# Investigation of Newly Synthesized Fluorinated Isatin-Hydrazones by *In Vitro* Antiproliferative Activity, Molecular Docking, ADME Analysis, and e-Pharmacophore Modeling

**DOI:** 10.1021/acsomega.4c03014

**Published:** 2024-06-05

**Authors:** Eyüp Başaran, Semiha Köprü, Senem Akkoç, Burçin Türkmenoğlu

**Affiliations:** † Department of Chemistry and Chemical Processing Technologies, Vocational School of Technical Sciences, Batman University, Batman 72060, Türkiye; ‡ Department of Chemistry, Faculty of Sciences, Erciyes University, Kayseri 38039, Türkiye; § Technology Research and Application Center, Erciyes University, Kayseri 38039, Türkiye; ∥ Department of Basic Pharmaceutical Sciences, Faculty of Pharmacy, Suleyman Demirel University, Isparta 32260, Türkiye; ⊥ Faculty of Engineering and Natural Sciences, Bahcesehir University, Istanbul 34353, Türkiye; # Department of Analytical Chemistry, Faculty of Pharmacy, Erzincan Binali Yildirim University, Erzincan 24002, Türkiye

## Abstract

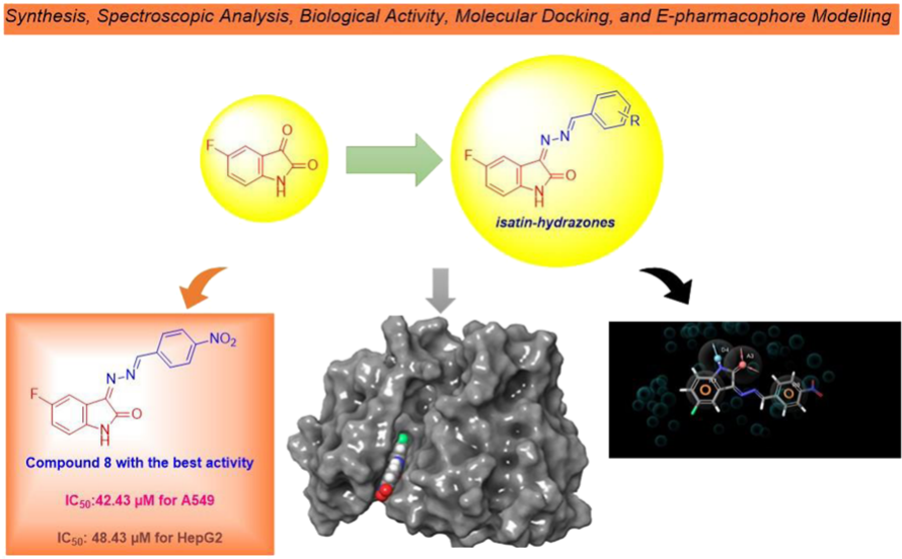

In this study, we investigated the *in vitro* antiproliferative activities and performed computational studies of newly synthesized fluorinated isatin-hydrazones. The chemical structures of the synthesized compounds were confirmed by FT-IR, 1D NMR (^1^H- and ^13^C NMR and APT), 2D NMR (HETCOR and HMBC), and elemental analysis. All compounds (**1**–**15**) were tested in human lung (A549) and liver (HepG2) cancer cell lines for 72 h. The compounds were screened against a healthy embryonic kidney cell line (HEK-293T) under the same conditions to determine their toxic effects. According to the results obtained, one of the compounds, in particular, compound **8** was effective at inhibiting the growth of cancerous cells, and its effects on both cancer cell lines were similar to IC_50_ values of 42.43 and 48.43 μM for A549 and HepG2, respectively. Compound **8**, which was determined to be the best anticancer agent *in vitro*, was chosen to interact with the target via molecular docking. This selected ligand (compound **8**) interacted with the targets 4HJO, 4ASD, 3POZ, and 7TZ7, and docked into the active sites. The docking score, Glide energy, and Glide emodel values were calculated and determined to be lower than those of the reference compound cisplatin. The pharmacokinetic properties, stability, and drug-likeness parameters of all designed compounds were estimated using SwissADME. Finally, the binding affinities of compound **8** for all four targets were calculated using the MM-GBSA method.

## 1 Introduction

Cancer is a leading cause of global mortality and is responsible for a significant number of deaths worldwide.^1^ Cancer is an overarching concept used to delineate a pathological condition marked by the unrestrained expansion of cells stemming from the perturbation or malfunction of regulatory signaling pathways that typically function within precise boundaries.^2^ Therefore, the World Health Organization anticipates that the number of individuals afflicted with cancer will increase to 22 million by 2030.^3^ Lung and liver cancer are malignant tumors with high mortality and the incidence of both types of cancer has increased rapidly in recent years^4,5^ Various diagnostic and therapeutic approaches have been employed for the aforementioned types of cancer up to the present time. Nevertheless, the survival rates of individuals afflicted with these malignancies are notably low.^6,7^ Lung cancer continues to be the primary contributor to mortality from malignant neoplasms, accounting for 1.8 million fatalities (18%), followed by colorectal (9.4%), liver (8.3%), gastric (7.7%), and mammary (6.9%) malignancies.^8^ The objective of the investigation in this field is to develop anticancer drugs that demonstrate substantial effectiveness while minimizing toxicity.

Hydrazones, which are employed in diverse domains of organic chemistry, are renowned for their versatility and are recognized as a significant category of compounds for new drug development.^9^ Hydrazones, which are regarded as a significant group of organic compounds, encompass C=N bonds that are conjugated with the lone pair of electrons of a functional nitrogen atom, and possess the structure R_1_R_2_C=NNH_2_^10,11^ Nitrogen atoms exhibit nucleophilic properties, whereas carbon atoms possess both electrophilic and nucleophilic characteristics.^12,13^ Hydrazone linkages in pharmaceutical materials have significant value in numerous transformations and have attracted great interest because of their many biological and clinical applications, such as anticancer, antimicrobial, antioxidant, antituberculosis, anti-inflammatory, antiviral, antiproliferative, and enzyme inhibitory activities.^14−24^

Isatin (1*H*-indole-2,3-dione) was known as a synthetic molecule until it was discovered in the fruits of the cannonball tree *Couroupita guianensis*.^25^ It occurs naturally in plants of the *Isatis* genus and in many organisms and acts as a metabolic derivative of adrenaline in humans.^26,27^ Isatins are a prominent group of heterocycles with fascinating biological properties. It is a molecule with extensive synthetic capabilities, holds a significant position in the field of medicinal chemistry, and serves as a precursor for numerous pharmacologically active substances.^28^ Various substituents on isatin nuclei have demonstrated a wide range of biological activities, including antiproliferative,^27,29^ enzymatic,^30^ anti-inflammatory,^31,32^ antiviral,^33^ and antimicrobial activities.^34,35^ Furthermore, there are ample reasons to investigate the anticancer activity of imino derivatives with isatin nuclei, as various studies have shown that the isatin nucleus has a strong anticancer effect against multiple cancer cell line.^36−38^

Molecular hybridization is a useful and common strategy in medicinal chemistry, consisting of combining different pharmacophoric moieties to create a new therapeutic molecule. Numerous studies and bioactivity results have been presented regarding the synthetic methodology, pharmacology, and biological evaluation of hydrazone hybrid molecules containing isatin nuclei.^38,39^ We have designed and synthesized a total of 14 fluorinated isatin-hydrazone hybrids, 12 of which are novel, by combining fluorinated isatin and arylhydrazine groups with biologically active structures in a single compound framework and adopting the molecular hybrid approach to develop potent anticancer agents. In addition, the drug discovery process is time-consuming and laborious. However, with the support of *in silico* approaches, the identification of better lead compounds and scaffolds for a target can be achieved.^40^ These approaches are ligand- and structure-based drug design methods. Pharmacophore modeling is also a technique that can help in the rapid prediction of hits. The combination of ligand-based pharmacophore models helps to achieve better productivity.^41^ Thus, better lead compounds can be designed with e-pharmacophores for specific targets. In the current study, both ligand-based (e-pharmacophore) and structure-based (docking, ADMET) approaches were combined.^42^

This study aimed to design and obtain newly synthesized fluorinated isatin-hydrazone compounds that are pharmacologically effective against cancer cells. New ligands have been proposed using multidisciplinary methods, including synthesis, structure elucidation, and *in vitro* and *in silico* approaches. For these purposes, all synthesized compounds and their structures were elucidated and examined *in vitro* against A549, HepG2, and HEK-293T cells. Computational studies applying computer-aided drug design were conducted to support the results. The Epidermal Growth Factor Receptor (EGFR), Vascular Endothelial Growth Factor Receptor 2 (VEGFR2), EGFR Kinase and PI3K α structures have been identified as targets known to be effective against cancer cells. In this manner, both experimental and computational results were obtained.

## 2 Results and Discussion

### 2.1 Chemistry

**Scheme 1:**
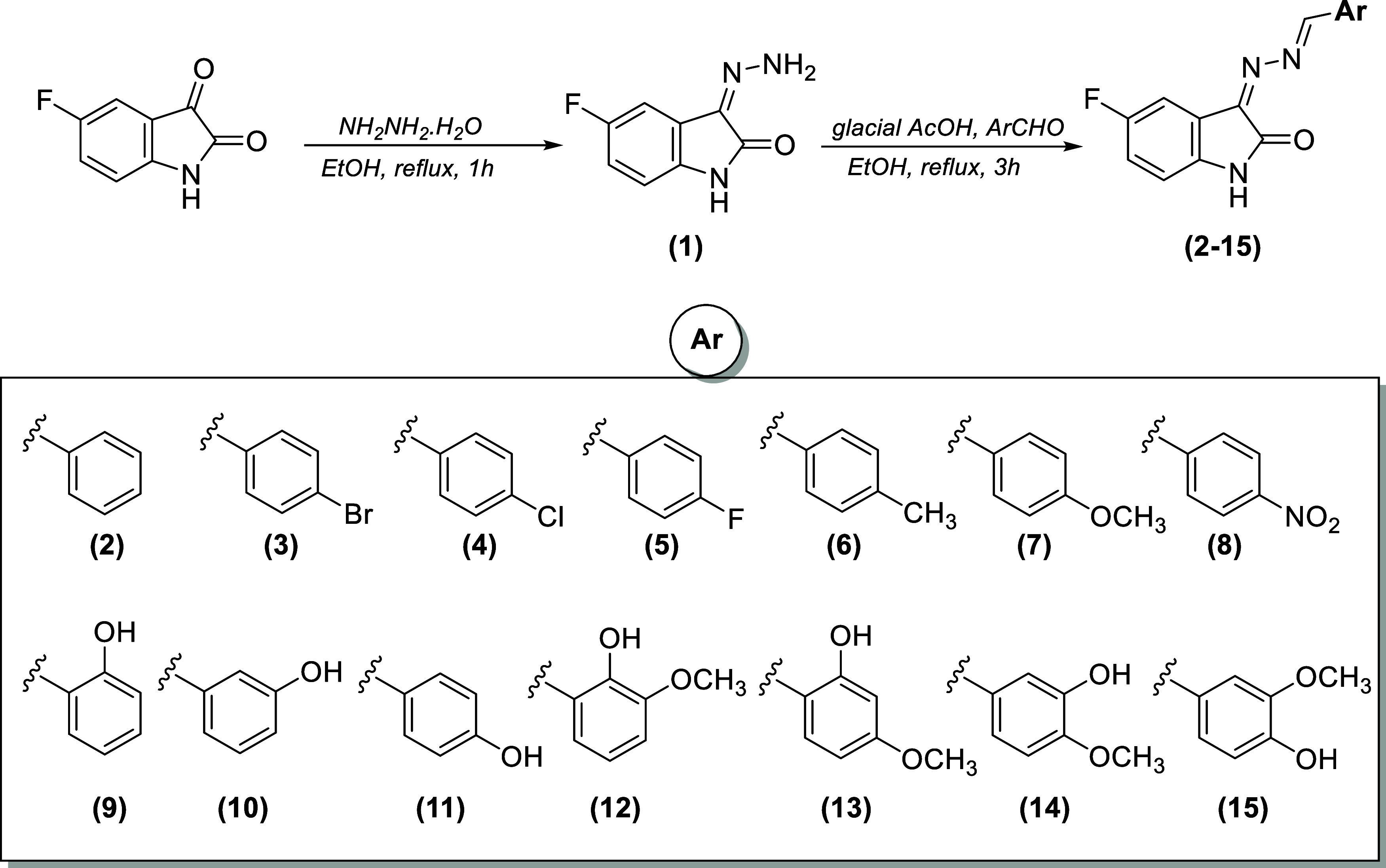
Synthesis of Fluorinated Isatin-Hydrazones (**2**–**15**)

As shown in Scheme 1, the newly synthesized fluorinated isatin-hydrazones **2**–**15** were prepared in 57–71% yield, and the structures of the target compounds were supported by spectral data, such as FT-IR, 1D and 2D NMR, and elemental analysis.

The IR spectra of compounds **2**–**15** were recorded exhibiting the amino (N–H) stretching band at 3243–3122 cm^–1^, carbonyl (C=O) stretching band at 1760–1721 cm^–1^, and azomethine (C=N) stretching band at 1625–1610 cm^–1^. The hydroxyl (O–H) stretching band in the structures of compounds **8**–**15** was confirmed at 3394–3201 cm^–1^. In the H NMR spectrum of compounds **2**–**15**, the NH proton in the indole ring was observed to be singlet resonant at 10.86–11.03 ppm. The azomethine CH=N proton, which indicated the formation of a hydrazone structure, was found to be singlet resonant at 8.62–9.01 ppm. The OH protons in the structures of compounds **8**–**15** were found to resonate at 9.64–12.29 ppm. The three protons of the methoxy (OCH_3_) group in compounds **7** and **12**–**15** were detected as singlet resonant at 3.82–3.88 ppm. The aromatic protons of the target compounds were observed to be resonant at 6.55 and 8.22 ppm as multiplet (m), triplet (t), doublet (d), double doublet (dd), and singlet (s). In the ^13^C NMR spectra for compounds **2**–**15** showed that the indole ring C=O carbon resonated at 164.65–165.32 ppm, while the azomethine C=N carbon resonated at 159.12–164.74 ppm. The aromatic carbons exhibited the expected peaks. The NMR data from the APT, HMBC, and HETCOR spectra of exemplary compounds **7** and **14** are described in more detail.

**Figure 1:**
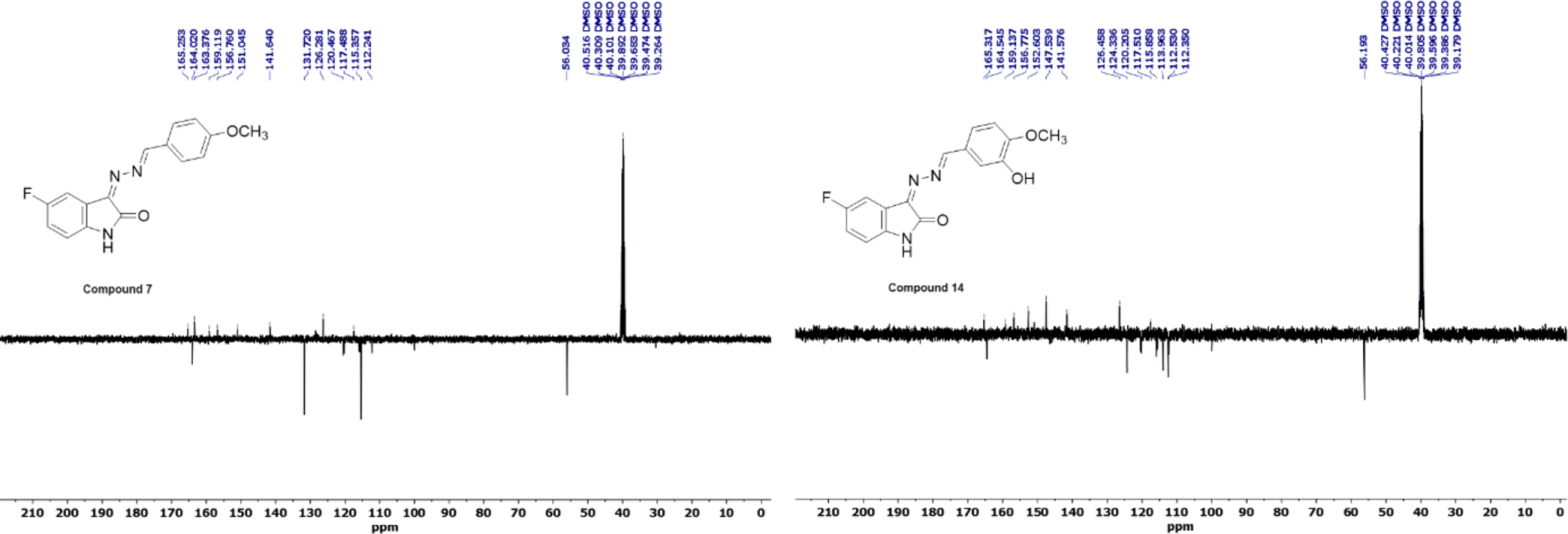
APT spectra of compounds **7** and **14**.

In the APT spectrum of compound **7**, while the peak of the −OCH_3_ group at the *para* position appears at 56.03 ppm, that of the azomethine group −CH appears at 164.02 ppm, the aromatic −CH– peaks for the fluorinated indole ring appear at 112.33, 120.46, and 115.94 ppm, and the −CH peaks of the phenyl ring to which the *p*-OCH_3_ group is attached appear at 115 and 131 ppm and are oriented in the negative direction, the indole ring carbonyl group is oriented at 165.25 ppm, the quaternary carbon attached to the 5-fluorine position is oriented at 159.12 ppm, quaternary carbon at 163.38 ppm to which the methoxy group is attached, and the other deprotonated carbons are oriented in the positive direction. Also, when we examined the spectrum of compound **14**, while the peak of the −OCH_3_ group at the *p*-position appears at 56.19 ppm, the −CH peak of the azomethine group appears at 164.55 ppm, the −CH peaks of the aromatic ring for the fluorinated indole ring appear at 112.35, 120.43, and 115.86 ppm and the −CH peaks of the aromatic phenyl ring to which the *p*-OCH_3_ is attached appear at 113.96, 124.33, and 112.35 ppm and are oriented in the negative direction, the indole ring carbonyl group is oriented at 165.31 ppm, the quaternary carbon attached to the 5-fluorine position is oriented at 159.18 ppm, quaternary carbon at 152.67 ppm to which the methoxy group is attached and the other deprotonated carbons are oriented in the positive direction. The APT spectra of compounds **7** and **14** are shown in Figure 1.

**Figure 2:**
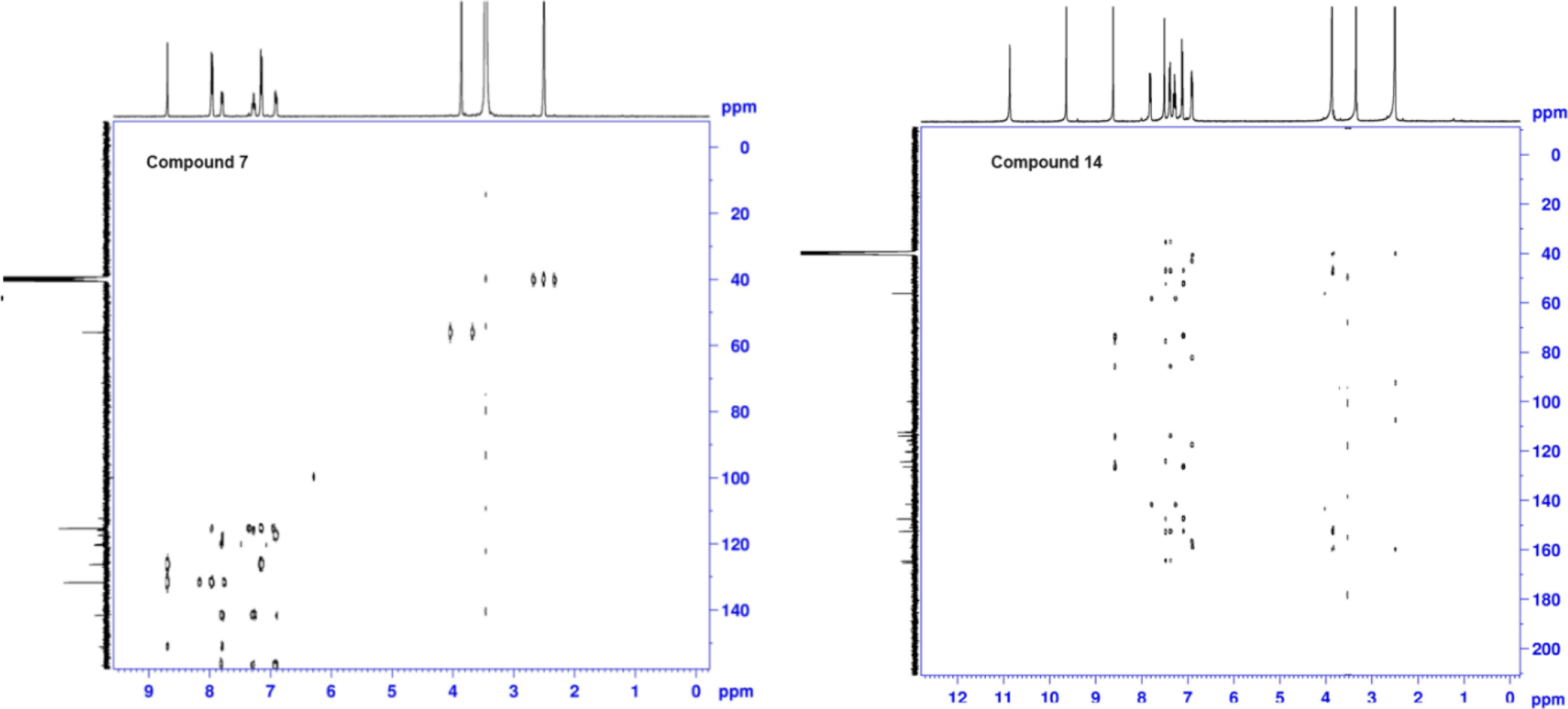
HMBC spectra of compounds **7** and **14**.

In the HMBC spectrum of compound **7**, the azomethine-CH peak at 8.7 ppm was correlated with the quaternary carbon at 126.28 ppm through 1 bond and with the aromatic −CH carbon at 131.7 ppm through 2 bonds. The aromatic ring proton at 7.8 ppm was paired with 115 ppm −CH over 1 bond and 163.38 ppm quaternary carbon over 2 bonds. The indole ring is correlated with −CH at 6.9 ppm, with the deprotonated carbon with an F bond over 1 bond at 159.12 ppm, with the deprotonated carbon at 156.76 ppm over 2 bonds, and with the 5-ring –C=N of the indole ring with 5 bonds at 141.64 ppm. At 7.2 ppm, the indole ring −CH peak matched the deprotonated carbon with an F bond over 1 bond at 159.12 ppm, the carbon over 2 bonds at 112.23 ppm, the deprotonated carbon over 1 bond at 117.49 ppm, and the deprotonated carbon over 2 bonds at 156.76 ppm. At 7.8 ppm, the indole −CH peak, with F-bonded deprotonated carbon over 2 bonds, at 159.12 ppm, with −CH carbon over 1 bond at 112.23 ppm, it is paired with the deprotonated carbon at 117.49 ppm over 2 bonds, with the deprotonated carbon at 156.76 ppm over 1 bond, and with the carbonyl carbon at 165.32 over 3 bonds. Moreover, when we examined the HMBC spectrum of compound **14**, the azomethine −CH peak at 8.62 ppm is correlated with the quaternary carbon at 126.46 ppm through 1 bond and with the aromatic −CH carbon at 113.97 and 124.28 ppm through 2 bonds. The aromatic ring proton at 7.1 ppm is paired with 124.28 ppm of aromatic −CH over 1 bond and 152.77 quaternary carbon over 1 bond, and 147.54 ppm of the −OH group is paired with the deprotonated carbon over 2 bonds. The indole ring is correlated with −CH at 6.9 ppm, with the deprotonated carbon with an F bond over 1 bond at 156.77 ppm, with the deprotonated carbon at 152.60 ppm over 2 bonds, and with the 5-ring −C=N of indole with 5 bonds at 141.57 ppm. At 7.2 ppm the indole ring-CH peak matched the deprotonated carbon with F bond over 1 bond at 156.77 ppm, the carbon over 2 bonds at 112.23 ppm, the deprotonated carbon over 1 bond at 115.5 ppm, and the deprotonated carbon over 2 bonds at 152.60 ppm. At 7.8 ppm indole ring-CH peak, with F-bonded deprotonated carbon over 2 bonds, at 156.77 ppm and −CH carbon over 1 bond at 112.23 ppm, it is paired with the deprotonated carbon at 119 ppm over 2 bonds, with the deprotonated carbon at 152.6 ppm over 1 bond, and with the carbonyl carbon at 165.32 over 3 bonds. The HMBC spectra of compounds **7** and **14** are shown in Figure 2.

**Figure 3:**
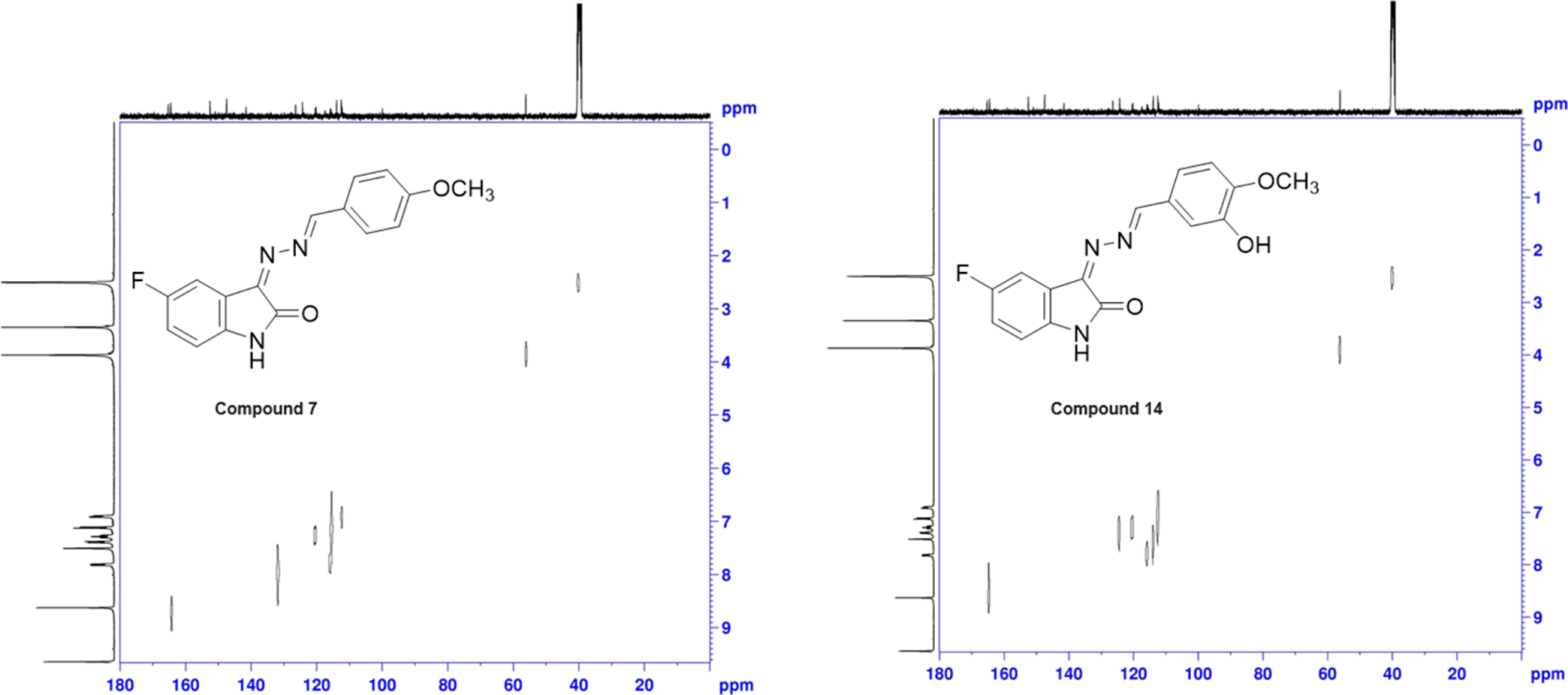
HETCOR spectra of compounds **7** and **14**.

In the HETCOR spectrum of compound **7**, the −OCH_3_ peak at 56.03 ppm due to the aromatic ring in the ^13^C NMR spectrum matched with the peak at 3.86 ppm in ^1^H NMR. Phenyl ring-CH peaks were observed coincidentally at 7.16 and 7.95 ppm in the ^1^H NMR spectrum. The 7.16 and 7.95 ppm −CH peaks are correlated with the 115.35 and 131.7 ppm peaks, respectively. The indole ring-CH peaks were observed at 6.9, 7.2, and 7.8 ppm, and a match was observed with the peaks at 112.33, 120.46, and 115.94 ppm in the HETCOR. The azomethine-CH peak at 8.7 ppm is correlated at 164.02 ppm. Additionally, when we examined the HETCOR spectrum of compound **14**, the −OCH_3_ peak at 56.19 ppm due to the aromatic ring matched the peak at 3.87 ppm. Phenyl ring-CH peaks were observed at 7.1, 7.3, and 7.5 ppm. These peaks were observed to match the peaks of 112.35, 124.28, and 113.97 nm in the spectrum. The indole ring-CH peaks were observed at 6.9, 7.2, and 7.8 ppm, and a match was observed with the peaks of 112.23, 112.56, and 115.50 ppm in the HETCOR. The azomethine-CH peak at 8.62 ppm correlated to 164.55 ppm. The HETCOR spectra of compounds **7** and **14** are shown in Figure 3.

### 2.2 Cytotoxic Activity Studies

Compounds (**1**–**15**) were screened in two cancer and normal cell lines at concentrations of 200, 100, 50, and 25 μM for 72 h. The results are presented in Table 1.

**Table 1:** IC_50_ Results for Compounds against Human Cell Lines

	IC_50_ (μM)		
compounds	A549^a^	HepG2^b^	HEK-293T^c^
**1**	>200	>200	>200
**2**	>200	>200	>200
**3**	186.20	>200	24.99
**4**	>200	>200	29.34
**5**	>200	107.90	192.80
**6**	>200	>200	139.60
**7**	>200	152.90	197.40
**8**	42.43	48.43	6.04
**9**	>200	>200	142.00
**10**	179.90	166.30	87.03
**11**	>200	>200	>200
**12**	149.70	>200	>200
**13**	>200	>200	>200
**14**	115.00	>200	>200
**15**	>200	>200	>200
**cisplatin**	4.19	N.T.^d^	7.48

a A549: human lung epithelial carcinoma cell line.

b HepG2: human liver epithelial carcinoma cell line.

c HEK-293T: human normal embryonic kidney cell line.

d N.T.: not tested.

All synthesized compounds (**1**–**15**) were tested in a lung cancer cell line after incubation for 72 h of incubation. According to the results obtained, only five of the 15 compounds tested (**3**, **8**, **10**, **12**, and **14**) were found to have a cytotoxic effect on the incubation time and concentration studied. It was determined that the other ten compounds (**1**, **2**, **4**, **5**, **6**, **7**, **9**, **11**, **13**, and **15**) were not active against this cell line and did not inhibit cell growth. In the synthesized series of fluorinated isatin-hydrazone derivatives, compound **8**, containing the 4-nitrobenzylidene group, exhibited important lung cell growth inhibition with an IC_50_ value of 42.43 μM (Table 1). The second most effective compound in the series was compound **14**, which contained a 3-hydroxy-4-methoxybenzylidene group in its structure with an IC_50_ value of 115.00 μM. Although compound **12**, which contains a 2-hydroxy-3-methoxybenzylidene group in its structure, is the third most effective compound, 149.70 μM, it appears to have a moderate effect on inhibiting the growth of lung cells.

All compounds were tested *in vitro* against the liver cancer cell line and the second cancer cell line in which the prepared compounds (**1**–**15**) were tested. Four of the 15 compounds (**5**, **7**, **8**, and **10**) tested showed cytotoxic effects against this cell line. While compound **5** containing a 4-fluorobenzylidene group in its structure and compound **7** containing a 4-methoxybenzylidene group in its structure were inactive against the A549 cell line, they showed an effect in the HepG2 cell line with an IC_50_ value of 107.90 and 152.90 μM, respectively. Compounds **8** and **10**, which contained 4-nitrobenzylidene and 3-hydroxybenzylidene groups in their structure, respectively, were found to have cytotoxic effects against both cancer cell lines by using the MTT assay. Similar to the A549 cell line, compound **8** showed the highest cytotoxic effect on the HepG2 cell line, with an IC_50_ value of 48.43 μM. As shown in Table 1, the IC_50_ values of compound **8** were very similar in both cell lines. In other words, there was a slight difference in the activity of this compound in the A549 and HepG2 cell lines. The lower IC_50_ value of compound **8** for normal cells than for cancer cells suggests that the compound may cause significant toxicity in healthy tissues, which may limit its clinical use. In drug development, minimizing damage to healthy tissues is vital for improving patient outcomes and the quality of life. Therefore, compound selectivity is important in the field of oncology. Different derivatives of compound **8** should be prepared in future studies to increase the cytotoxic effect against cancer cells while minimizing the cytotoxicity toward normal cells.

**Figure 4:**
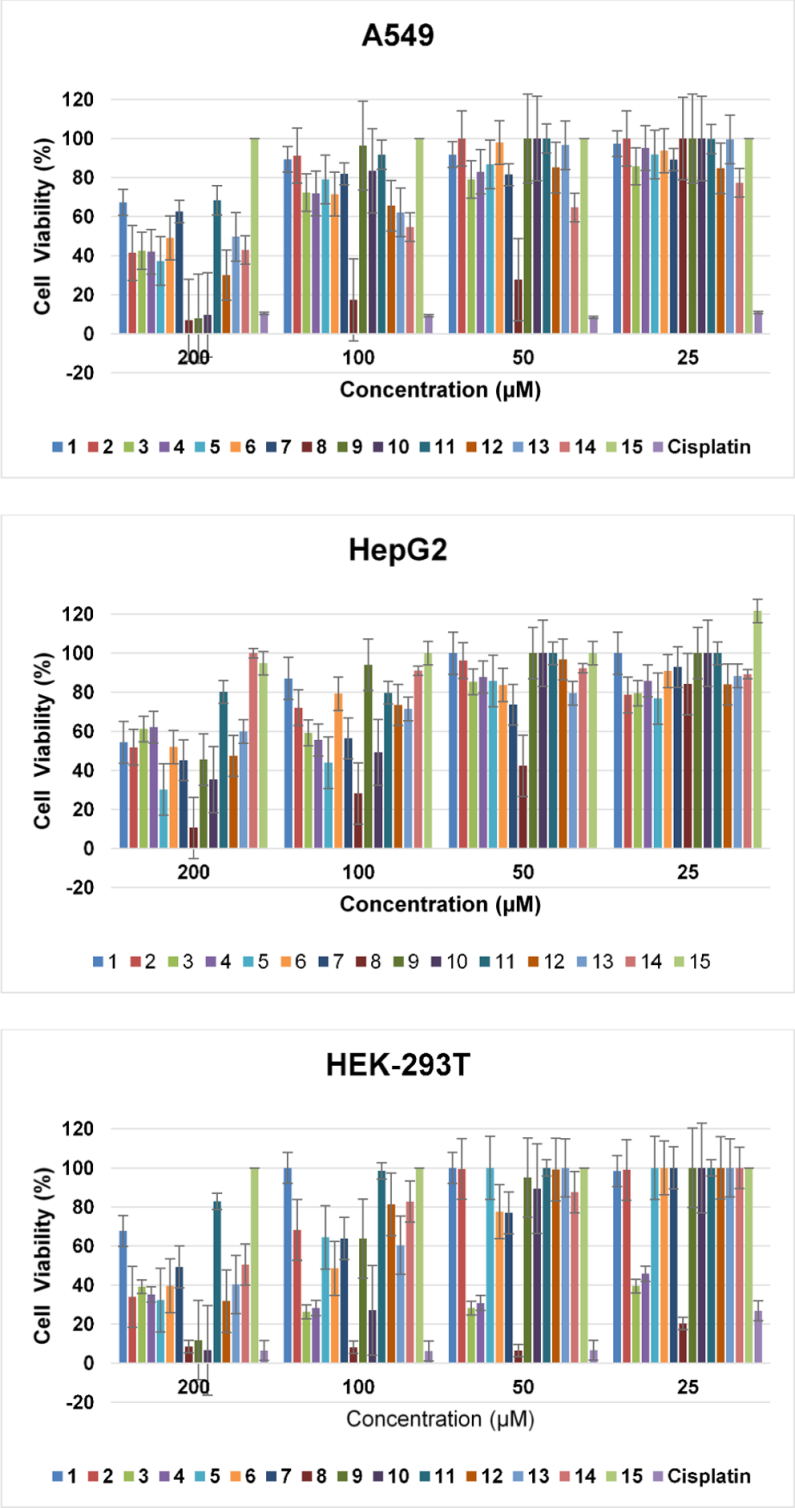
Changes in the viability of A549, HepG2, and HEK-293T cells depending on the concentrations of the tested compounds.

Eight of the 15 compounds (**3**–**10**) tested in a healthy cell line were found to have toxic effects. It has been determined that some compounds (**4**, **6**, and **9**) that do not have a cytotoxic effect against both cancerous cell types do not show selectivity against healthy cells and have a toxic effect on HEK-293T cells for 72 h. The change in substituents in the compounds coded **1**, **2**, and **11**–**15** did not affect the toxic effect of the compounds on healthy cells, and all of them were found to have an IC_50_ value greater than 200 μM. Compounds **3** and **4**, which contained 4-bromobenzylidene and 4-chlorobenzylidene groups in their structure, respectively, were observed to have highly toxic effects. Compound **8**, which had a highly cytotoxic effect on cancerous cells, did not show selectivity toward healthy cells and had a higher toxic effect than the positive control drug cisplatin (IC_50_ value of 6.04 μM), which is a well-known chemotherapeutic drug that has been used for decades in the treatment of various types of cancer, including liver and lung cancer. Compound **5** did not show high toxicity against HEK-293T cells and caused only slight growth inhibition at the highest tested doses of 100 and 200 μM. As shown in Figure 4, compound **8** caused high cell growth inhibition, even at the lowest tested concentration of 25 μM.

The selectivity indices (SI) of the compounds were calculated according to the formula below.
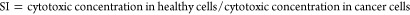


Considering this formula, when the HEK-293T IC_50_ value was divided by the A549 IC_50_ value for each compound, the selectivity index was obtained as follows: 1, 1, 0.13, 0.14, 0.96, 0.69, 0.98, 0.14, 0.71, 0.48, 1, 1.33, 1, 1.73, and 1 for compounds **1**–**15**, respectively. Likewise, these values were calculated as 1, 1, 0.12, 0.14, 1.78, 0.69, 1.29, 0.12, 0.71, 0.52, 1, 1, 1, 1, and 1 for compounds **1**–**15** in HepG2, respectively. According to these results, compound **14** showed greater selectivity against the A549 cell line, and compound **5** showed higher selectivity against the HepG2 cell line.

### 2.3 Computational Studies

#### 2.3.1 Predicting Drug-Likeness and Physicochemical Properties with SwissADME

Currently, the first step in computational studies of synthesized lead compounds is to examine the ADME predictions.^43^ If these compounds have good ADME properties, they can be presented as drug-like drug candidates. In this study, ADME-estimated values were examined computationally in terms of pharmacokinetics. Thus, it can be used as a tool to reduce failures before starting *in vivo* experiments.

ADME analysis was performed online using the SwissADME server. Drug–drug interactions generally result from the major cytochrome P450 (CYP) inhibition. Small molecule inhibitors of CYP3A4, 2D6, 2C19, 2C9, and 1A2 have different physicochemical and structural properties.^43^ The inhibitory properties of human Cytochrome P450 were examined using ADME studies and are presented in Table 2.

**Table 2:** SwissADME Values for Compounds **1**–**15**

compounds	CYP1A2 inhibitor	CYP2C19 inhibitor	CYP2C9 inhibitor	CYP2D6 inhibitor	CYP3A4 inhibitor	BBB permeant	GI absorption	P-gp substrate	log *K*_p_ (skin permeation
**1**	no	no	no	no	no	no	high	no	–6.53 cm/s
**2**	yes	yes	no	no	no	yes	high	no	–5.99 cm/s
**3**	yes	yes	yes	no	no	yes	high	no	–5.98 cm/s
**4**	yes	yes	yes	no	no	yes	high	no	–5.75 cm/s
**5**	yes	no	no	no	no	yes	high	no	–6.03 cm/s
**6**	yes	yes	no	no	no	yes	high	no	–5.82 cm/s
**7**	yes	yes	yes	no	no	yes	high	no	–6.20 cm/s
**8**	yes	no	no	no	no	no	high	no	–6.39 cm/s
**9**	yes	no	no	no	no	yes	high	no	–6.35 cm/s
**10**	yes	no	no	no	no	yes	high	no	–6.35 cm/s
**11**	yes	no	no	no	no	yes	high	no	–6.35 cm/s
**12**	yes	no	no	no	yes	no	high	no	–6.54 cm/s
**13**	yes	no	no	no	yes	no	high	no	–6.54 cm/s
**14**	yes	no	no	no	yes	no	high	no	–6.54 cm/s
**15**	yes	no	no	no	yes	no	high	no	–6.54 cm/s

Blood–Brain Barrier (BBB) permeability and gastrointestinal absorption score (GI absorption score) values were also calculated for 15 compounds and are presented in Table 2. The pharmacokinetic properties are listed in Table 2.

Table 2 shows that some of the synthesized compounds are BBB permeable, and some are not. According to the *in vitro* results obtained, compound **8**, which is the best compound, has no BBB permeability in the data presented in Table 2, which is a desirable situation, its log *K*_p_ value is −6.39 cm/s, its GI absorption is high, it does not have a P-gb substrate, and it has CYP1A2 inhibitory properties. According to the data presented in Table 2, compound **8** had good pharmacokinetic properties.

Drug-likeness and pharmacokinetic ADMET properties were examined to predict whether all of the synthesized compounds would be new precursor compounds. It was observed that all compounds presented in Table 3 obey the Lipinski, Veber, and Egan rule, while only compound **1** violated the Ghose and Muggen rule. In addition, all compounds were evaluated to have a bioavailability value of 0.55, which indicates high bioavailability and permeability, according to Lipinski’s rule of five.

**Table 3:** Predicted Drug Similarities and Bioavailability Values of Compounds **1**–**15** Based on Lipinski, Ghose, Veber, Egan, and Muegge Rules

compounds	Lipinski	Ghose	Veber	Egan	Muegge	bioavailability score
**1**	yes	no	yes	yes	no	0.55
**2**	yes	yes	yes	yes	yes	0.55
**3**	yes	yes	yes	yes	yes	0.55
**4**	yes	yes	yes	yes	yes	0.55
**5**	yes	yes	yes	yes	yes	0.55
**6**	yes	yes	yes	yes	yes	0.55
**7**	yes	yes	yes	yes	yes	0.55
**8**	yes	yes	yes	yes	yes	0.55
**9**	yes	yes	yes	yes	yes	0.55
**10**	yes	yes	yes	yes	yes	0.55
**11**	yes	yes	yes	yes	yes	0.55
**12**	yes	yes	yes	yes	yes	0.55
**13**	yes	yes	yes	yes	yes	0.55
**14**	yes	yes	yes	yes	yes	0.55
**15**	yes	yes	yes	yes	yes	0.55

Analysis of the computational physicochemical properties of the synthesized compounds offers the idea of evaluating drug-like properties in advance in drug discovery research. The ADME properties of all compounds synthesized in this study are presented in Table 4. The molecular mass must be less than 500 Da, log *P* value must be less than 5, the number of hydrogen bond donors must be less than 5, the number of hydrogen bond acceptors must be less than 10, and TPSA values must be more than 20 A^2^ and less than 130 A^2^.^44^ Considering these necessary data, it was determined that all compounds presented in Table 4 complied with all of these rules.

**Table 4:** *In Silico* ADME Properties of Compounds **1**–**15**

compounds	MW (g/mol)	log *P*	HBD	HBA	TPSA (Å^2^)
**1**	179.15	0.88	2	3	67.48
**2**	267.26	2.70	1	4	53.82
**3**	346.15	3.34	1	4	53.82
**4**	301.70	3.25	1	4	53.82
**5**	285.25	3.02	1	5	53.82
**6**	281.28	3.04	1	4	53.82
**7**	297.28	2.71	1	5	63.05
**8**	312.26	2.15	1	6	99.64
**9**	283.26	2.30	2	5	74.05
**10**	283.26	2.31	2	5	74.05
**11**	283.26	2.28	2	5	74.05
**12**	313.28	2.33	2	6	83.28
**13**	313.28	2.28	2	6	83.28
**14**	313.28	2.32	2	6	83.28
**15**	313.28	2.35	2	6	83.28
**recommend values**	≤500	≤5	≤5	≤10	20 Å^2^ < TPSA < 130 Å^2^

Considering all the data in Tables 2–4 in the estimation of drug similarity and physicochemical properties section of our study using SwissADME, the physicochemical and pharmacokinetic properties of the compounds were calculated. It can be said that these estimated data are suitable lead molecule candidates and comply with all of the rules, especially for compound **8**.

#### 2.3.2 Molecular Docking and Binding Free Energy Calculations

Molecular docking studies have been conducted to support cytotoxic studies in cancer cell lines. When the molecular docking results were examined in terms of binding scores (Table 5), it was determined that they had better values than the reference compound cisplatin. In Table 5, compound **8** interacts with four different targets *in silico*, and the binding modes of compound **8** and cisplatin for each target are also presented.

**Table 5:** Binding Modes of Compound **8** and Cisplatin with Targets 4ASD, 3POZ, 4HJO, and 7TZ7

PDB ID	compound	H-bond	π-π stacking	π-cation	salt bridge	docking score	glide energy	glide emodel	Δ*G*_bind_
4ASD^a^	**8**	Cys919			Lys868 Glu885	–9.722	–50.164	–71.143	–62.74
	**cisplatin**	Hie1026 Ile1025				–2.984	–14.881	–16.259	47.33
3POZ^b^	**8**	Asp855				–7.178	–43.407	–56.099	–53.68
	**cisplatin**	Asp855 Thr854				–3.170	–15.161	–12.342	55.63
4HJO^c^	**8**	Met769			Lys704	–6.463	–41.083	–59.831	–48.82
	**cisplatin**	Thr766	Lys721			–2.972	–9.065	–11.268	51.80
7TZ7^d^	**8**	Tyr836 Ash810		Lys802 Tyr836		–6.296	–36.071	–50.720	–30.13
	**cisplatin**	Asn920 Asp933				–2.188	–9.559	–10.230	34.04

a Crystal structure of VEGFR2.

b Crystal structure of EGFR Kinase

c Crystal structure of the inactive EGFR.

d Crystal structure of PI3K α.

**Figure 5:**
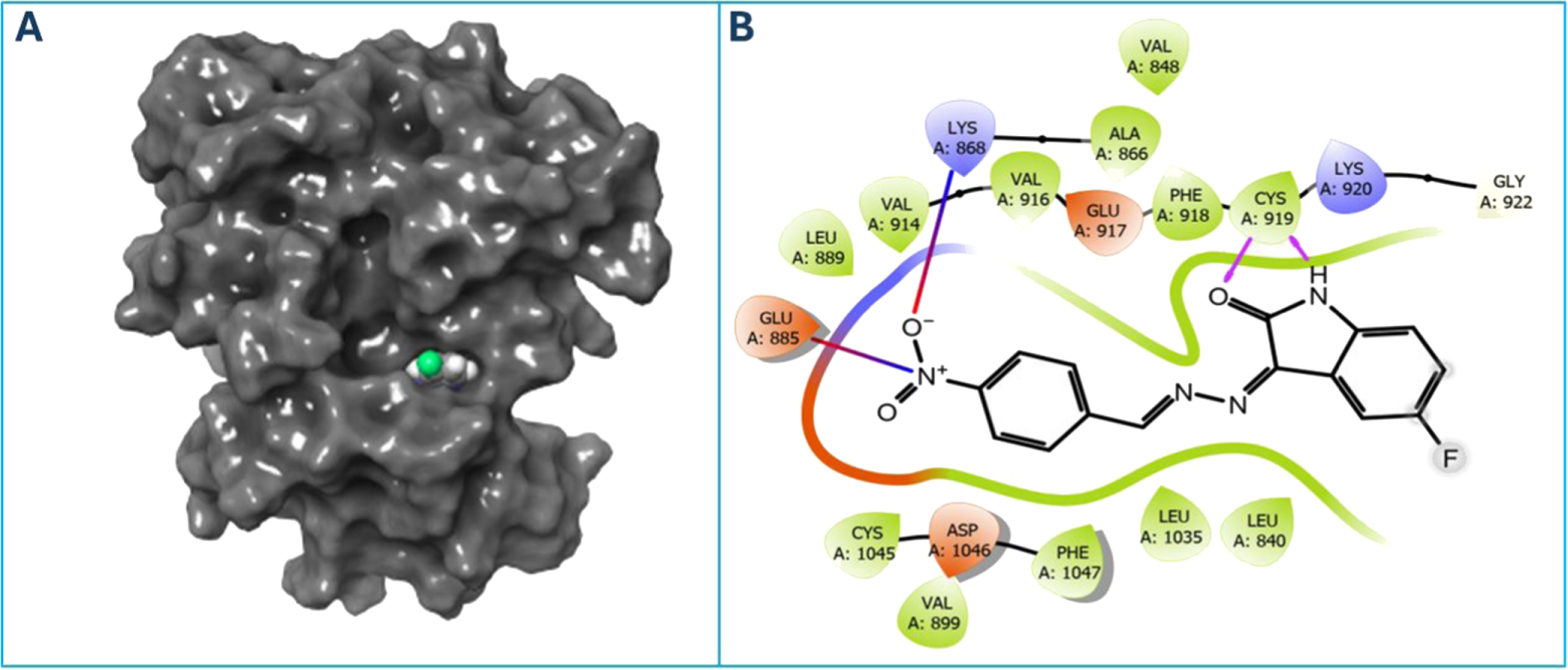
(A) 3D diagram for the interaction of compound **8** with VEGFR2 (PDB ID:4ASD). (B) 2D diagram of compound **8** with VEGFR2 (PDB ID:4ASD).

As shown in Table 5, the docking score of compound **8** for the VEGFR2 target was −9.722 kcal/mol. There is a hydrogen bond with the Cys919 amino acid residue in the 4ASD complex with compound **8** and a salt bridge interaction with the Lys868 and Glu885 amino acid residues. The 2D and 3D interactions between 4ASD and compound **8** are shown in Figure 5. In Figure 5A, compound **8** was docked to the active binding site of the 3D target.

**Figure 6:**
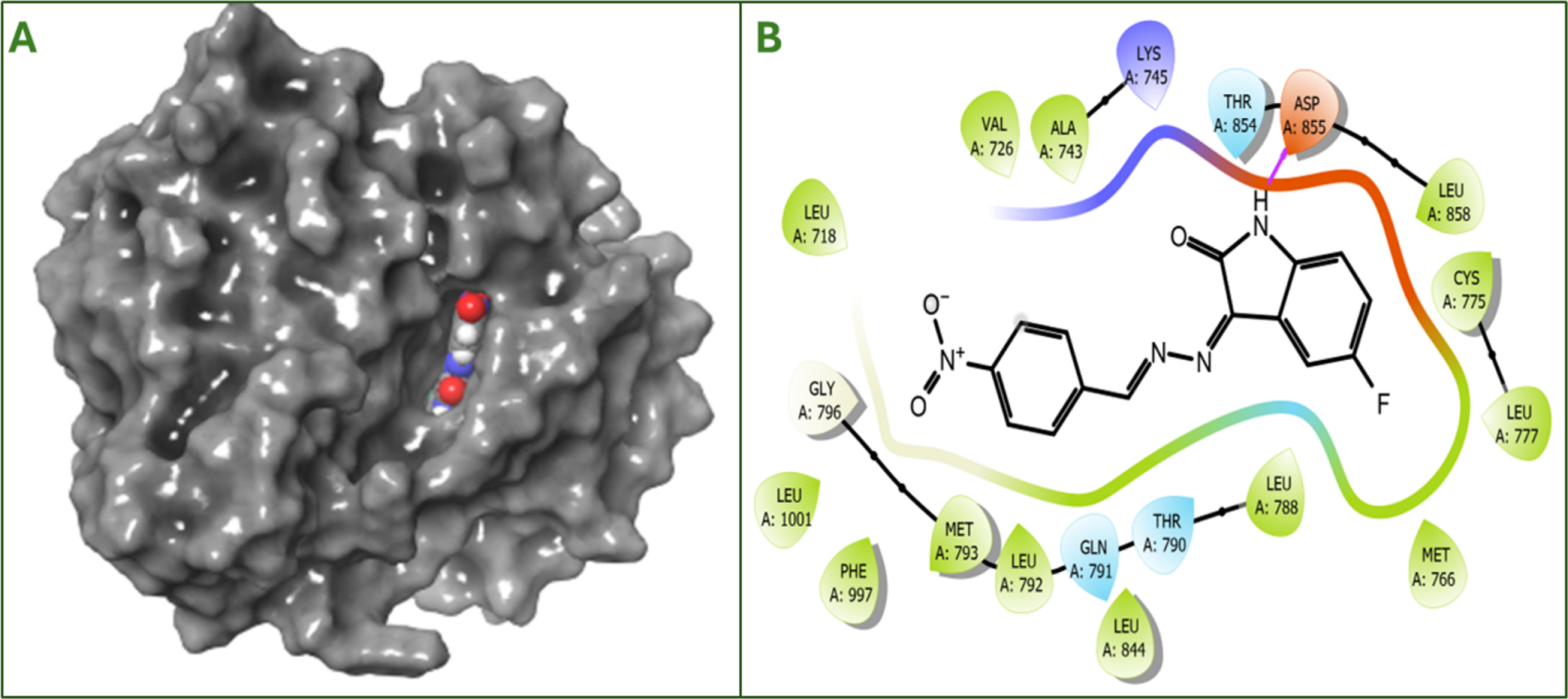
(A) 3D diagram for the interaction of compound **8** with EGFR TK (PDB ID:3POZ). (B) 2D diagram of compound **8** with EGFR TK (PDB ID:3POZ).

The crystal structure of the second target EGFR TK (Table 5) was 3POZ. The interaction results of both cisplatin and compound **8** with 3POZ are presented in Table 5. The docking score of compound **8**-3POZ complex according to molecular docking is −7.178 kcal/mol, and the free binding energy value is −53.68 kcal/mol. Here, too, it can be said that it has a binding mode that is better than that of cisplatin. Additionally, Figure 6A shows that compound **8** docked to the correct region in the 3D diagram of EGFR TK. In the 2D interaction diagram in Figure 6B, it was determined that hydrogen bonding occurred with amino acid Asp855.

**Figure 7:**
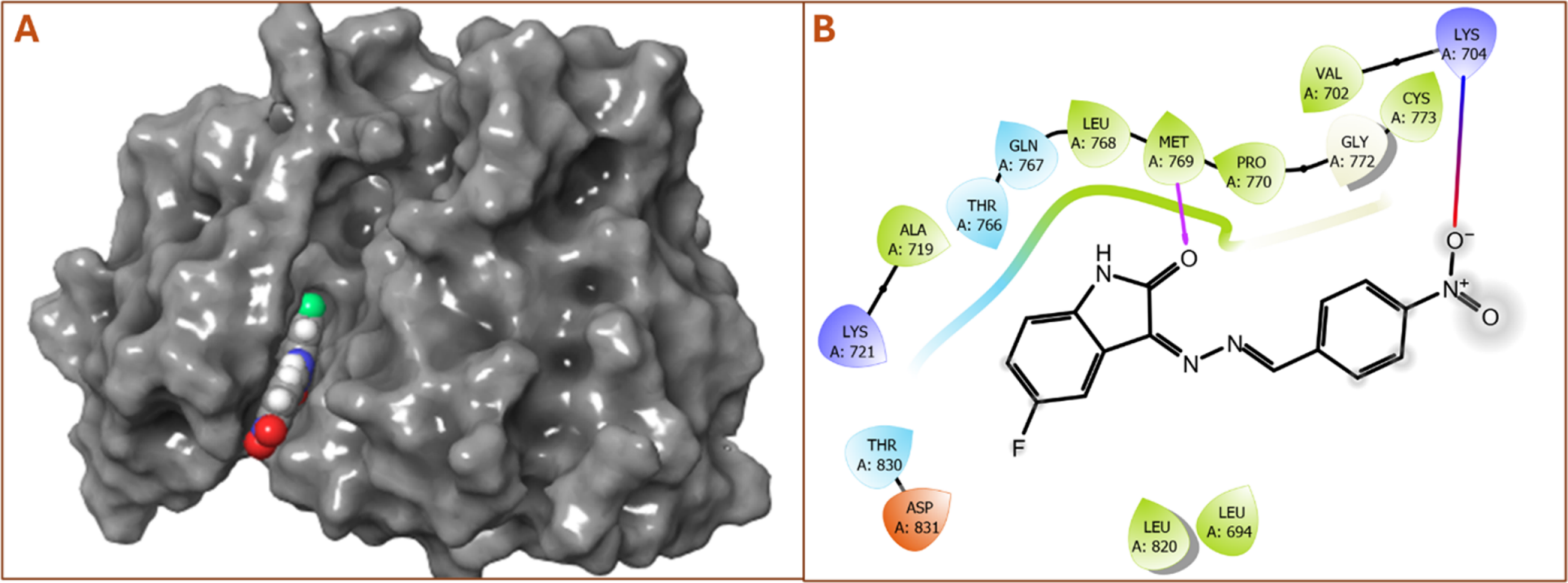
(A) 3D diagram for the interaction of compound **8** with EGFR (PDB ID:4HJO). (B) 2D diagram of compound **8** with EGFR (PDB ID:4HJO).

In Figure 7, the interaction of EGFR, one of the most important receptors in cancer, is shown in both 2D and 3D. As shown in Table 5, according to the values calculated according to molecular docking of the EGFR-compound **8** complex, the docking score value was −6.463 kcal/mol, the Glide energy value was −41.083 kcal/mol, the Glide emodel value was −59.831 kcal/mol, and the free binding energy value was −48.82 kcal/mol. The amino acid residues in the binding mode of the compound **8**-4HJO complex are shown in both Table 5 and Figure 7. Figure 7A shows how compound **8**, which binds to the active binding site of the target, is docked. In the 2-dimensional interaction diagram in Figure 7B, it was determined that there was a hydrogen bond interaction with the amino acid Met769 and a salt bridge interaction with the amino acid Lys704, which are important interactions for the inhibition of EGFR.

The interaction between cisplatin and compound **8** with the final target, PI3K α, was investigated *in silico*. Both bonding modes and bonding parameter values were calculated and are presented in Table 5. The docking score of compound **8** with the 7TZ7 target was −6.296 kcal/mol, and the binding energy value of the resulting complex is −30.13 kcal/mol. As shown in Table 5, the reference compound cisplatin, which interacts with the 7TZ7 target, had lower binding parameter values in compound **8**.

**Figure 8:**
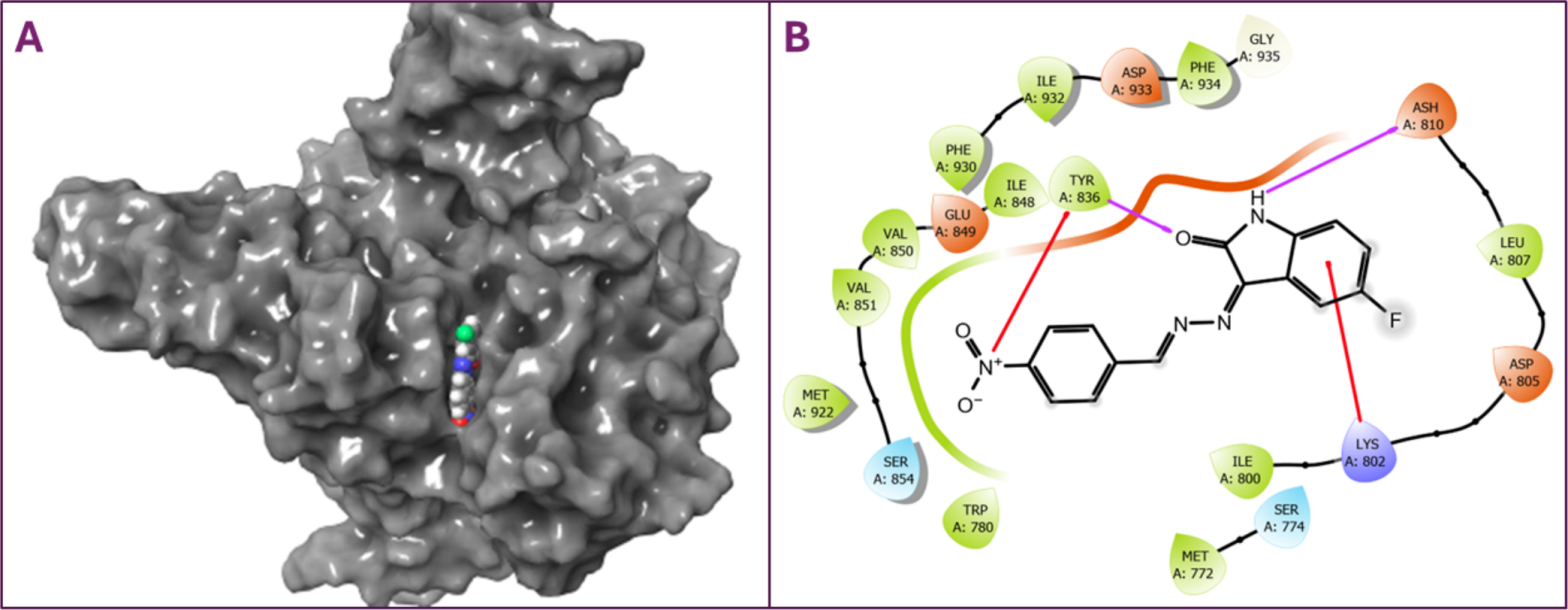
(A) 3D diagram for the interaction of compound **8** with PI3K α (PDB ID:7TZ7). (B) 2D diagram of compound **8** with PI3K α (PDB ID:7TZ7).

It has been understood that the ligand is located in the interaction region of the target in the binding mode presented in the 2D and 3D interaction diagrams in Figure 8. In the two-dimensional interaction diagram in Figure 8B, it is presented that there is a hydrogen bond interaction with amino acid residues Tyr836 and Ash810 and a cation–π bond interaction with amino acids Tyr836 and Lys802.

#### 2.3.3 Determination of e-Pharmacophores

The subsections of computer-aided drug design are ligand-based and structure-based drug designs. In this study, pharmacophore groups within the target of the most active compound were determined to support molecular docking. Pharmacophore modeling and structure-based protein–ligand docking are now considered integral parts of drug discovery in the current scientific computational studies. Screening of lead compounds by the e-pharmacophore method has the advantages of both ligand- and structure-based approaches by producing energetically optimized, structure-based pharmacophores (https://www.schrodinger.com/science-articles/e-pharmacophores).

**Figure 9:**
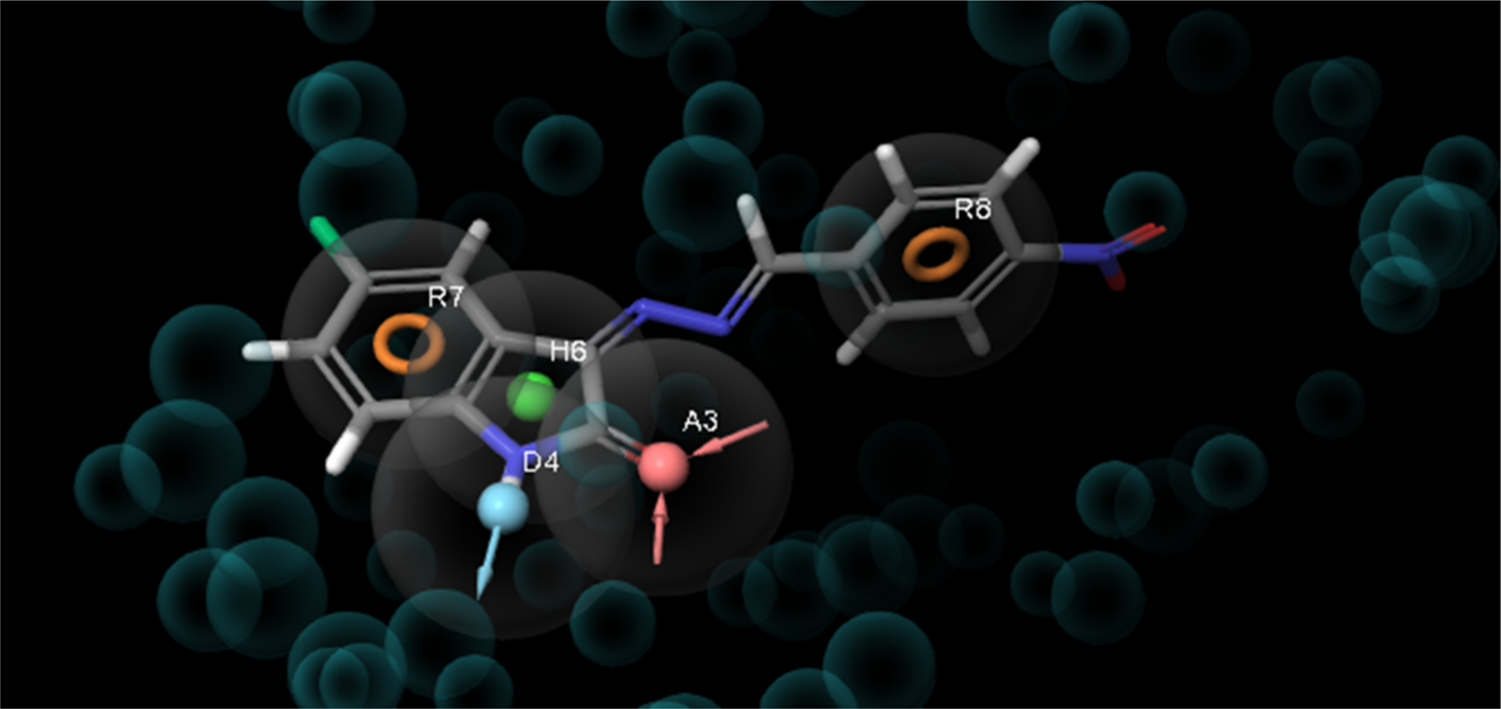
Generated pharmacophore hypothesis of VEGFR2 complexed with aligned compound **8** as a reference.

The phase module of compound **8** that interacted with the targets in molecular docking was used to determine an electronic pharmacophore (e-pharmacophore) model. This model was developed using compound **8**, which binds to VEGFR2, and its structural properties were determined (Figure 9). The e-pharmacophore model was designed to distinguish suitable sites within the active sites of innovative inhibitors.^45,46^

Known for their speed, e-pharmacophore screening methods serve as ideal tools for the preliminary screening of comprehensive molecular libraries. Compounds selected after screening are then subjected to a more sensitive, albeit slower method, such as Glide SP or other comprehensive techniques, to accurately determine the binding free energy. A structure-based pharmacophore model using VEGFR2 was constructed to provide insights into inhibitor binding to each target (Figure 9). Considering the complex formed as a result of the molecular docking of Glide XP, pharmacophore mapping was performed based on the structural and energy data between the target and the ligand.

The e-pharmacophore hypothesis, created for the active region of the 4ASD crystal structure, consists of five pharmacophores. The five pharmacophores presented in Figure 9 include two aromatic rings (R7 and R8), one hydrogen bond donor (D4), one hydrogen bond acceptor (A3), and one hydrophobic (H6) interaction.^47,48^

**Figure 10:**
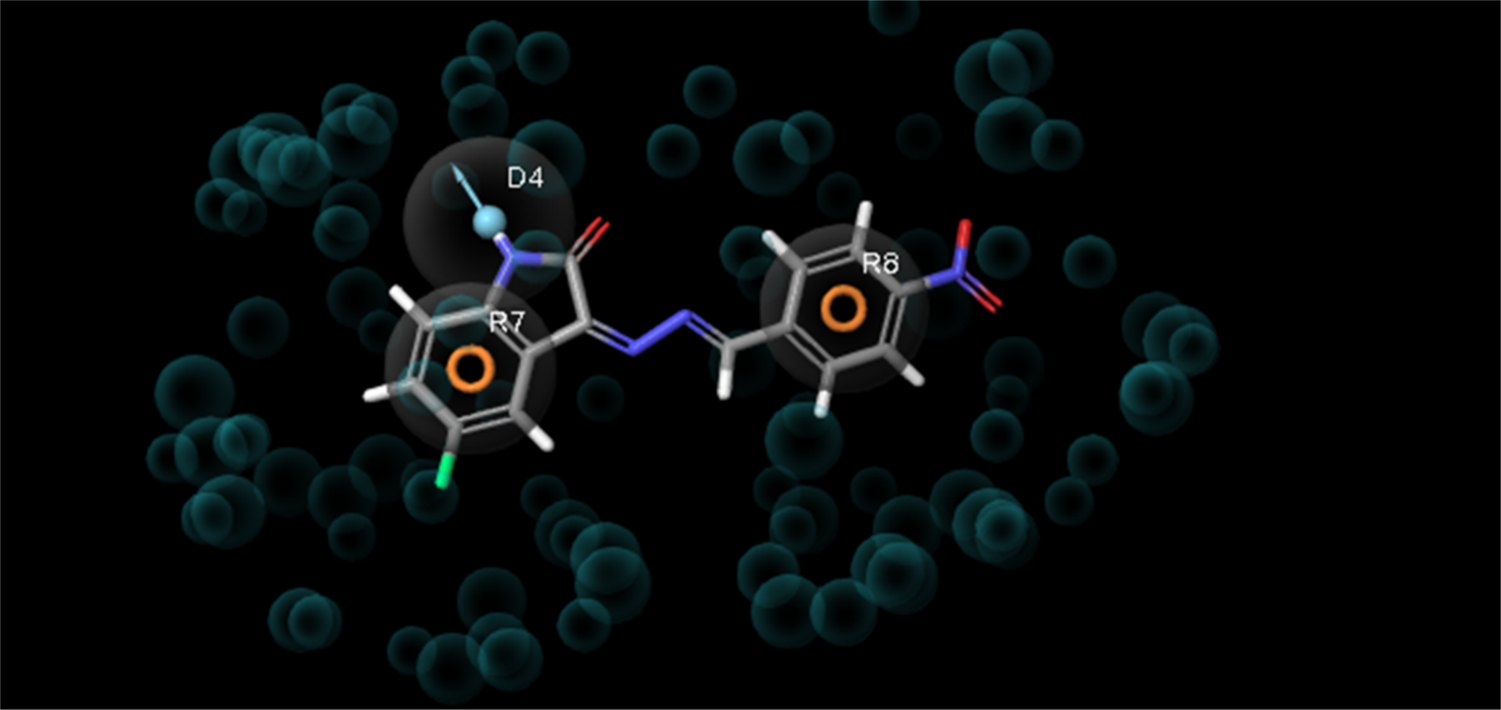
Generated pharmacophore hypothesis of EGFR TK complexed with aligned compound **8** as a reference.

The e-pharmacophore hypothesis formed by 3POZ, the crystal structure of the EGFR TK target, and compound **8** in the active site of the crystal structure, are presented in Figure 10. As shown in Figure 10, the e-pharmacophore hypothesis consists of three pharmacophores. These three pharmacophores interact with two aromatic rings (R7 and R8) and a hydrogen bond donor (D4).

**Figure 11:**
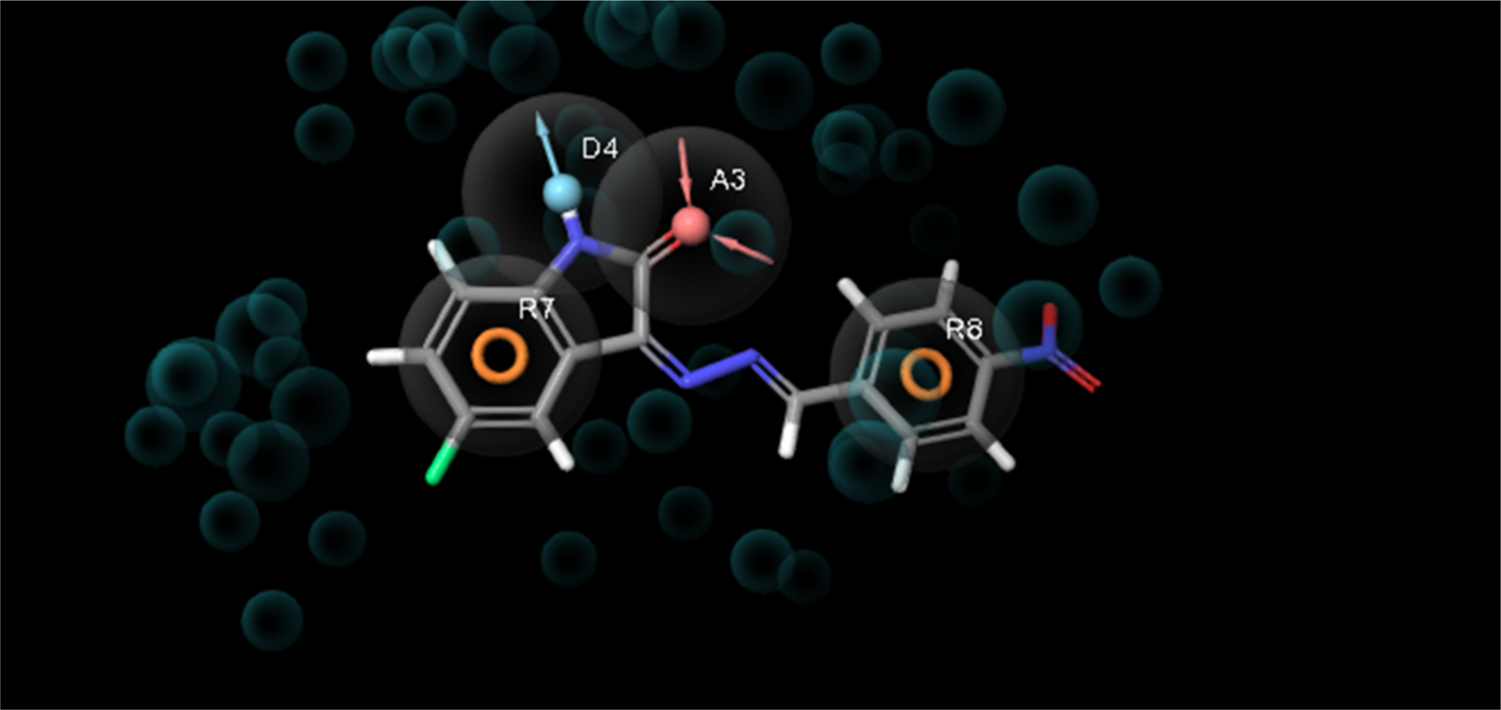
Generated pharmacophore hypothesis of EGFR complexed with aligned compound **8** as a reference.

The complex structure of the compound **8** ligand when it settles into the active site of the EGFR receptor, which is formed depending on the result of the Schrödinger 2021–2 Glide program, was examined. The e-pharmacophore hypothesis formed between the ligand and target in this complex is presented in Figure 11. It has been determined that the e-pharmacophore hypothesis of 4HJO and ligand compound **8** consists of four pharmacophores. These four pharmacophores involve two aromatic rings (R7 and R8), a hydrogen bond donor (D4), and a hydrogen bond acceptor (A3).

**Figure 12:**
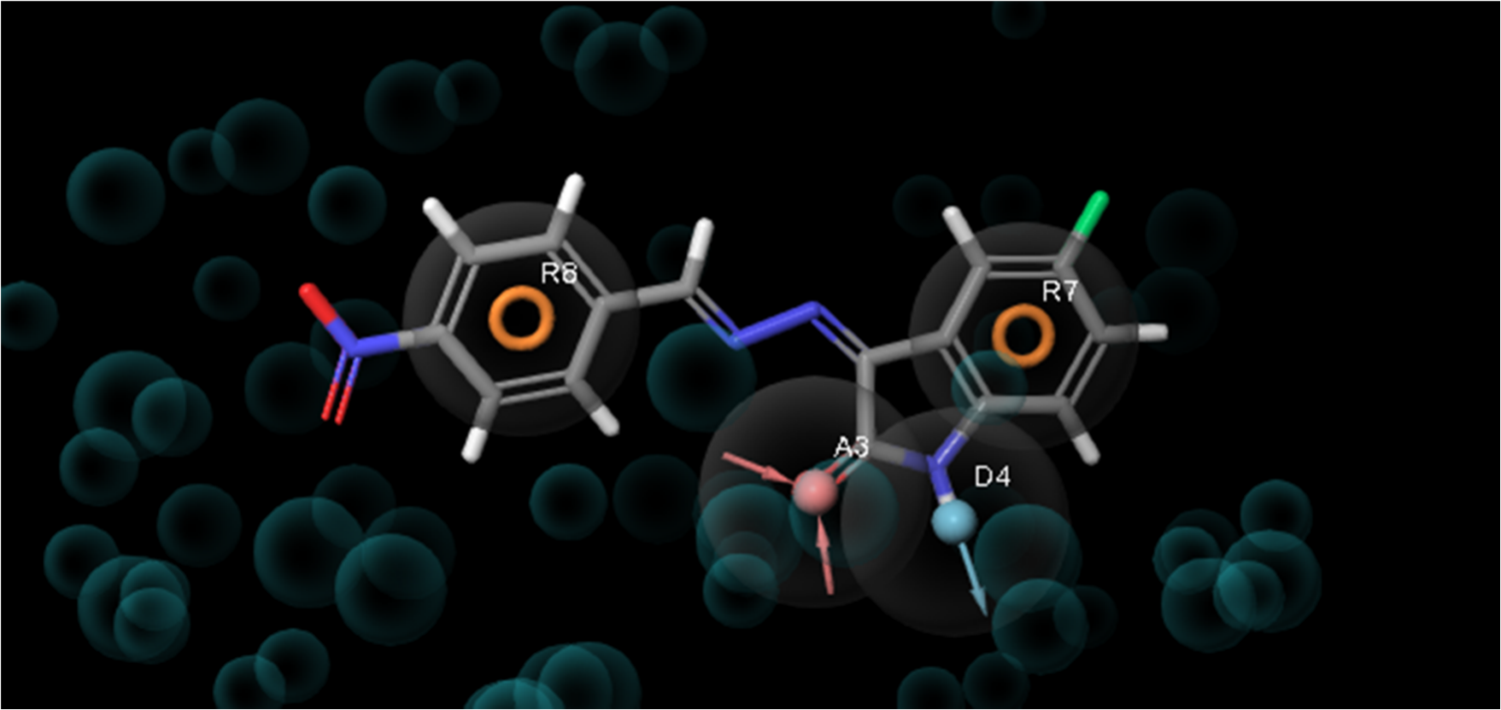
Generated pharmacophore hypothesis of PI3K α complexed with aligned compound **8** as reference.

The e-pharmacophore hypothesis analysis of the ligand–target complex after localization to the active site of compound **8**, which interacts with the PI3K α target *in silico*, is shown in Figure 12. As shown in Figure 12, the e-pharmacophore hypothesis of compound **8** with target 7TZ7 consists of four pharmacophores. These four pharmacophores involve two aromatic rings (R7 and R8), a hydrogen bond donor (D4), and a hydrogen bond acceptor (A3).

## 3 Conclusions

In this study, we reported the *in vitro* antiproliferative activity of fluorinated isatin-hydrazones using e-pharmacophore modeling, spectroscopic analysis, molecular docking, and ADME studies. Fifteen compounds synthesized within the scope of this study were tested *in vitro* in two cancer cell lines and one healthy human cell line. The results showed that compound **8**, which contains 4-nitrobenzylidene in its structure, had the highest cytotoxic effect among the tested compounds against both lung and liver cancer cell lines, with IC_50_ values of 42.43 and 48.43 μM, respectively. Computational studies were carried out to design the precursor compounds to support the experimental part of the study. The current study used *in silico* approaches combining e-pharmacophore modeling and structure-based molecular docking of targets to identify anticancer inhibitors. A good homology model was created for the targets (anticancer) identified in this study. An e-pharmacophore model was developed, and molecular docking and MM-GBSA binding energy calculations were performed after the synthesized compounds were used to verify their affinity for the target. The designed compounds were calculated based on the determined targets. The Glide gscore, dock score, and binding energy were further analyzed by using the ADME parameters. This study suggests that fluorinated isatin-hydrazone derivatives are good anticancer inhibitors. In addition, the drug similarity, pharmacokinetics, and physicochemical properties of the designed compounds were determined using SwissADME, which is an *in silico* approach. These compounds were interpreted to have the desired ADME properties. According to molecular docking based on the interactions of amino acid residues in the binding site, EGFR, VEGFR2, and PI3K targets were determined to have good stability at the binding site. Based on the results obtained from molecular docking, it can be considered that the *in vitro* data were supported. According to the binding parameter values, it can be said that the comparison compound may be more effective than cisplatin and may lead to other studies.

## 4 Experimental Section

### 4.1 Materials and Methods

All reagents and solvents required for the synthesis of anticancer agents were obtained from Merck and Sigma Aldrich. Melting points were ascertained using a capillary melting apparatus, namely, the DMP-100 melting point apparatus, and remained uncorrected. Elemental analyses were conducted using a Thermo Scientific Flash 2000 elemental analyzer. FT-IR spectra were obtained using a PerkinElmer 400 FT-IR/FT-FIR spectrometer spotlight 400 imaging system in the scan range 4000–400 cm^–1^. The 1D (^1^H-, ^13^C NMR, and APT) and 2D (HMBC and HETCOR) NMR spectra of the compounds dissolved in DMSO-*d*_6_ were obtained by using a Bruker Advance III 400 spectrometer.

#### 4.1.1 Synthesis of Compound **1**

A mixture of 5-fluoroisatin (1 mmol) and hydrazine monohydrate (1.5 equiv) in ethanol (20 mL) was refluxed for 1 h and cooled to room temperature. The precipitate was filtered, washed with water, and dried at room temperature in open air. The resulting product was recrystallized with ethanol.

##### 4.1.1.1 5-Fluoro-3-hydrazonoindolin-2-one (**1**)^49^

Yellow solid, yield: 0.136 g (76%), **m.p.:** 203–204 °C. **FT-IR/ATR (ν**_**max**_**) cm**^**–1**^**:** 3363, 3136 (N–H), 3034, 2966 (C–H), 1683 (C=O). ^**1**^**H NMR (DMSO-*****d***_**6**_, **400 MHz), ppm:** δ 10.72 (s, 1H, NH), 10.65 (d, *J* = 14.8 Hz, 1H, NH), 9.80 (d, *J* = 15.0 Hz, 1H, NH), 7.14 (d, *J* = 6.4 Hz, 1H, ArH), 6.99–6.44 (m, 1H, ArH), 6.86–6.82 (m, 1H, ArH). ^**13**^**C NMR (DMSO-*****d***_**6**_, **100 MHz), ppm:** δ 166.46, 163.44, 157.34, 135.16, 126.13, 124.12, 124.02, 113.70, 113.46, 111.29, 111.20, 110.52, 104.99, 104.73. **Elemental analysis for C**_**8**_**H**_**6**_**FN**_**3**_**O (179.15 g/mol), Calculated**: C, 53.63; H, 3.38; N, 23.46%. **Found**: C, 53.86; H, 3.23; N, 23.88%.

#### 4.1.2 General Procedure of Compounds **2**–**15**

A mixture of compound **1** (0.5 mmol) and substituted aromatic aldehyde (0.5 mmol) in absolute ethanol (15 mL) was added to 2–3 drops of glacial acetic acid. The reaction mixture was refluxed for 3 h. The reaction was completed followed by TLC. The resulting precipitate was filtered, washed with petroleum ether, and dried in open air. Then further, the target products were purified by crystallization with ethanol.

##### 4.1.2.1 5-Fluoro-3-{[benzylidene]hydrazinylidene}indolin-2-one (**2**)

Orange solid, yield: 0.095 g (71%), **m.p.:** 229–230 °C. **FT-IR/ATR (ν**_**max**_**) cm**^**–1**^**:** 3163 (N–H), 3090, 3043 (C–H), 1741 (C=O), 1625 (C=N). ^**1**^**H NMR (DMSO-*****d***_**6**_, **400 MHz), ppm:** δ 10.93 (s, 1H, NH), 8.67 (s, 1H, CH=N), 7.99 (d, *J* = 5.4 Hz, 2H, ArH), 7.66 (d, *J* = 6.4 Hz, 1H, ArH), 7.63–7.55 (m, 3H, ArH), 7.29 (t, *J* = 7.9 Hz, 1H, ArH), 6.91 (dd, *J* = 8.3, 4.1 Hz, 1H, ArH). ^**13**^**C NMR (DMSO-*****d***_**6**_, **100 MHz), ppm:** δ 164.95, 162.21, 159.09, 156.74, 150.87, 141.89, 133.65, 132.89, 129.77, 128.20, 120.78, 120.55, 117.25, 115.91, 115.65, 112.46. **Elemental analysis for C**_**15**_**H**_**10**_**FN**_**3**_**O (267.26 g/mol), Calculated**: C, 67.41; H, 3.77; N, 15.72%. **Found**: C, 67.71; H, 3.92; N, 15.54%.

##### 4.1.2.2 5-Fluoro-3-{[4-bromobenzylidene]hydrazinylidene}indolin-2-one (**3**)

Orange solid, yield: 0.113 g (65%), **m.p.:** 262–263 °C. **FT-IR/ATR (v**_**max**_**) cm**^**–1**^**:** 3133 (N–H), 3081, 2997 (C–H), 1730 (C=O), and 1625 (C=N). ^**1**^**H NMR (DMSO-*****d***_**6**_, **400 MHz), ppm:** δ 10.93 (s, 1H, NH), 8.67 (s, 1H, CH=N), 7.93 (d, *J* = 8.3 Hz, 2H, ArH), 7.81 (d, *J* = 8.3 Hz, 2H, ArH), 7.62 (dd, *J* = 8.1, 2.5 Hz, 1H, ArH), 7.35–7.25 (m, 1H, ArH), 6.91 (dd, *J* = 8.6, 4.2 Hz, 1H, ArH). ^**13**^**C NMR (DMSO-*****d***_**6**_, **100 MHz), ppm:** δ 164.88, 161.12, 159.11, 156.75, 150.83, 141.97, 132.84, 131.19, 126.53, 120.89, 120.66, 117.16, 117.07, 115.91, 115.66, 112.48, 112.40. **Elemental analysis for C**_**15**_**H**_**9**_**BrFN**_**3**_**O (346.16 g/mol), Calculated**: C, 52.05; H, 2.62; N, 12.14%. **Found**: C, 52.11; H, 2.85; N, 12.37%.

##### 4.1.2.3 5-Fluoro-3-{[4-chlorobenzylidene]hydrazinylidene}indolin-2-one (**4**)^50^

Orange solid, yield: 0.104 g (69%), **m.p.:** 257–258 °C. **FT-IR/ATR (ν**_**max**_**) cm**^**–1**^**:**3112 (N–H), 3065, 3008 (C–H), 1731 (C=O), 1618 (C=N). ^**1**^**H NMR (DMSO-*****d***_**6**_, **400 MHz), ppm:** δ 10.93 (s, 1H, NH), 8.69 (s, 1H, CH=N), 8.01 (d, *J* = 8.4 Hz, 2H, ArH), 7.67 (d, *J* = 8.4 Hz, 2H, ArH), 7.63 (dd, *J* = 8.2, 2.4 Hz, 1H), 7.35–7.25 (m, 1H, ArH), 6.91 (dd, *J* = 8.6, 4.2 Hz, 1H, ArH). ^**13**^**C NMR (DMSO-*****d***_**6**_, **100 MHz), ppm:** δ 164.88, 161.10, 159.08, 156.74, 150.94, 141.97, 137.49, 132.52, 131.07, 129.92, 120.89, 120.66, 117.17, 115.95, 115.69, 112.40. **Elemental analysis for C**_**15**_**H**_**9**_**ClFN**_**3**_**O (301.71 g/mol), Calculated**: C, 59.72; H, 3.01; N, 13.93%. **Found**: C, 59.89; H, 3.14; N, 13.65%.

##### 4.1.2.4 5-Fluoro-3-{[4-fluorobenzylidene]hydrazinylidene}indolin-2-one (**5**)^38^

Orange solid, yield: 0.091 g (64%), **m.p.:** 246–248 °C. **FT-IR/ATR (ν**_**max**_**) cm**^**–1**^**:** 3144 (N–H), 3076, 3003 (C–H), 1733 (C=O), 1624 (C=N). ^**1**^**H NMR (DMSO-*****d***_**6**_, **400 MHz), ppm:** δ 10.92 (s, 1H, NH), 8.71 (s, 1H, CH=N), 8.07 (dd, *J* = 8.5, 5.8 Hz, 2H, ArH), 7.68 (dd, *J* = 8.2, 2.5 Hz, 1H, ArH), 7.45 (t, *J* = 8.8 Hz, 2H, ArH), 7.34–7.24 (m, 1H, ArH), 6.91 (dd, *J* = 8.5, 4.3 Hz, 1H, ArH). ^**13**^**C NMR (DMSO-*****d***_**6**_, **100 MHz), ppm:** δ 166.22, 164.96, 163.72, 161.67, 159.11, 156.75, 151.11, 141.92, 132.08, 131.99, 130.35, 120.80, 120.56, 117.25, 117.15, 117.10, 116.88, 115.98, 115.72, 112.44, 112.36. **Elemental analysis for C**_**15**_**H**_**9**_**F**_**2**_**N**_**3**_**O (285.25 g/mol), Calculated**: C, 63.16; H, 3.18; N, 14.73%. **Found**: C, 63.35; H, 3.29; N, 14.41%.

##### 4.1.2.5 5-Fluoro-3-{[4-methylbenzylidene]hydrazinylidene}indolin-2-one (**6**)

Orange solid, yield: 0.096 g (68%), **m.p.:** 241–242 °C. **FT-IR/ATR (ν**_**max**_**) cm**^**–1**^**:** 3154 (N–H), 3076, 3003 (C–H), 1760 (C=O), 1624 (C=N). ^**1**^**H NMR (DMSO-*****d***_**6**_, **400 MHz), ppm:** δ 10.90 (s, 1H, NH), 8.66 (s, 1H, CH=N), 7.88 (d, *J* = 7.8 Hz, 2H, ArH), 7.70 (d, *J* = 8.2 Hz, 1H, ArH), 7.40 (d, *J* = 7.8 Hz, 2H, ArH), 7.28 (t, *J* = 8.9 Hz, 1H, ArH), 6.91 (dd, *J* = 8.5, 4.2 Hz, 1H, ArH), 2.40 (s, 3H, CH_3_). ^**13**^**C NMR (DMSO-*****d***_**6**_, **100 MHz), ppm:** δ 165.04, 162.78, 159.09, 156.74, 150.89, 143.36, 141.79, 131.04, 130.39, 129.53, 120.65, 120.41, 117.34, 117.25, 115.90, 115.64, 112.39, 112.32, 21.76. **Elemental analysis for C**_**16**_**H**_**12**_**FN**_**3**_**O (281.29 g/mol), Calculated**: C, 68.32; H, 4.30; N, 14.94%. **Found**: C, 68.51; H, 4.47; N, 14.74%.

##### 4.1.2.6 5-Fluoro-3-{[4-methoxybenzylidene]hydrazinylidene}indolin-2-one (**7**)

Orange solid, yield: 0.094 g (63%), **m.p.:** 247–248 °C. **FT-IR/ATR (ν**_**max**_**) cm**^**–1**^**:** 3159 (N–H), 3101, 3008 (C–H), 1742 (C=O), 1620 (C=N). ^**1**^**H NMR (DMSO-*****d***_**6**_, **400 MHz), ppm:** δ 10.87 (s, 1H, NH), 8.70 (s, 1H, CH=N), 7.97 (d, *J* = 8.6 Hz, 2H, ArH), 7.80 (dd, *J* = 8.2, 2.5 Hz, 1H, ArH), 7.33–7.23 (m, 1H, ArH), 7.16 (d, *J* = 8.6 Hz, 2H, ArH), 6.90 (dd, *J* = 8.5, 4.2 Hz, 1H, ArH), 3.87 (s, 3H, OCH_3_). ^**13**^**C NMR (DMSO-*****d***_**6**_, **100 MHz), ppm:** δ 165.25, 164.07, 163.38, 159.11, 156.76, 151.07, 141.64, 131.73, 126.28, 120.48, 120.24, 117.49, 117.40, 116.00, 115.70, 115.36, 112.32, 56.04. **Elemental analysis for C**_**16**_**H**_**12**_**FN**_**3**_**O**_**2**_**(297.29 g/mol), Calculated**: C, 64.64; H, 4.07; N, 14.13%. **Found**: C, 64.30; H, 4.21; N, 14.35%.

##### 4.1.2.7 **5**-Fluoro-3-{[4-nitrobenzylidene]hydrazinylidene}indolin-2-one (**8**)

Red solid, yield: 0.101 g (65%), **m.p.:** 249–251 °C. **FT-IR/ATR (ν**_**max**_**) cm**^**–1**^**:** 3243 (N–H), 3081, 2987 (C–H), 1734 (C=O), 1616 (C=N). ^**1**^**H NMR (DMSO-*****d***_**6**_, **400 MHz), ppm:** δ 10.96 (s, 1H, NH), 8.73 (s, 1H, CH=N), 8.40 (d, *J* = 8.7 Hz, 2H, ArH), 8.22 (d, *J* = 8.7 Hz, 2H, ArH), 7.49 (dd, *J* = 8.1, 2.5 Hz, 1H, ArH), 7.32–7.27 (m, 1H, ArH), 6.94–6.90 (m, 1H, ArH). ^**13**^**C NMR (DMSO-*****d***_**6**_, **100 MHz), ppm:** δ 164.65, 159.12, 158.41, 156.75, 150.35, 149.63, 142.16, 139.31, 133.09, 130.90, 130.31, 124.79, 124.49, 121.19, 120.96, 116.81, 115.87, 115.62, 112.62. **Elemental analysis for C**_**15**_**H**_**9**_**FN**_**4**_**O**_**3**_**(312.26 g/mol), Calculated**: C, 57.70; H, 2.91; N, 17.94%. **Found**: C, 57.49; H, 3.06; N, 17.56%.

##### 4.1.2.8 5-Fluoro-3-{[2-hydroxybenzylidene]hydrazinylidene}indolin-2-one (**9**)

Red solid, yield: 0.088 g (62%), **m.p.:** 252–253 °C. **FT-IR/ATR (ν**_**max**_**) cm**^**–1**^**:** 3216 (O–H), 3199 (N–H), 3107, 3029 (C–H), 1733 (C=O), 1615 (C=N). ^**1**^**H NMR (DMSO-*****d***_**6**_, **400 MHz), ppm:** δ 10.89 (s, 1H, NH), 10.67 (s, 1H, OH), 8.92 (s, 1H, CH=N), 7.94 (d, *J* = 7.9 Hz, 1H, ArH), 7.78 (dd, *J* = 8.3, 2.3 Hz, 1H, ArH), 7.43 (d, *J* = 7.6 Hz, 1H, ArH), 7.32–7.24 (m, 1H, ArH), 7.01 (dd, *J* = 7.5, 4.3 Hz, 2H, ArH), 6.90 (dd, *J* = 8.5, 4.3 Hz, 1H, ArH). ^**13**^**C NMR (DMSO-*****d***_**6**_, **100 MHz), ppm:** δ 165.15, 161.32, 160.40, 159.23, 156.76, 151.19, 141.81, 134.78, 129.09, 120.65, 120.37, 119.67, 117.39, 117.27, 115.69, 115.43, 112.36, 112.28. **Elemental analysis for C**_**15**_**H**_**10**_**FN**_**3**_**O**_**2**_**(283.26 g/mol), Calculated**: C, 63.60; H, 3.56; N, 14.83%. **Found**: C, 63.82; H, 3.32; N, 14.99%.

##### 4.1.2.9 5-Fluoro-3-{[3-hydroxybenzylidene]hydrazinylidene}indolin-2-one (**10**)

Orange solid, yield: 0.096 g (68%), **m.p.:** 260–261 °C. **FT-IR/ATR (ν**_**max**_**) cm**^**–1**^**:** 3394 (O–H), 3217 (N–H), 3081, 3027 (C–H), 1731 (C=O), 1625 (C=N). ^**1**^**H NMR (DMSO-*****d***_**6**_, **400 MHz), ppm:** δ 10.92 (s, 1H, NH), 9.91 (s, 1H, OH), 8.60 (s, 1H, CH=N), 7.66 (dd, *J* = 8.3, 2.5 Hz, 1H, ArH), 7.43–7.36 (m, 3H, ArH), 7.35–7.26 (m, 1H, ArH), 7.04–6.98 (m, 1H, ArH), 6.92 (dd, *J* = 8.6, 4.2 Hz, 1H, ArH). ^**13**^**C NMR (DMSO-*****d***_**6**_, **100 MHz), ppm:** δ 165.00, 162.33, 159.11, 158.33, 156.75, 150.79, 141.84, 134.89, 130.88, 121.23, 120.78, 120.54, 120.28, 117.27, 117.17, 115.81, 115.55, 114.68, 112.50, 112.43. **Elemental analysis for C**_**15**_**H**_**10**_**FN**_**3**_**O**_**2**_**(283.26 g/mol), Calculated**: C, 63.60; H, 3.56; N, 14.83%. **Found**: C, 63.48; H, 3.67; N, 14.61%.

##### 4.1.2.10 5-Fluoro-3-{[4-hydroxybenzylidene]hydrazinylidene}indolin-2-one (**11**)

Orange solid, yield: 0.099 g (70%), **m.p.:** 275–276 °C. **FT-IR/ATR (ν**_**max**_**) cm**^**–1**^**:** 3279 (O–H), 3180 (N–H), 3117, 3050 (C–H), 1722 (C=O), 1610 (C=N). ^**1**^**H NMR (DMSO-*****d***_**6**_, **400 MHz), ppm:** δ 10.86 (s, 1H, NH), 10.48 (s, 1H, OH), 8.67 (s, 1H, CH=N), 7.87 (d, *J* = 8.2 Hz, 2H, ArH), 7.86–7.82 (m, 1H, ArH), 7.27 (t, *J* = 8.0 Hz, 1H, ArH), 6.97 (d, *J* = 8.1 Hz, 2H, ArH), 6.90 (dd, *J* = 8.5, 4.1 Hz, 1H, ArH). ^**13**^**C NMR (DMSO-*****d***_**6**_, **100 MHz), ppm:** δ 165.19, 162.43, 159.10, 156.76, 150.96, 141.55, 132.14, 124.78, 120.30, 120.07, 117.61, 117.51, 116.76, 115.91, 115.71, 112.14. **Elemental analysis for C**_**15**_**H**_**10**_**FN**_**3**_**O**_**2**_**(283.26 g/mol), Calculated**: C, 63.60; H, 3.56; N, 14.83%. **Found**: C, 63.88; H, 3.44; N, 14.77%.

##### 4.1.2.11 5-Fluoro-3-{[2-hydroxy-3-methoxybenzylidene]hydrazinylidene}indolin-2-one (**12**)

Shiny dark brown solid, yield: 0.096 g (61%), **m.p.:** 244–246 °C. **FT-IR/ATR (ν**_**max**_**) cm**^**–1**^**:** 3235 (O–H), 3217 (N–H), 3080, 3009 (C–H), 1722 (C=O), 1624 (C=N). ^**1**^**H NMR (DMSO-*****d***_**6**_, **400 MHz), ppm:** δ 11.87 (s, 1H, OH), 11.03 (s, 1H, NH), 9.01 (s, 1H, CH=N), 7.43 (dd, *J* = 7.7, 2.4 Hz, 1H, ArH), 7.33–7.29 (m, 1H, ArH), 7.27 (s, 1H, ArH), 7.18 (d, *J* = 7.9 Hz, 1H, ArH), 6.96–6.89 (m, 2H, ArH), 3.82 (s, 3H, OCH_3_). ^**13**^**C NMR (DMSO-*****d***_**6**_, **100 MHz), ppm:** δ 167.51, 165.15, 160.99, 159.80, 159.10, 156.75, 150.52, 149.00, 148.77, 141.79, 124.89, 120.72, 120.48, 120.21, 119.99, 119.85, 119.55, 117.29, 117.21, 116.03, 115.54, 115.26, 112.44, 112.37, 56.46. **Elemental analysis for C**_**16**_**H**_**12**_**FN**_**3**_**O**_**3**_**(313.29 g/mol), Calculated**: C, 61.34; H, 3.86; N, 13.41%. **Found**: C, 61.56; H, 3.72; N, 13.80%.

##### 4.1.2.12 5-Fluoro-3-{[2-hydroxy-4-methoxybenzylidene]hydrazinylidene}indolin-2-one (**13**)

Orange solid, yield: 0.089 g (57%), **m.p.:** 257–258 °C. **FT-IR/ATR (ν**_**max**_**) cm**^**–1**^**:** 3201 (O–H), 3128 (N–H), 3044, 2987 (C–H), 1734 (C=O), 1614 (C=N). ^**1**^**H NMR (DMSO-*****d***_**6**_, **400 MHz), ppm:** δ 12.29 (s, 1H, OH), 10.95 (s, 1H, NH), 8.98 (s, 1H, CH=N), 7.85 (t, *J* = 7.6 Hz, 1H, ArH), 7.58 (d, *J* = 8.7 Hz, 1H, ArH), 7.39 (d, *J* = 5.6 Hz, 1H, ArH), 7.28 (d, *J* = 9.2 Hz, 1H, ArH), 6.89 (d, *J* = 4.1 Hz, 1H, ArH), 6.55 (d, *J* = 3.3 Hz, 1H, ArH), 3.83 (s, 3H, OCH_3_). ^**13**^**C NMR (DMSO-*****d***_**6**_, **100 MHz), ppm:** δ 168.31, 165.21, 163.83, 161.57, 159.81, 157.28, 150.75, 149.89, 141.51, 135.49, 131.50, 120.30, 120.06, 117.40, 115.29, 115.08, 112.83, 112.17, 108.24, 101.42, 55.97. **Elemental analysis for C**_**16**_**H**_**12**_**FN**_**3**_**O**_**3**_**(313.29 g/mol), Calculated**: C, 61.34; H, 3.86; N, 13.41%. **Found**: C, 61.49; H, 3.61; N, 13.69%.

##### 4.1.2.13 5-Fluoro-3-{[3-hydroxy-4-methoxybenzylidene]hydrazinylidene}indolin-2-one (**14**)

Orange solid, yield: 0.108 g (62%), **m.p.:** 263–264 °C. **FT-IR/ATR (ν**_**max**_**) cm**^**–1**^**:** 3361 (O–H), 3206 (N–H), 3076, 3002 (C–H), 1721 (C=O), 1620 (C=N). ^**1**^**H NMR (DMSO-*****d***_**6**_, **400 MHz), ppm:** δ 10.87 (s, 1H, NH), 9.64 (s, 1H, OH), 8.62 (s, 1H, CH=N), 7.81 (dd, *J* = 8.3, 2.4 Hz, 1H, ArH), 7.51 (s, 1H, ArH), 7.39 (d, *J* = 8.3 Hz, 1H, ArH), 7.31–7.26 (m, 1H, ArH), 7.12 (d, *J* = 8.3 Hz, 1H, ArH), 6.91 (dd, *J* = 8.5, 4.2 Hz, 1H, ArH), 3.87 (s, 3H, OCH_3_). ^**13**^**C NMR (DMSO-*****d***_**6**_, **100 MHz), ppm:** δ 165.32, 164.60, 159.13, 156.77, 152.60, 147.53, 141.57, 126.45, 124.35, 120.44, 120.20, 117.36, 115.86, 115.60, 113.93, 112.51, 112.27, 56.19. **Elemental analysis for C**_**16**_**H**_**12**_**FN**_**3**_**O**_**3**_**(313.29 g/mol), Calculated**: C, 61.34; H, 3.86; N, 13.41%. **Found**: C, 61.58; H, 3.99; N, 13.83%.

##### 4.1.2.14 5-Fluoro-3-{[4-hydroxy-3-methoxybenzylidene]hydrazinylidene}indolin-2-one (**15**)

Red solid, yield: 0.102 g (65%), **m.p.:** 232–233 °C. **FT-IR/ATR (ν**_**max**_**) cm**^**–1**^**:** 3248 (O–H), 3175(N–H), 3102, 3013 (C–H), 1726 (C=O), 1620 (C=N). ^**1**^**H NMR (DMSO-*****d***_**6**_, **400 MHz), ppm:** δ 10.86 (s, 1H, NH), 10.14 (s, 1H, OH), 8.64 (s, 1H, CH=N), 7.88–7.81 (m, 1H, ArH), 7.56 (s, 1H, ArH), 7.48 (d, *J* = 8.2 Hz, 1H, ArH), 7.31–7.22 (m, 1H, ArH), 6.97 (d, *J* = 8.1 Hz, 1H, ArH), 6.89 (dd, *J* = 8.5, 4.3 Hz, 1H, ArH), 3.88 (s, 3H, OCH_3_). ^**13**^**C NMR (DMSO-*****d***_**6**_, **100 MHz), ppm:** δ 165.32, 164.74, 159.09, 156.73, 152.06, 150.98, 148.61, 141.53, 125.04, 120.26, 120.02, 117.61, 117.52, 116.48, 115.99, 115.74, 112.14, 55.98. **Elemental analysis for C**_**16**_**H**_**12**_**FN**_**3**_**O**_**3**_**(313.29 g/mol), Calculated**: C, 61.34; H, 3.86; N, 13.41%. **Found**: C, 61.44; H, 3.67; N, 13.63%.

### 4.2 Cytotoxic Activity Studies

This section of the study was performed following the procedure described in our previous study.^51,52^ The human lung cancer cell line (A549; ATCC CCL-185), human liver cancer cell line (HepG2; ATCC HB-8065), and human normal embryonic kidney cell line (HEK-293T) were cultured in high-glucose Dulbecco’s modified Eagle’s medium supplemented with 10% fetal bovine serum and 1% glutamax. Cells were seeded at a density of 5 × 10^3^ cells/well in sterile 96-well plates and incubated for 24 h. Following incubation, the cells were exposed to the prepared compounds (**1**–**15**) and cisplatin at 200, 100, 50, and 25 μM concentrations for 72 h. After the incubation period, the medium in the plates was carefully removed and the prepared MTT solution was added to each well of the plates. The plates were incubated for 2 h in an incubator. Absorbance was measured using an Epoch 2 ELISA plate reader at 590 nm. The IC_50_ values were calculated using GraphPad Prism Software version 5.

### 4.3 Computational Studies

#### 4.3.1 Predicting Drug-Likeness and Physicochemical Properties with SwissADME

In computational studies, the estimation of absorption, distribution, metabolism, excretion, and toxicity (ADMET) values is used as a tool for designing lead ligands. An important feature of the designed ligand is its “drug-likeness”. In this study, the data specified for this purpose were estimated using the SwissADME (http://www.swissadme.ch/) server. The pharmacokinetic properties of all designed and synthesized compounds, including molecular weight (MW), partition coefficient (log *P*), number of hydrogen bond donors (HBD), number of hydrogen bond acceptors (HBA), and topological polar surface area (PTSA) were calculated. In addition, to determine drug-likeness, whether the lead compounds complied with the Lipinski, Egan, Veber, Ghose, Egan, Muegge, and bioavailability score rules was considered when evaluating them as drug candidates.

In addition, log *K*_p_, GI absorption, BBB permeability, P-gp substrate, and cytochrome-P enzyme inhibition values were calculated using SiwssADME to estimate the absorption and distribution of the compounds.

### 4.4 Ligand Docking Studies

#### 4.4.1 Preparing the Ligands

The designed and synthesized compounds were prepared using the LigPrep wizard of the Schrödinger 2021–2 program.^53^ This was carried out using compounds **1**–**15** and the cisplatin ligand preparation procedure.

#### 4.4.2 Determination and Preparation of the Targets

Targets that are important in cancer signaling pathways were identified in this study. Since the human lung cancer cell line, human liver cancer cell line, and human normal embryonic kidney cell line were examined *in vitro*, the targets were selected accordingly. In this study, EGFR was preferred for lung cancer, VEGFR2 was preferred for liver cancer, and PI3K and EGFR kinase were preferred for the embryonic kidney cell line. In addition, because these targets prevent cancerous cells from spreading and metastasis throughout the body, *in silico* interactions of the designed compounds are important.

The next step after the preparation of the designed compounds was the preparation of proteins. The protein preparation procedure is applied for each target with which the ligands interact and is obtained from the protein database.^54^ In this study, four different targets and ligands interacted *in silico* with the molecular docking method to support the experimental data crystal structures: 4HJO (cocrystallized ligand: erlotinib)^55^ for EGFR, 3POZ (cocrystallized ligand: tak-285)^56^ for EGFR TK, 4ASD (cocrystallized ligand: sorafenib)^57^ for VEGFR2, and 7TZ7 (cocrystallized ligand: an inhibitor)^58^ for PI3K α. The targets were prepared using the ProteinPrep wizard of the Schrödinger 2021–2 Glide program.^53^

#### 4.4.3 Receptor Grid Generation

The grid boxes where the ligand was placed on the target were created using the Maestro Schrödinger Glide “Receptor Grid Generation” module. The area of the active site to be bound was used to determine the centers of bound ligands on the target with the receptor grid generation wizard. Grid boxes were identified for each target, and compound **8** was docked.

#### 4.4.4 Molecular Docking and Binding Free Energy Calculations

Compound **8** interacted with the crystal structures 4HJO, 4ASD, 3POZ, and 7TZ7 one by one, respectively. Binding parameter values were calculated as a result of the interaction with the ligand docking procedure. The accuracy of the numerical data was verified via redocking. The ligand inside the crystal structure was removed and recomplexed. The docking score was calculated and validated using molecular docking.

#### 4.4.5 Determination of e-Pharmacophore

The pharmacophore design of a ligand is based on the structural properties and conformation of the interacting target. Using this method, we determined the groups in the pharmacophore model of the ligand located in the active site of the target protein. The e-pharmacophore model was analyzed using the phase module of the modeling process of the Schrödinger 2021–2 program.^53^ A set of six standardized chemical properties, including hydrogen bond acceptor (A), hydrogen bond donor (D), positively ionizable (P), negatively ionizable (N), hydrophobic (H), and aromatic ring (R) were used to build the pharmacophore model.

## Supporting Information Available

FT-IR, ^1^H, and ^13^C NMR spectra of compounds (**1**–**15**) are available in the Supporting Information file (PDF)
