# Supplementary material for: Investigation of Newly Synthesized Fluorinated Isatin-Hydrazones by In Vitro Antiproliferative Activity, Molecular Docking, ADME Analysis, and e-Pharmacophore Modeling
Source: ACS Omega. 2024 Jun 5;9(24):26503–18. doi: 10.1021/acsomega.4c03014 (PMC11191101; doi:10.1021/acsomega.4c03014)

Supporting Information

Investigation of newly synthesized fluorinated isatin-hydrazones by *in vitro* antiproliferative activity, molecular docking, ADME analysis and e-pharmacophore modelling

Eyüp Başaran[a,*], Semiha Köprü[b,c,*], Senem Akkoç[d,e], Burçin Türkmenoğlu[f]

[a]*Department of Chemistry and Chemical Processing Technologies, Vocational School of Technical Sciences, Batman University, Batman 72060, Türkiye.*

[b]*Department of Chemistry, Faculty of Sciences, Erciyes University, Kayseri 38039, Türkiye.*

[c]*Technology Research and Application Center, Erciyes University, Kayseri 38039, Türkiye.*

[d]*Department of Basic Pharmaceutical Sciences, Faculty of Pharmacy, Suleyman Demirel University, Isparta 32260, Türkiye.*

[e]*Faculty of Engineering and Natural Sciences, Bahcesehir University, Istanbul 34353, Türkiye.*

[f]*Department of Analytical Chemistry, Faculty of Pharmacy, Erzincan Binali Yildirim University, Erzincan 24002, Türkiye.*

[*] Corresponing authors:
Eyüp Başaran
Department of Chemistry and Chemical Processing Technologies, Vocational School of Technical Sciences, Batman University, Türkiye.
Semiha Köprü
Department of Chemistry, Faculty of Sciences, Erciyes University, Kayseri, Türkiye
E-mail address: eyup.basaran@batman.edu.tr (E.Basaran) & semihaaydin@erciyes.edu.tr (S.Köprü)

FT-IR and NMR spectra of compounds 1-15

FT-IR spectrum of compound **1**

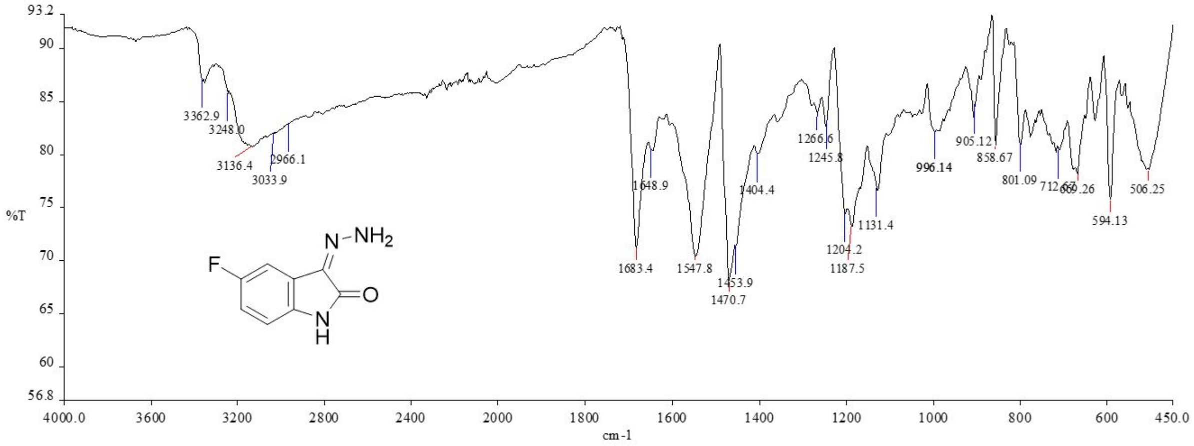

^{1}H NMR spectrum of compound **1**

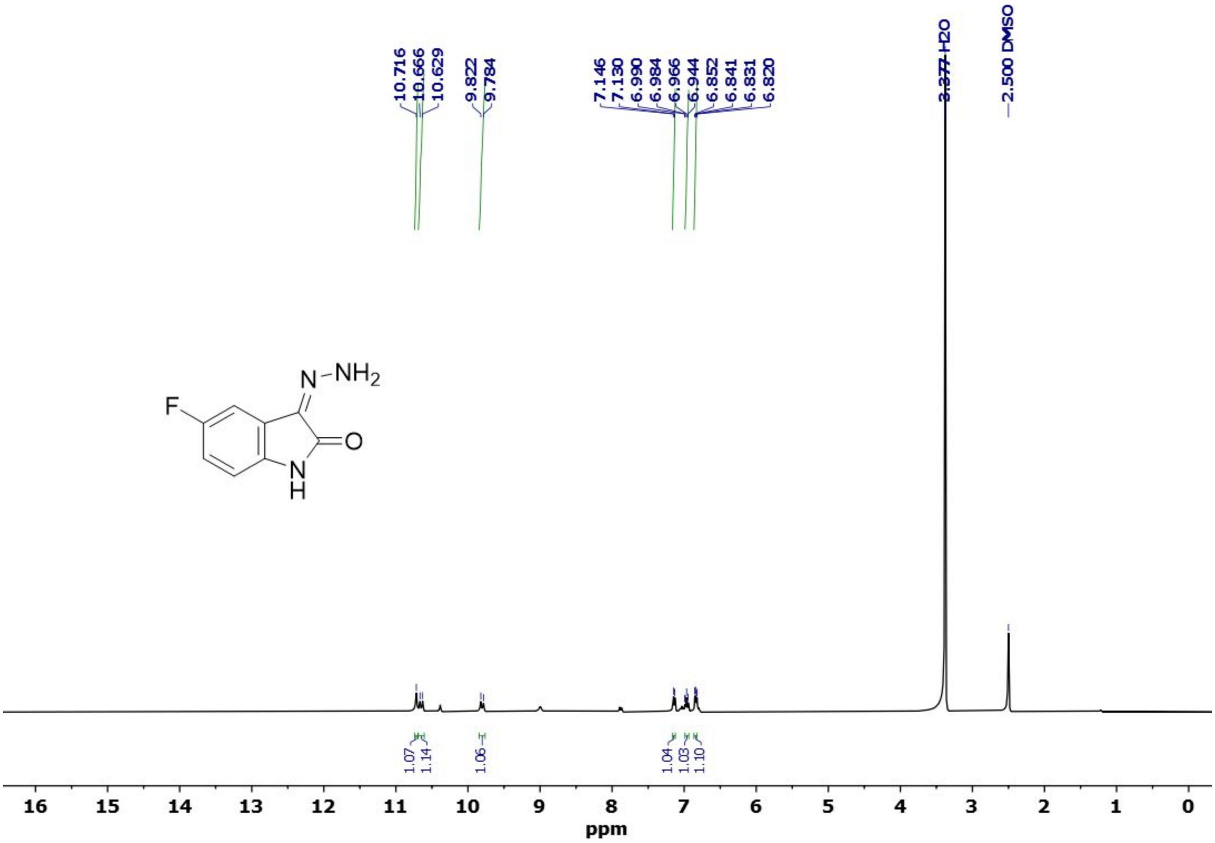

^{13}C NMR spectrum of compound **1**

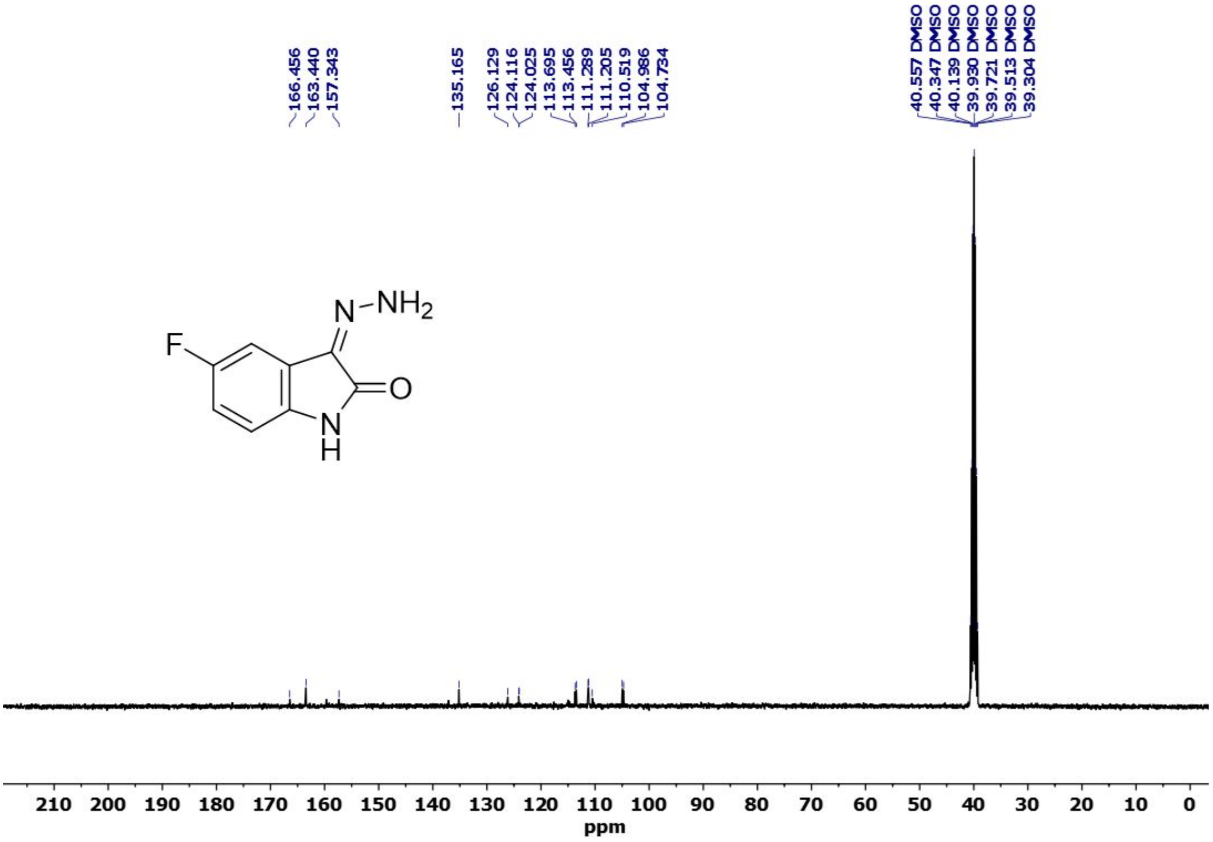

FT-IR spectrum of compound **2**

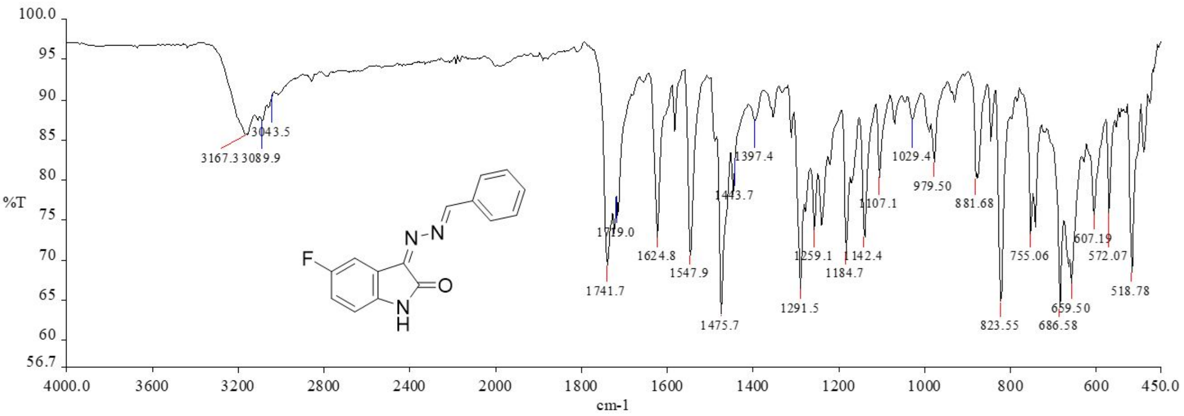

^{1}H NMR spectrum of compound **2**

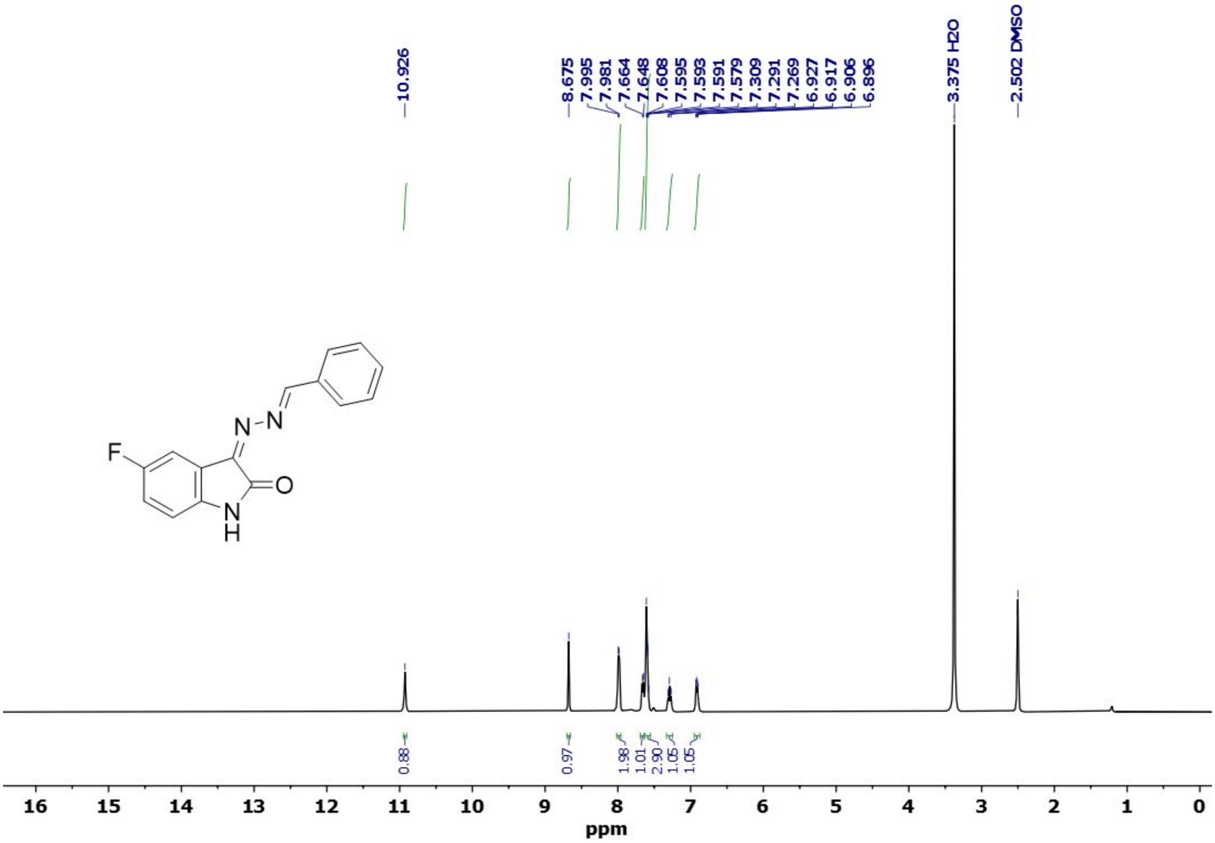

^{13}C NMR spectrum of compound **2**

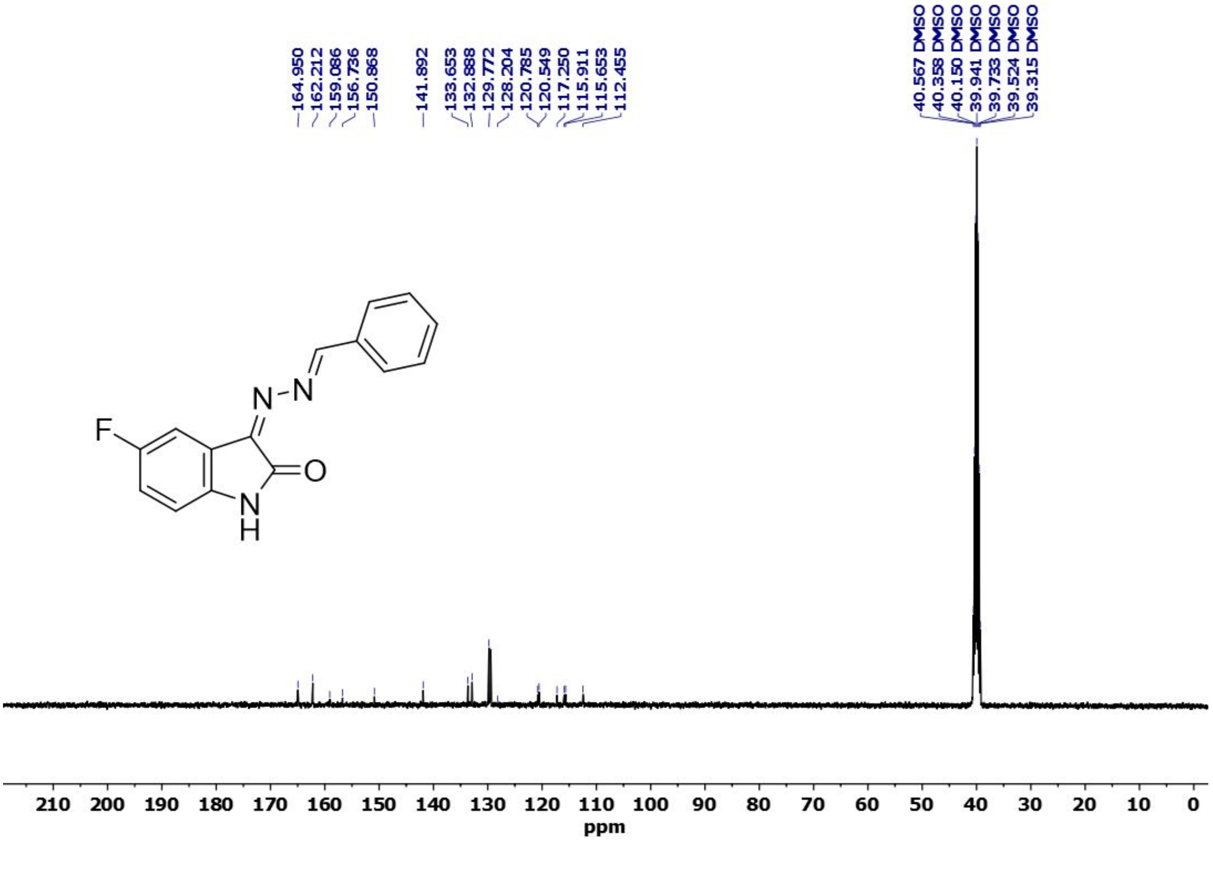

FT-IR spectrum of compound **3**

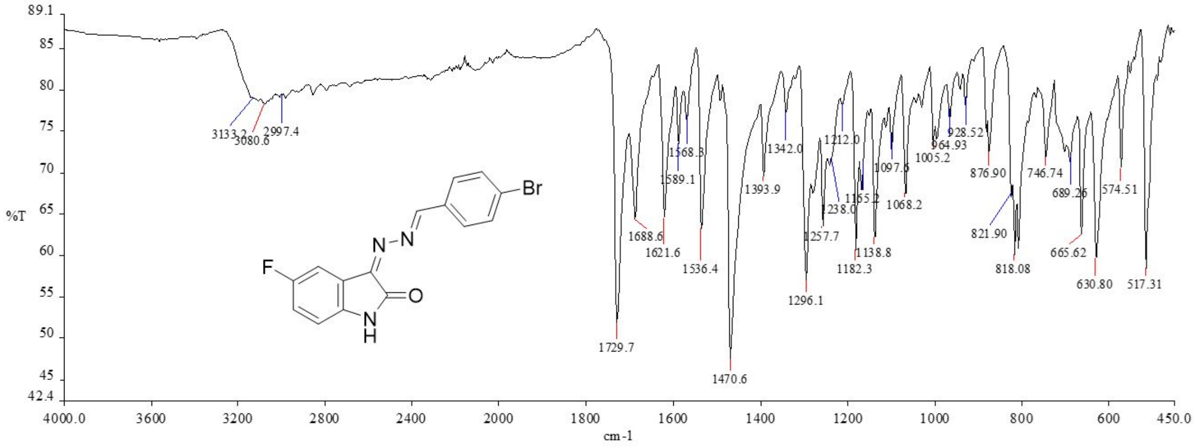

^{1}H NMR spectrum of compound **3**

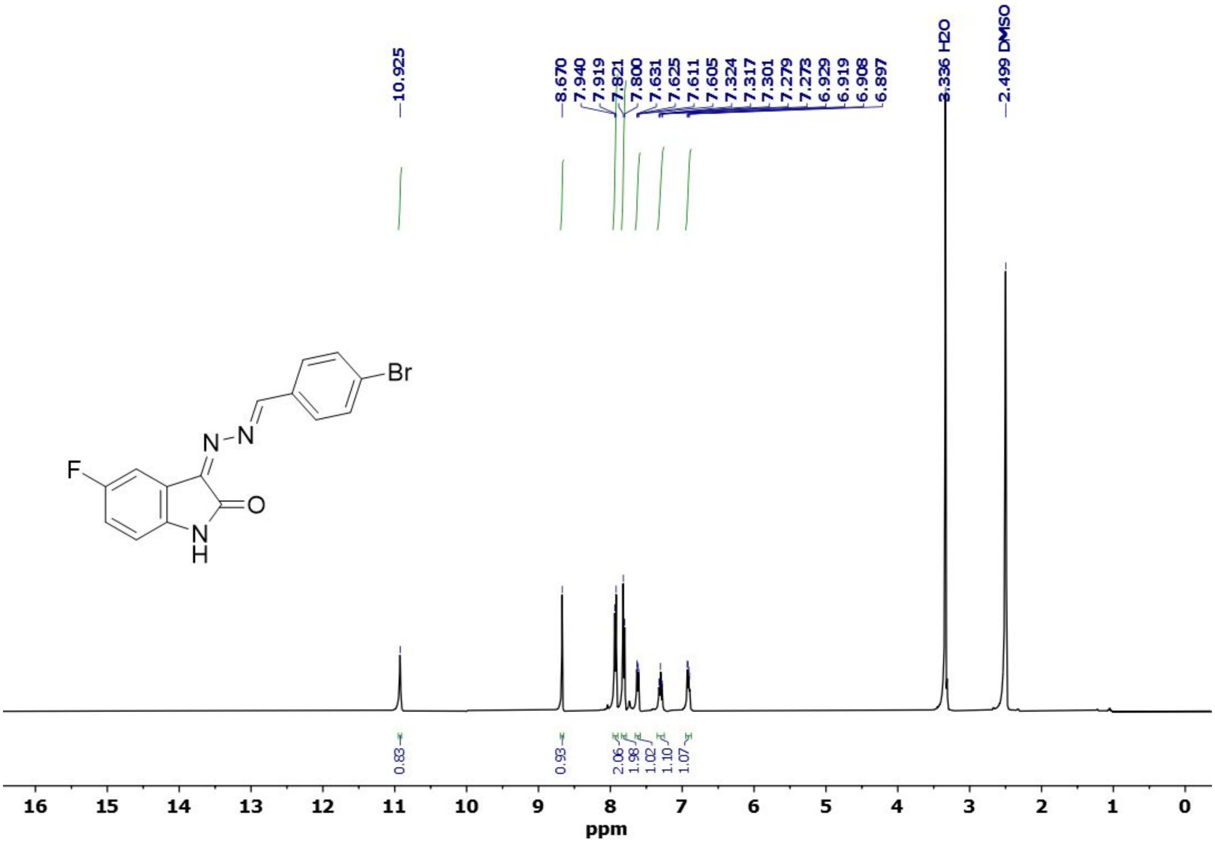

^{13}C NMR spectrum of compound **3**

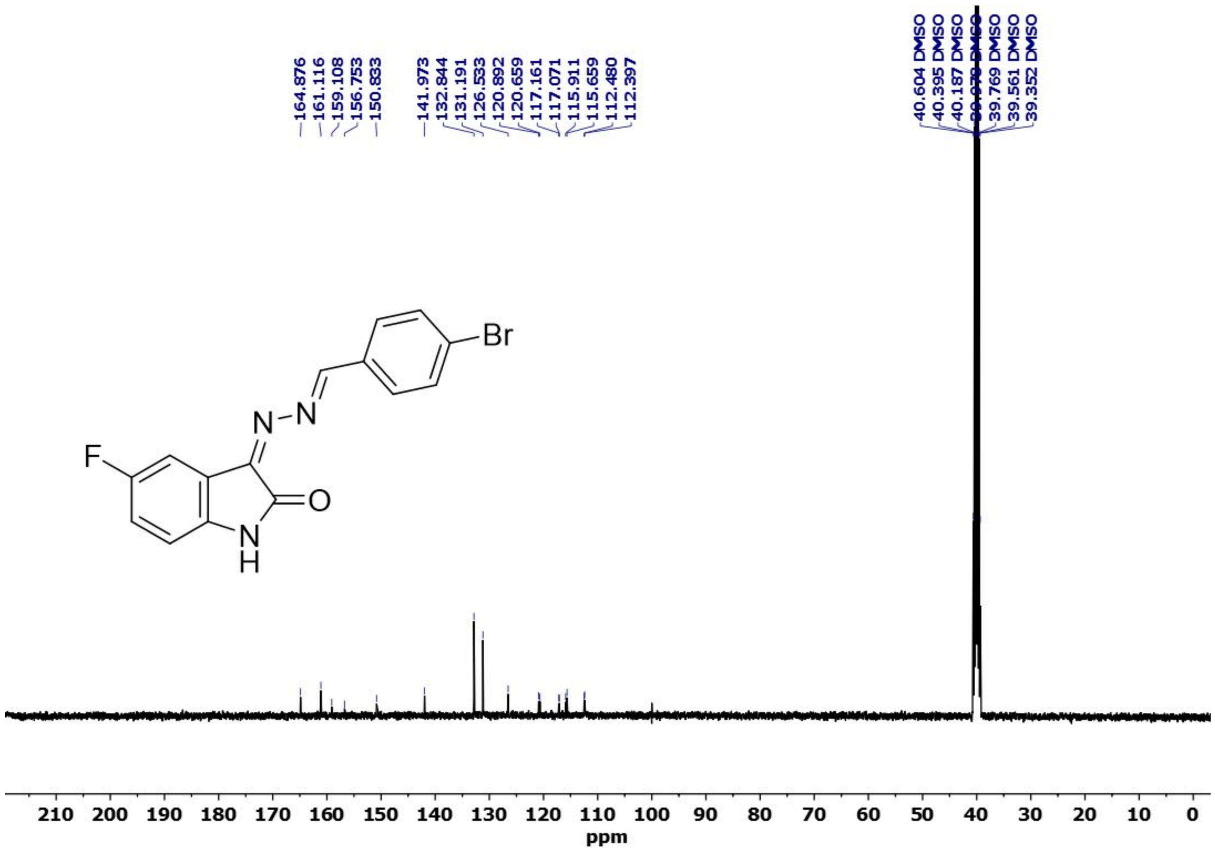

FT-IR spectrum of compound **4**

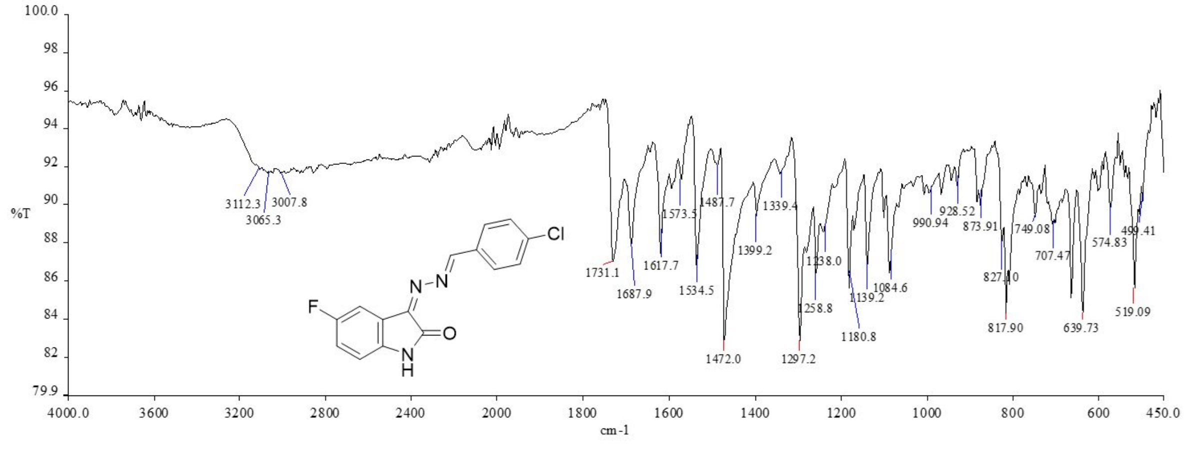

^{1}H NMR spectrum of compound **4**

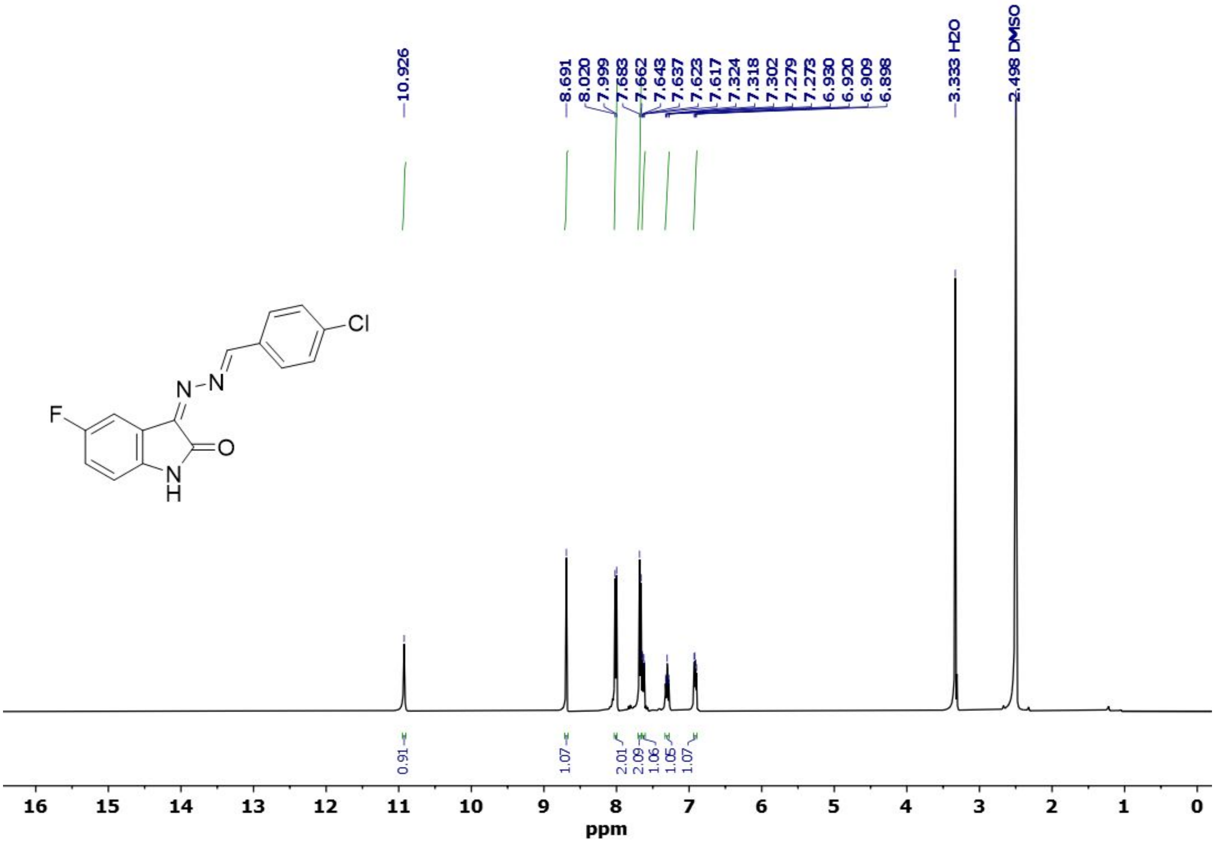

^{13}C NMR spectrum of compound **4**

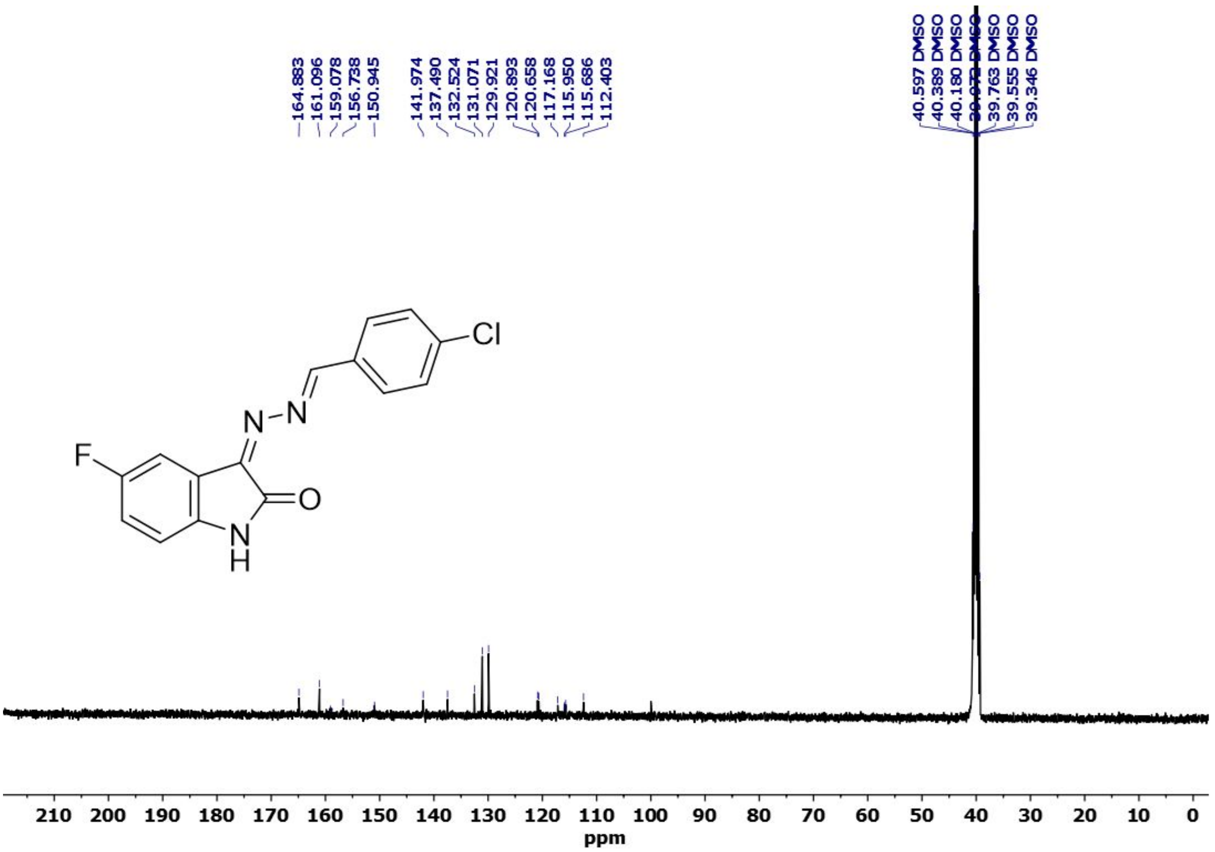

FT-IR spectrum of compound **5**

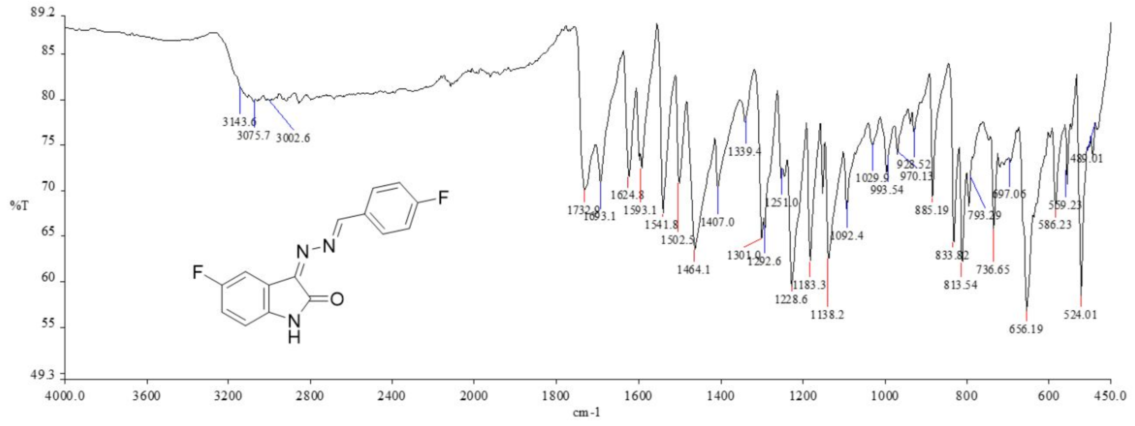

^{1}H NMR spectrum of compound **5**

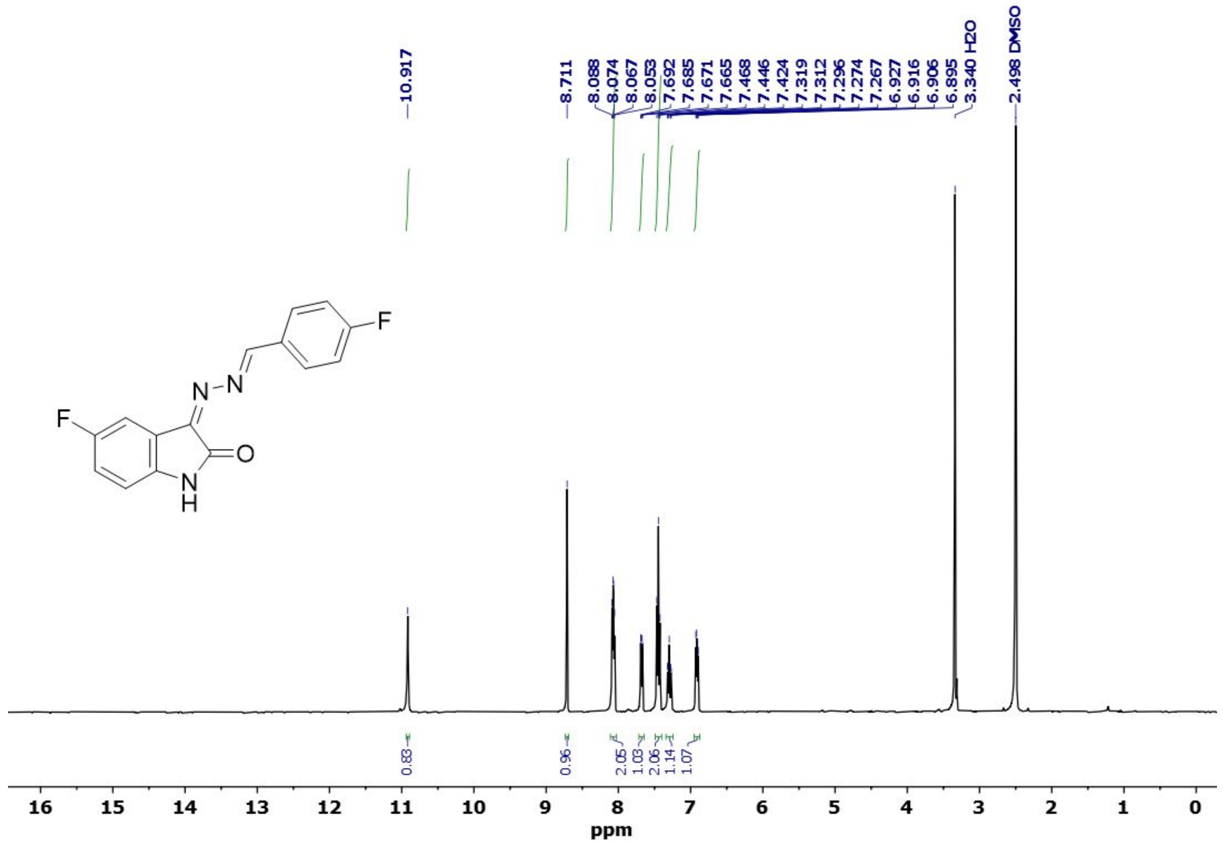

^{13}C NMR spectrum of compound **5**

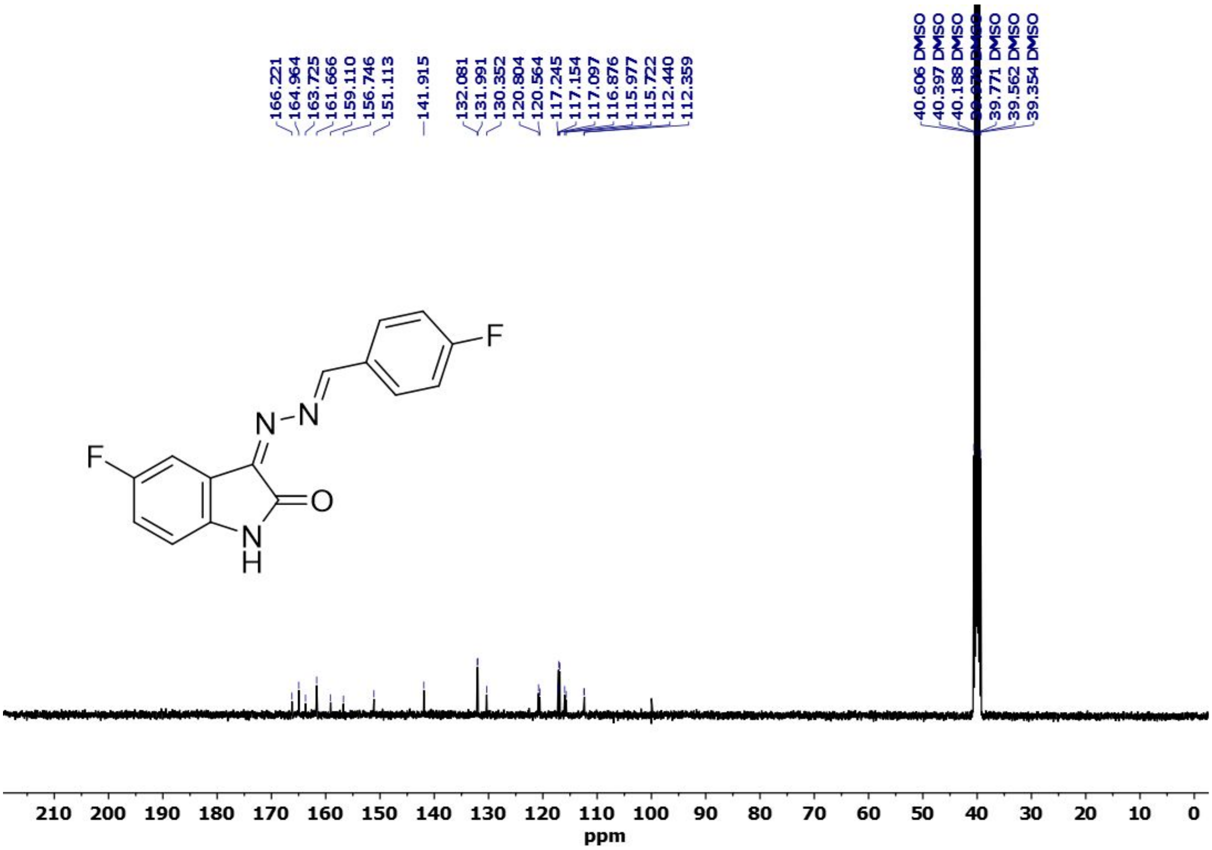

FT-IR spectrum of compound **6**

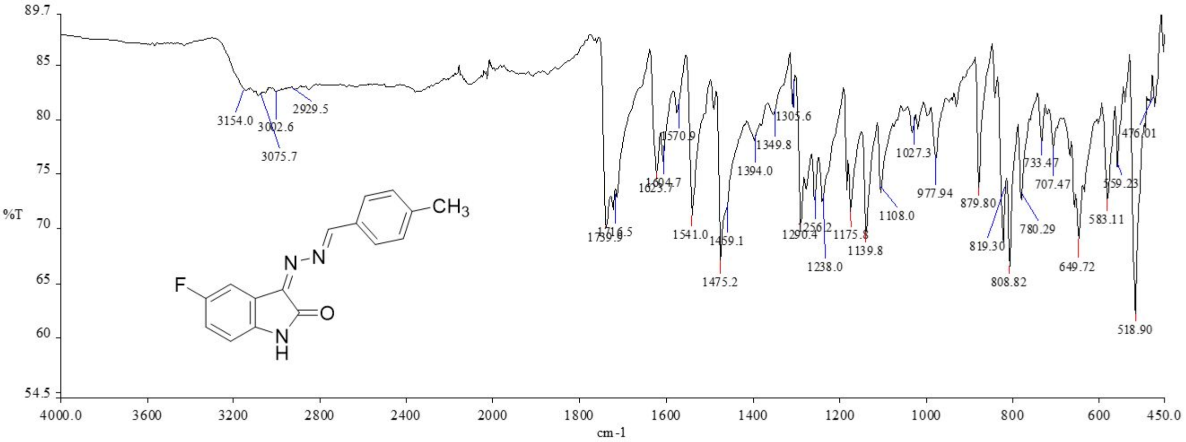

^{1}H NMR spectrum of compound **6**

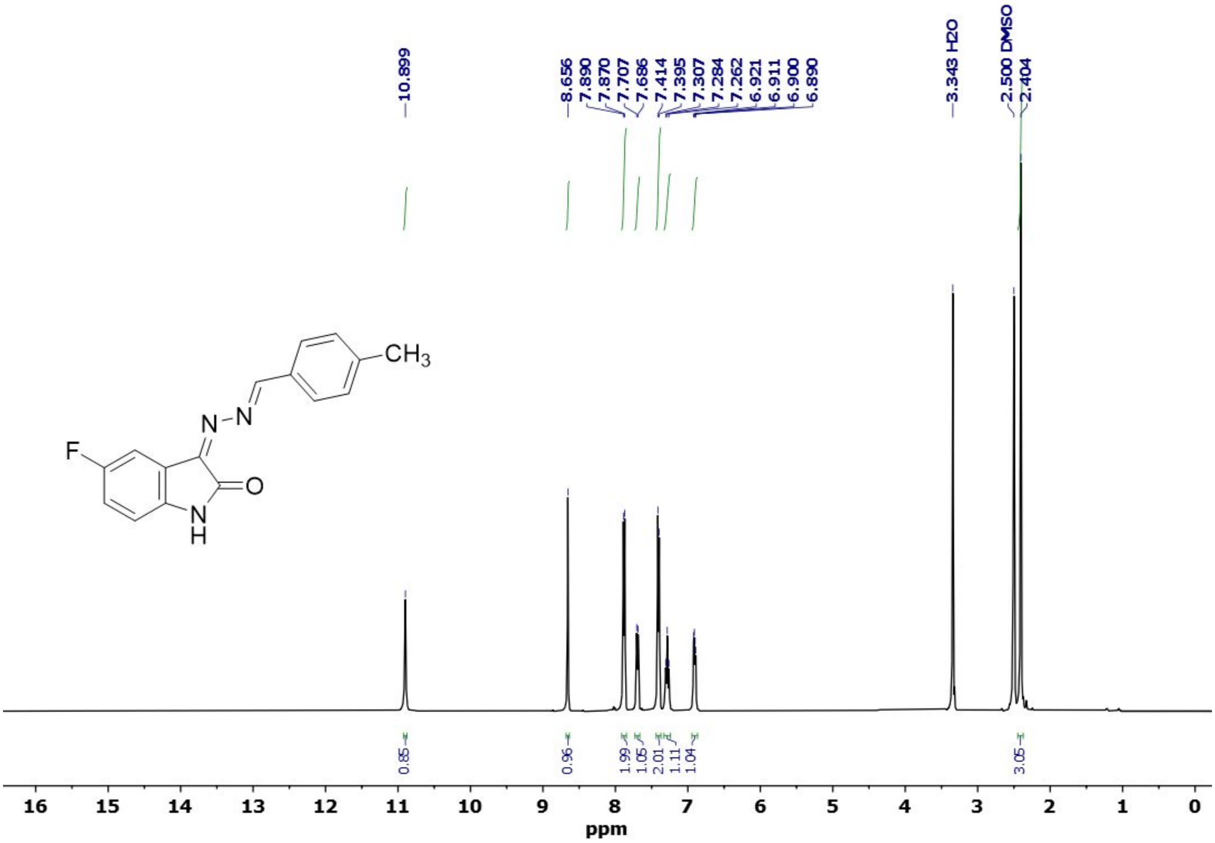

^{13}C NMR spectrum of compound **6**

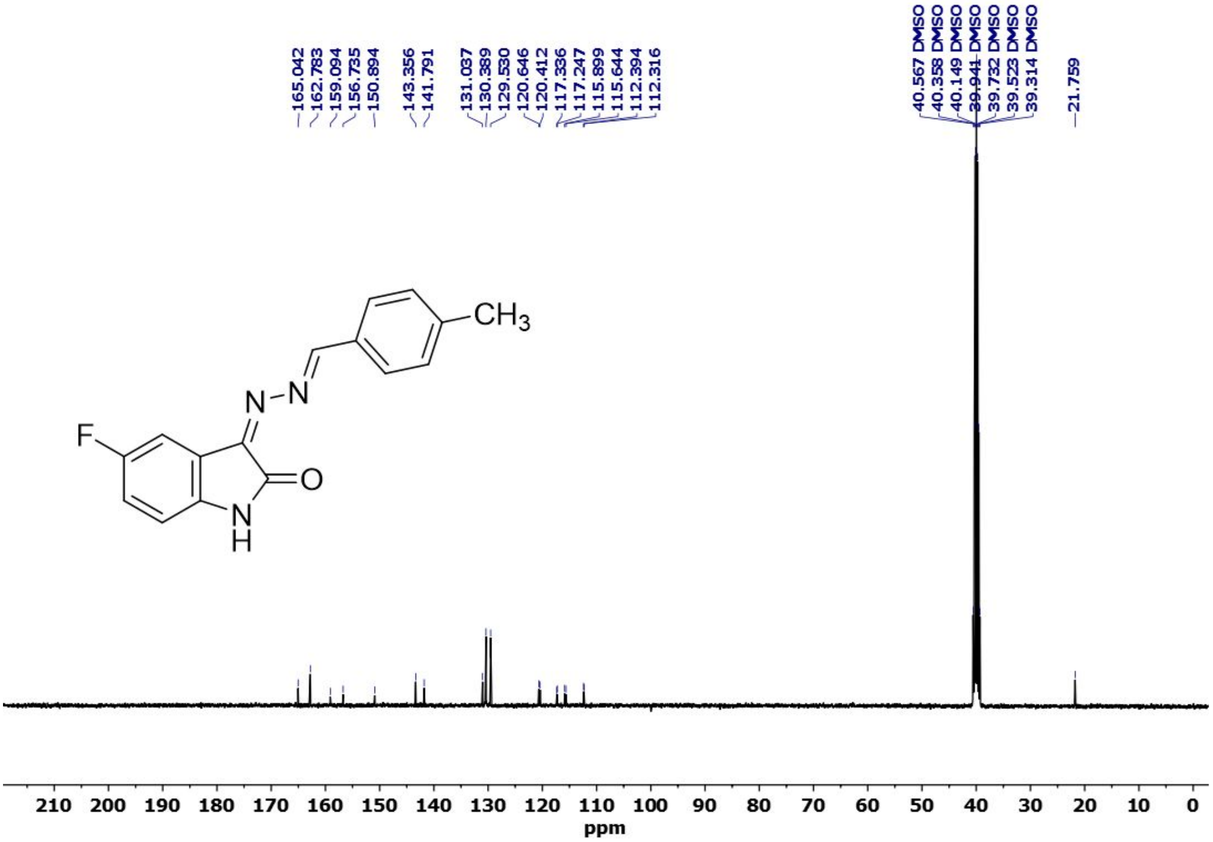

FT-IR spectrum of compound **7**

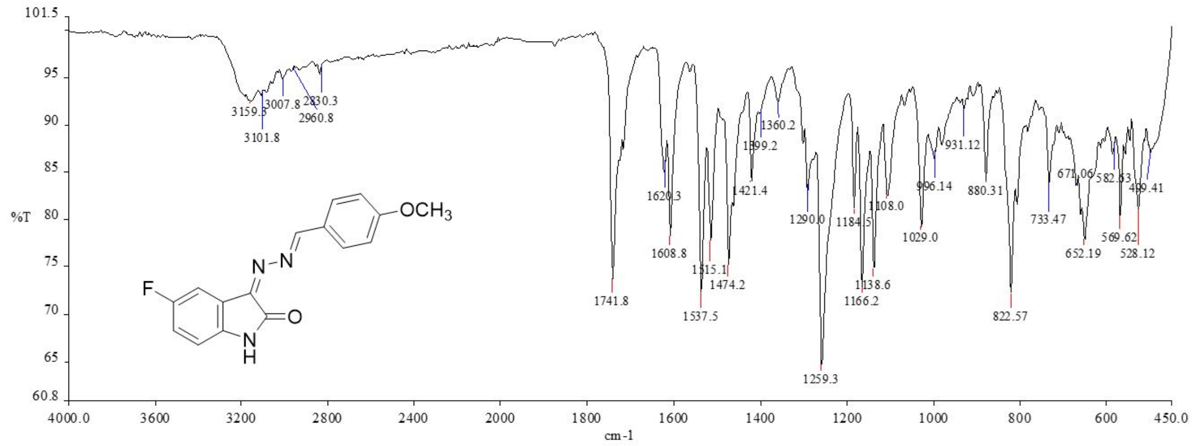

^{1}H NMR spectrum of compound **7**

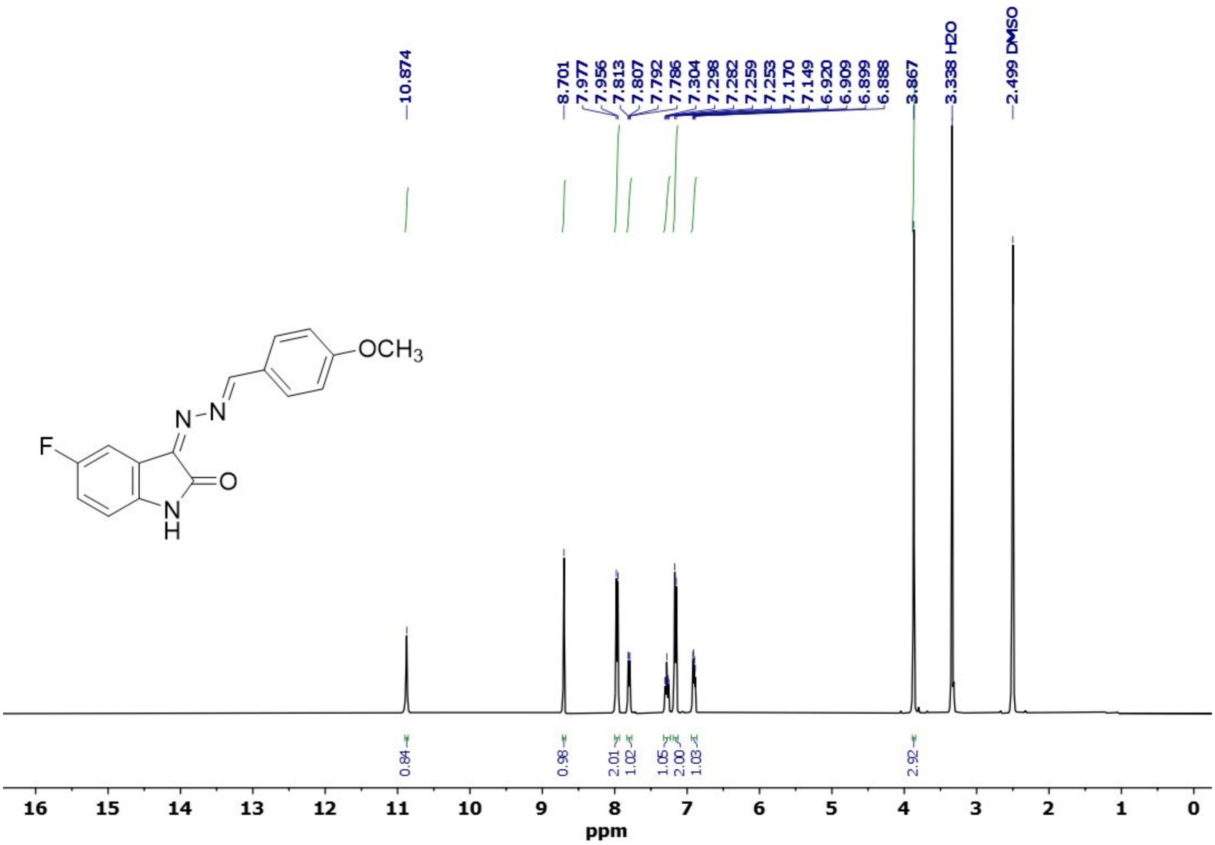

^{13}C NMR spectrum of compound **7**

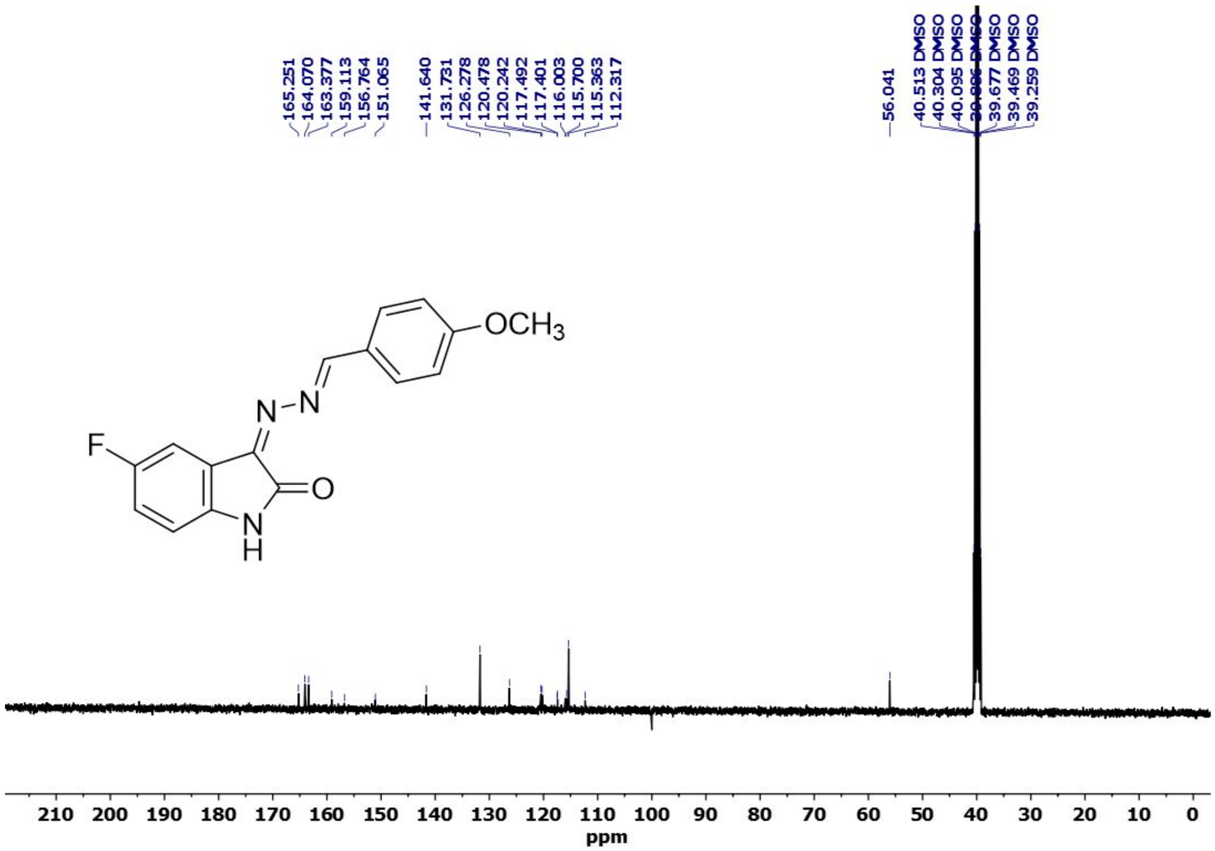

FT-IR spectrum of compound **8**

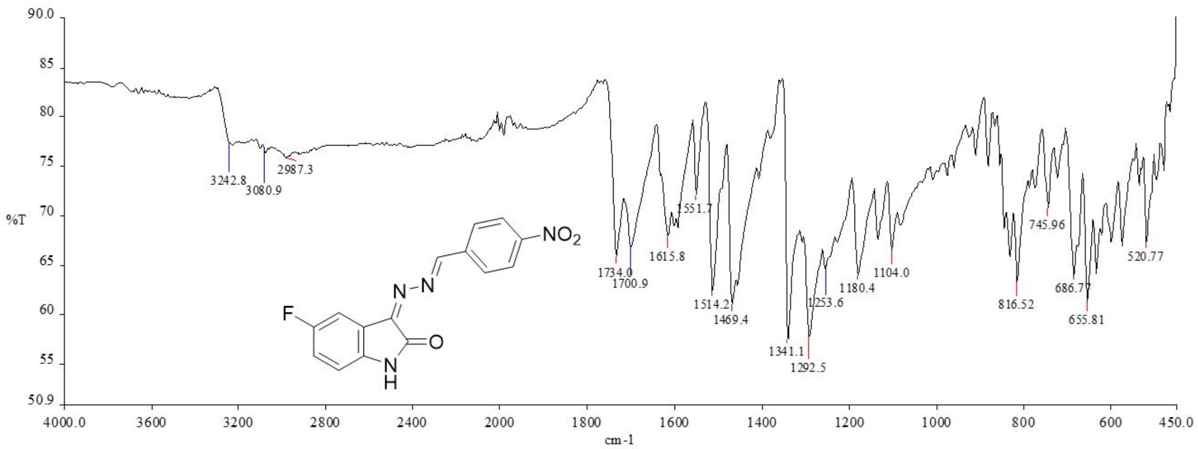

^{1}H NMR spectrum of compound **8**

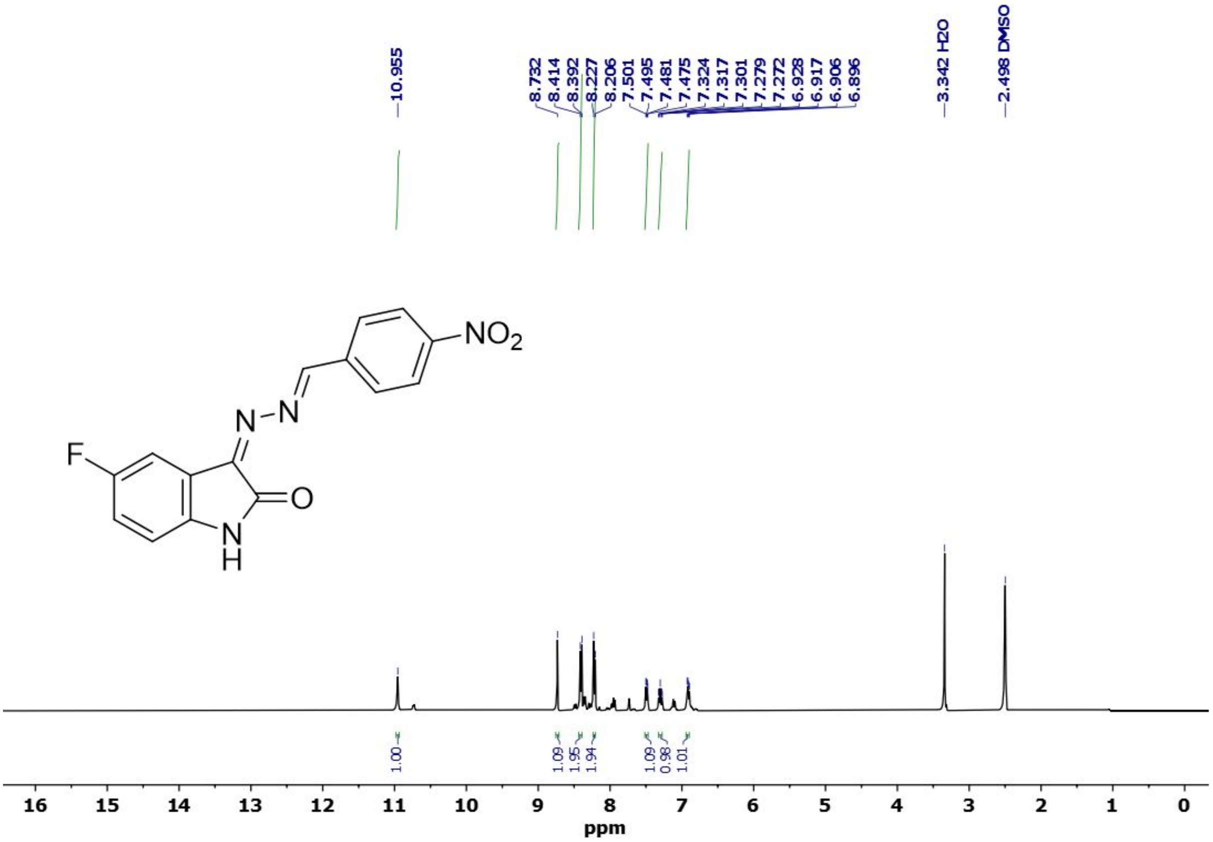

^{13}C NMR spectrum of compound **8**

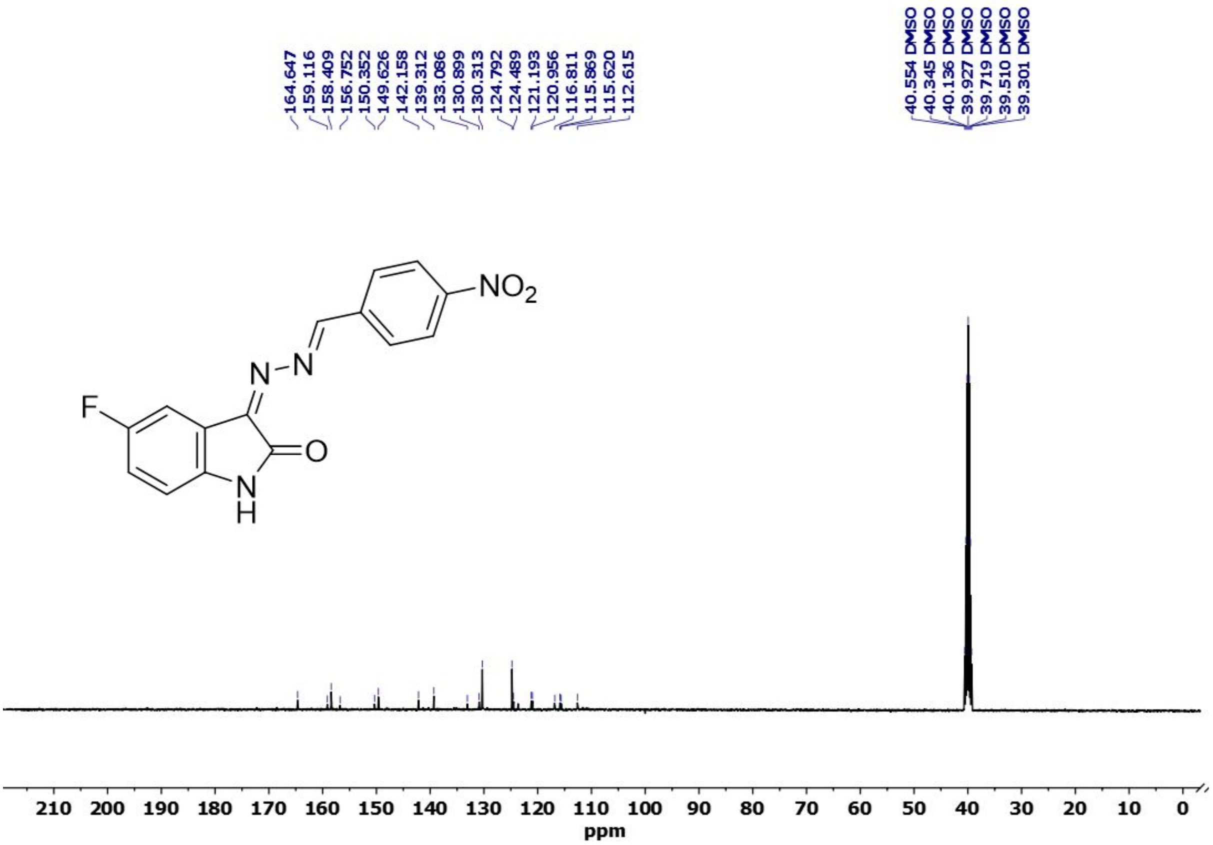

FT-IR spectrum of compound **9**

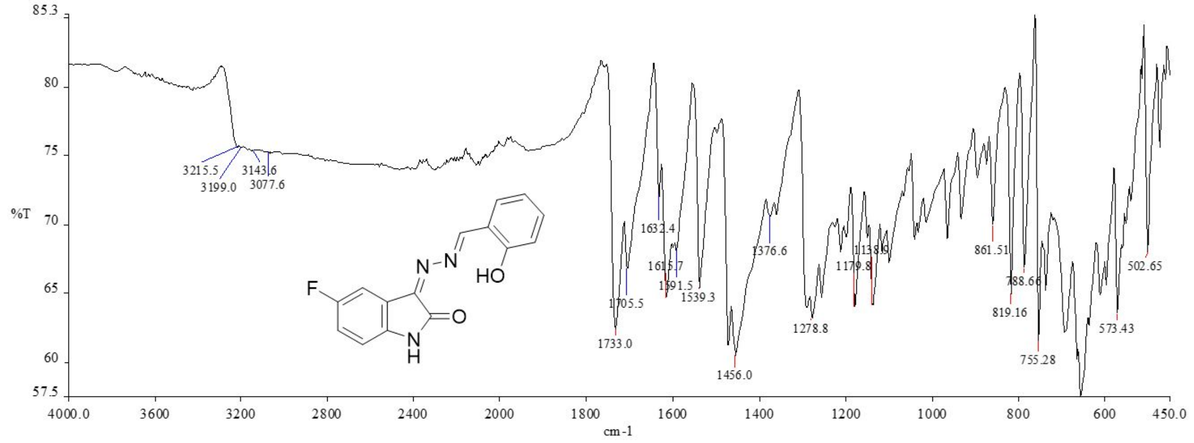

^{1}H NMR spectrum of compound **9**

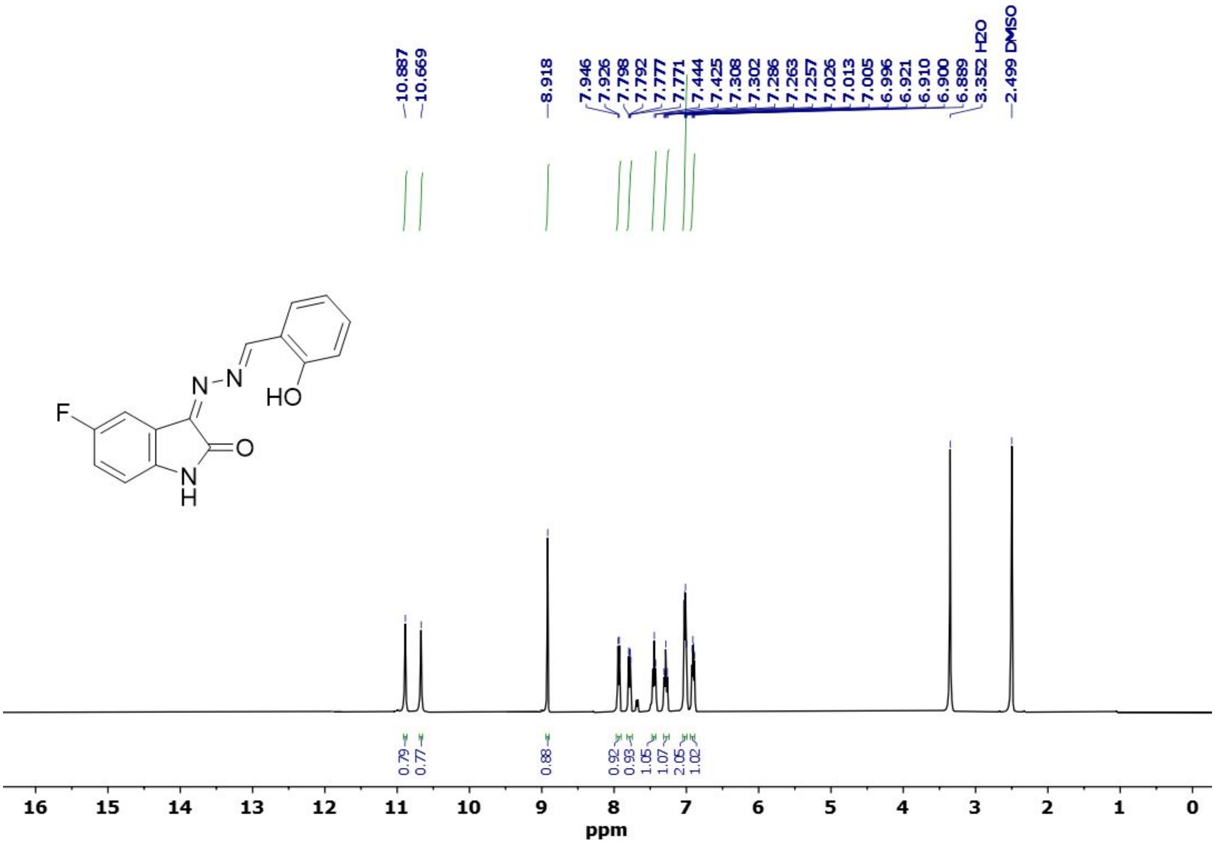

^{13}C NMR spectrum of compound **9**

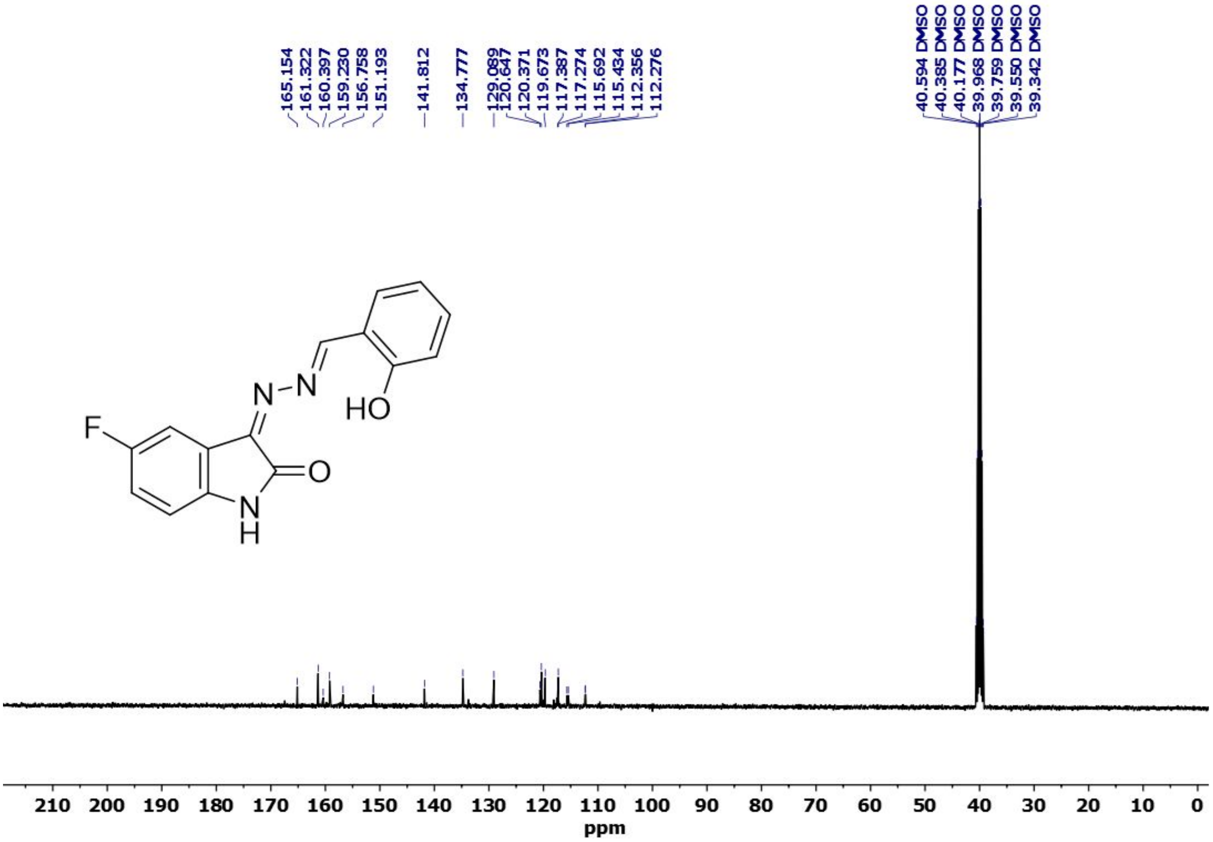

FT-IR spectrum of compound **10**

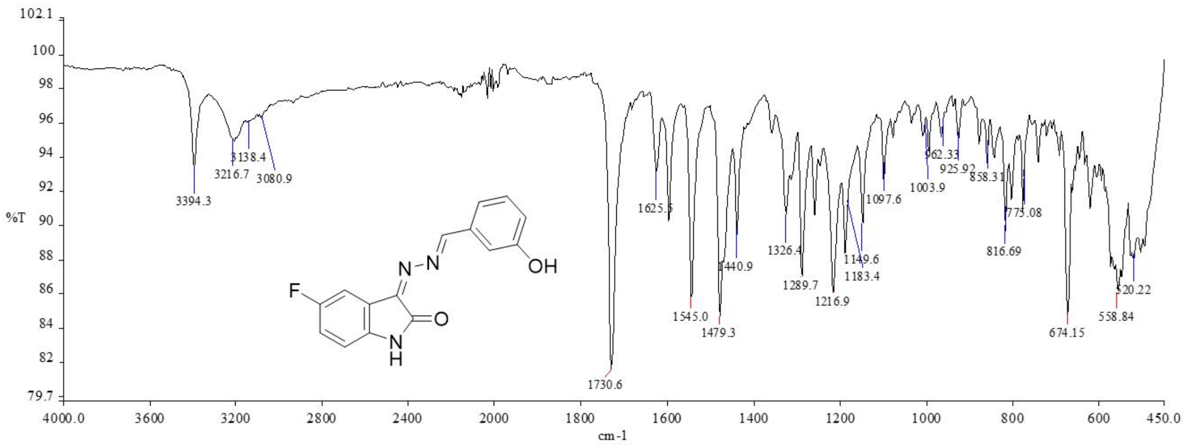

^{1}H NMR spectrum of compound **10**

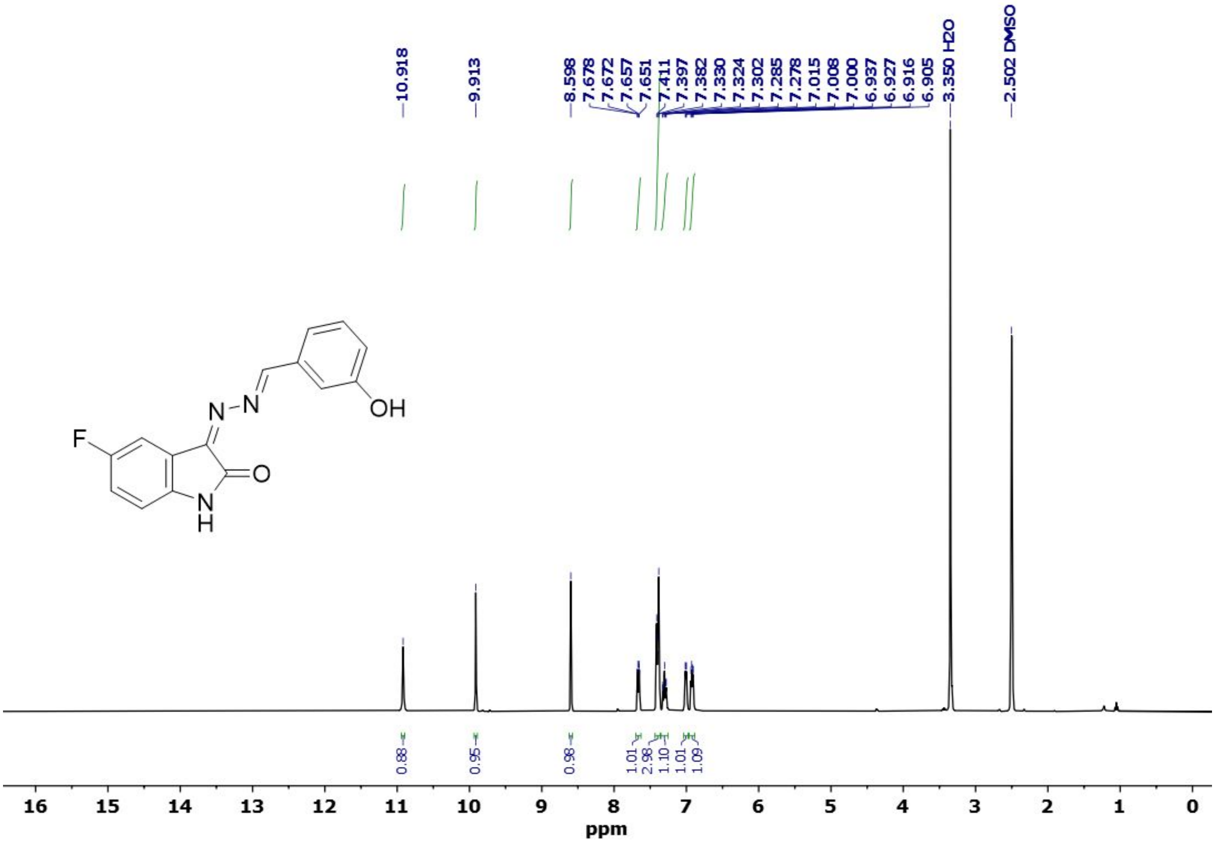

^{13}C NMR spectrum of compound **10**

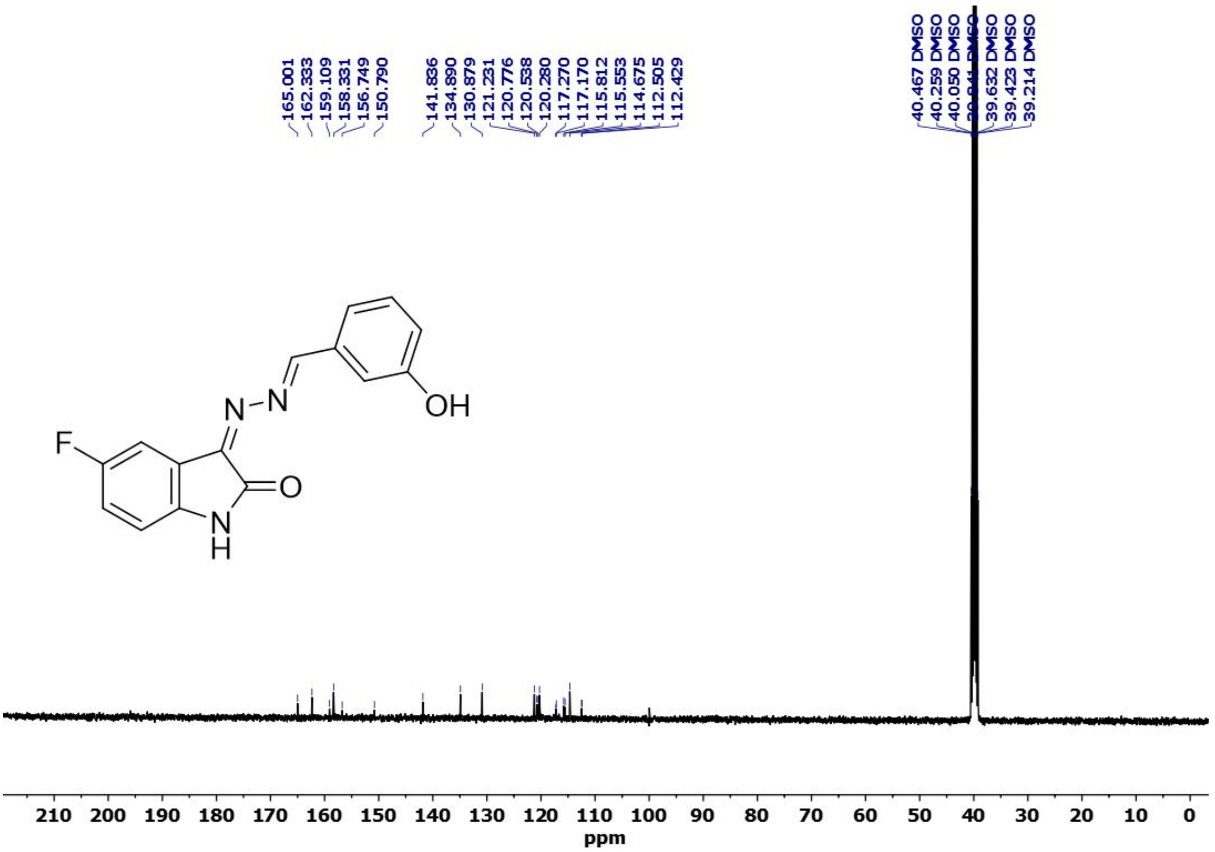

FT-IR spectrum of compound **11**

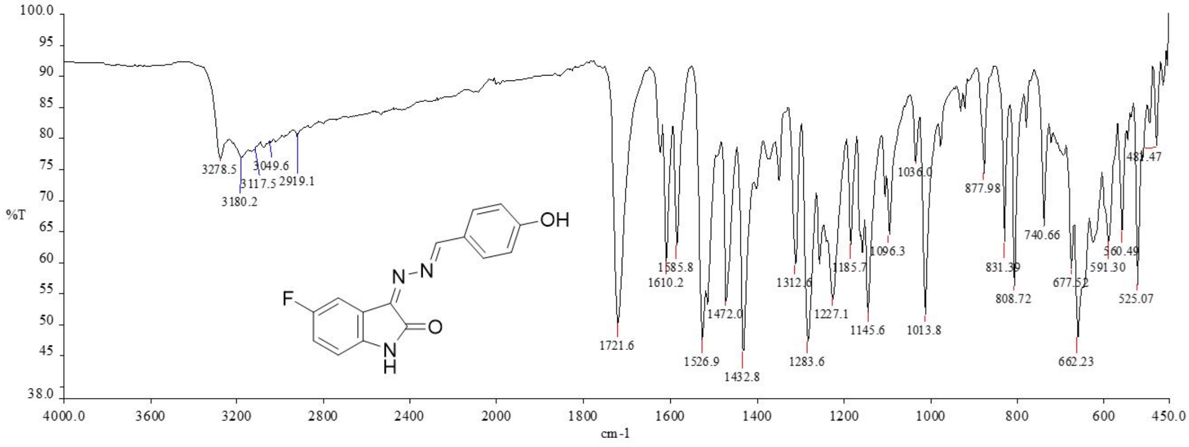

^{1}H NMR spectrum of compound **11**

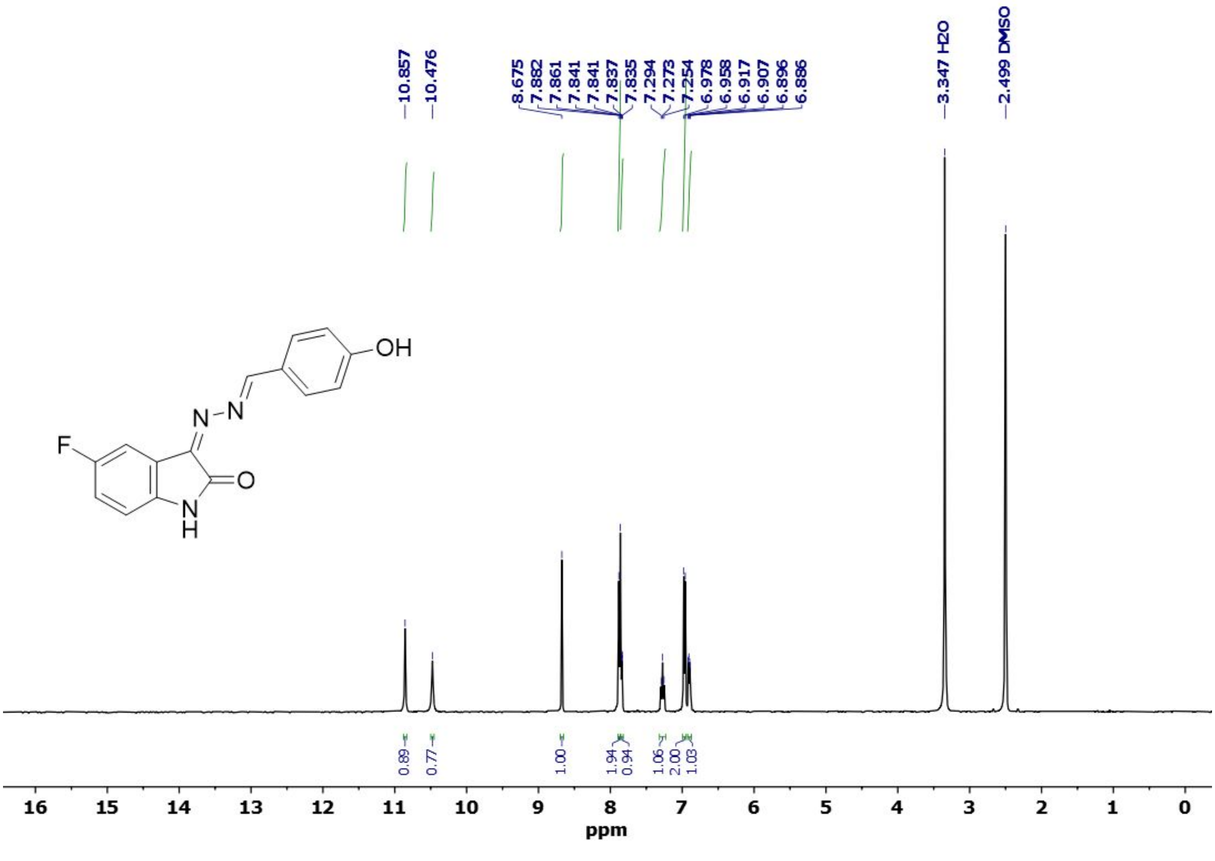

^{13}C NMR spectrum of compound **11**

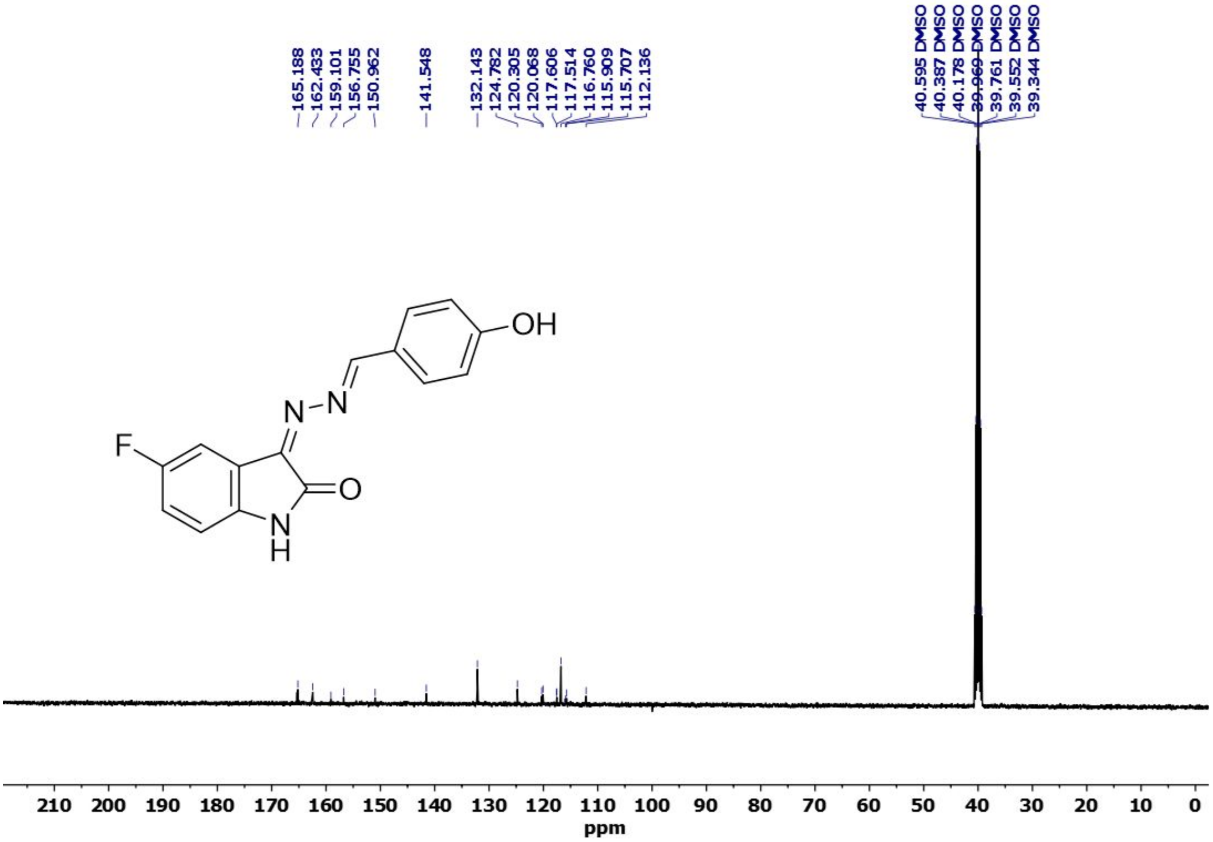

FT-IR spectrum of compound **12**

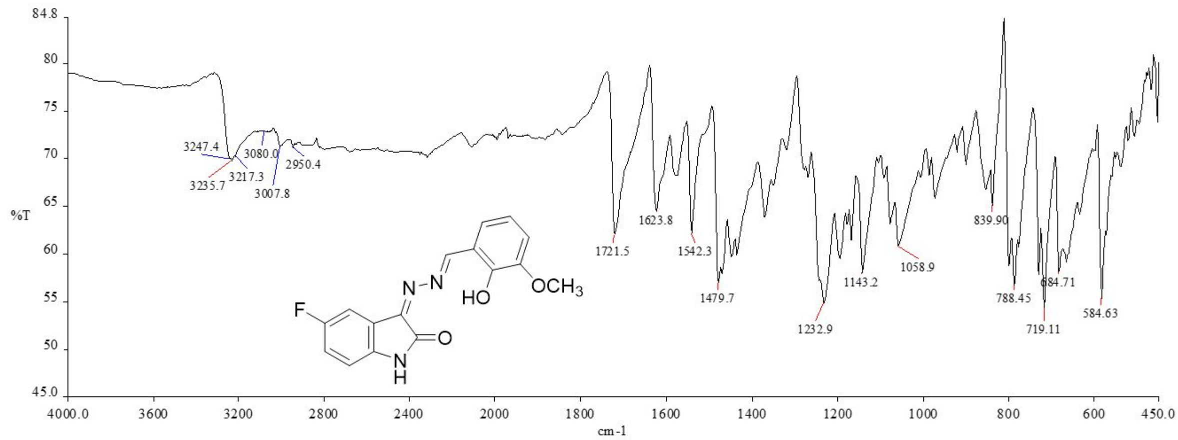

^{1}H NMR spectrum of compound **12**

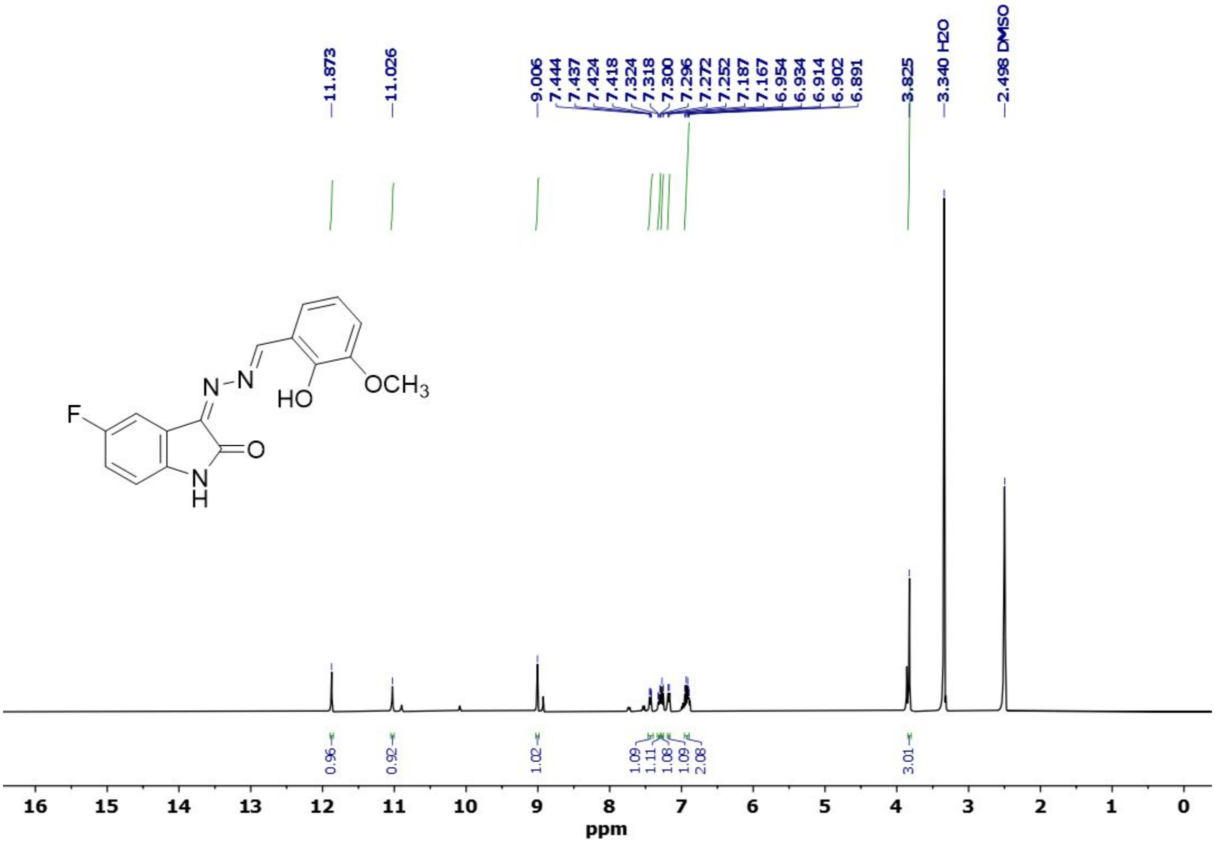

^{13}C NMR spectrum of compound **12**

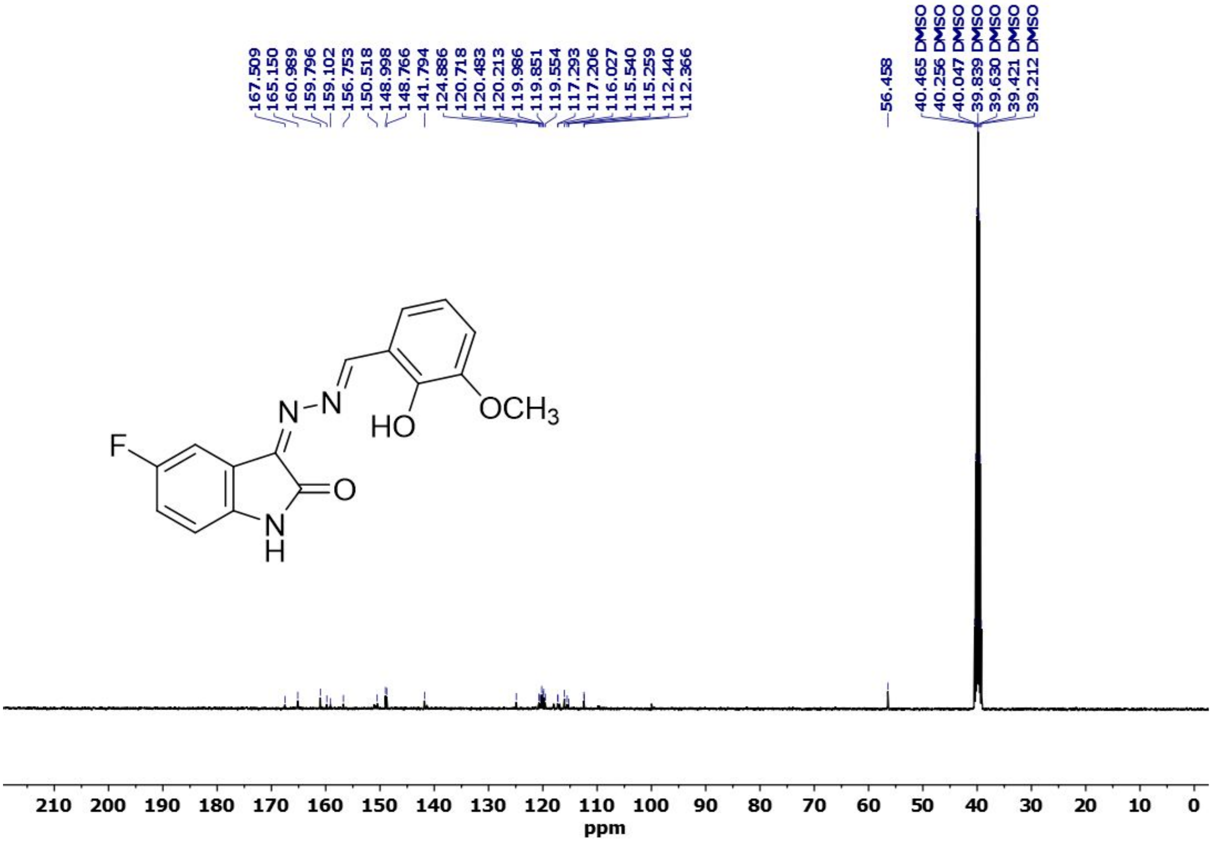

FT-IR spectrum of compound **13**

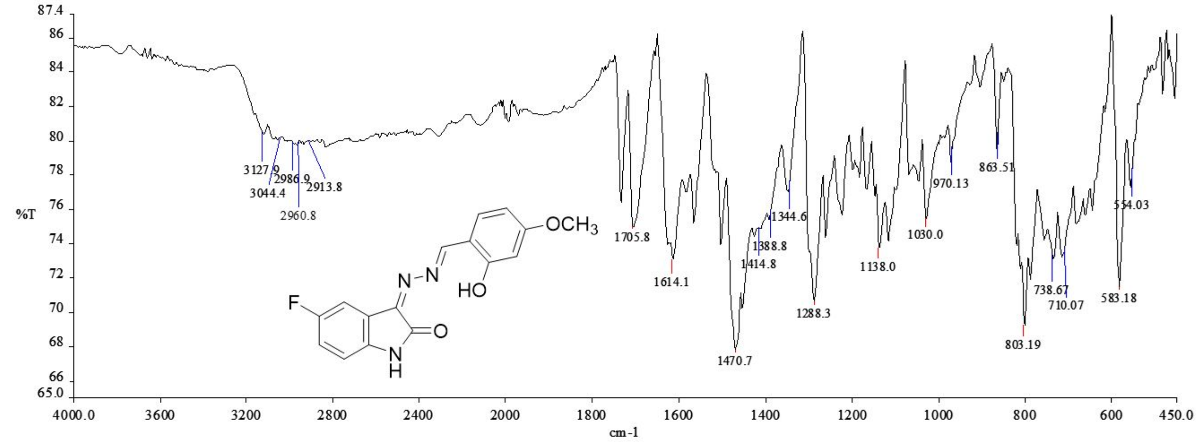

^{1}H NMR spectrum of compound **13**

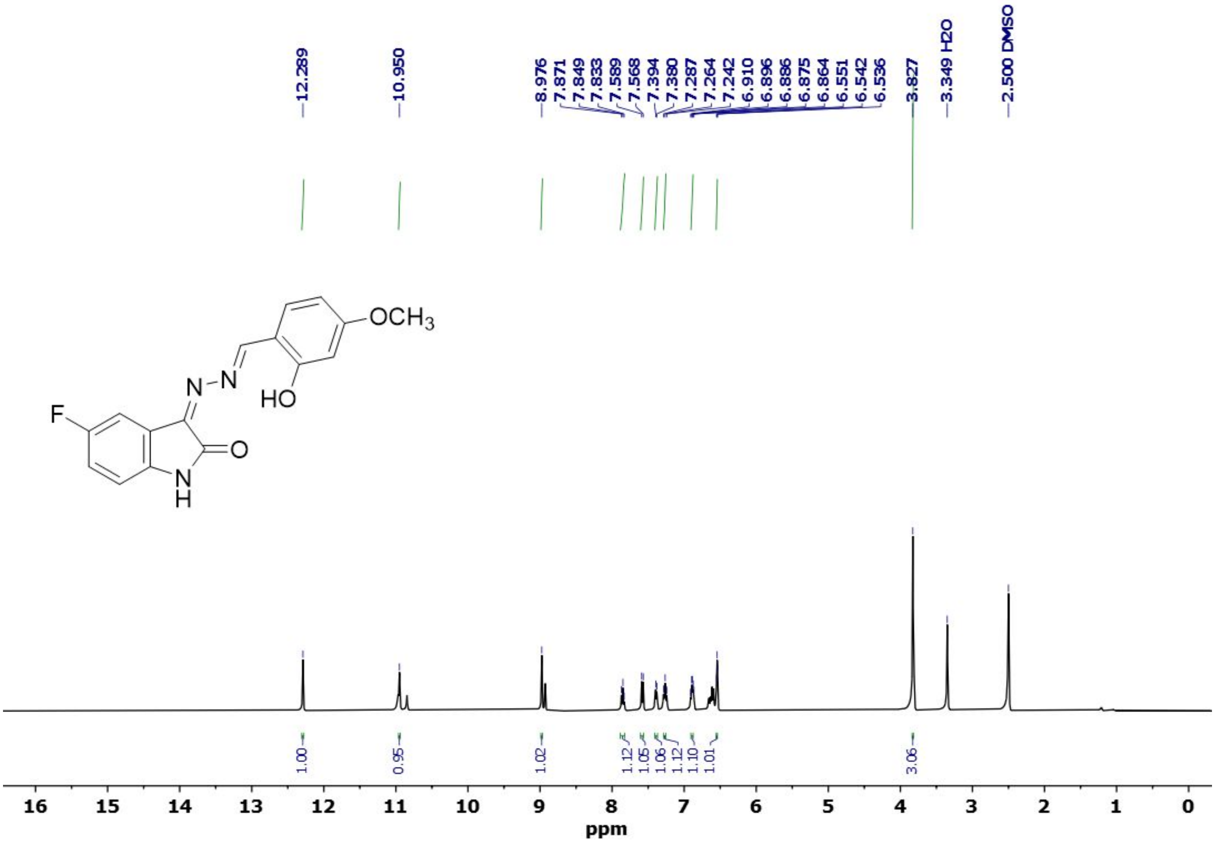

^{13}C NMR spectrum of compound **13**

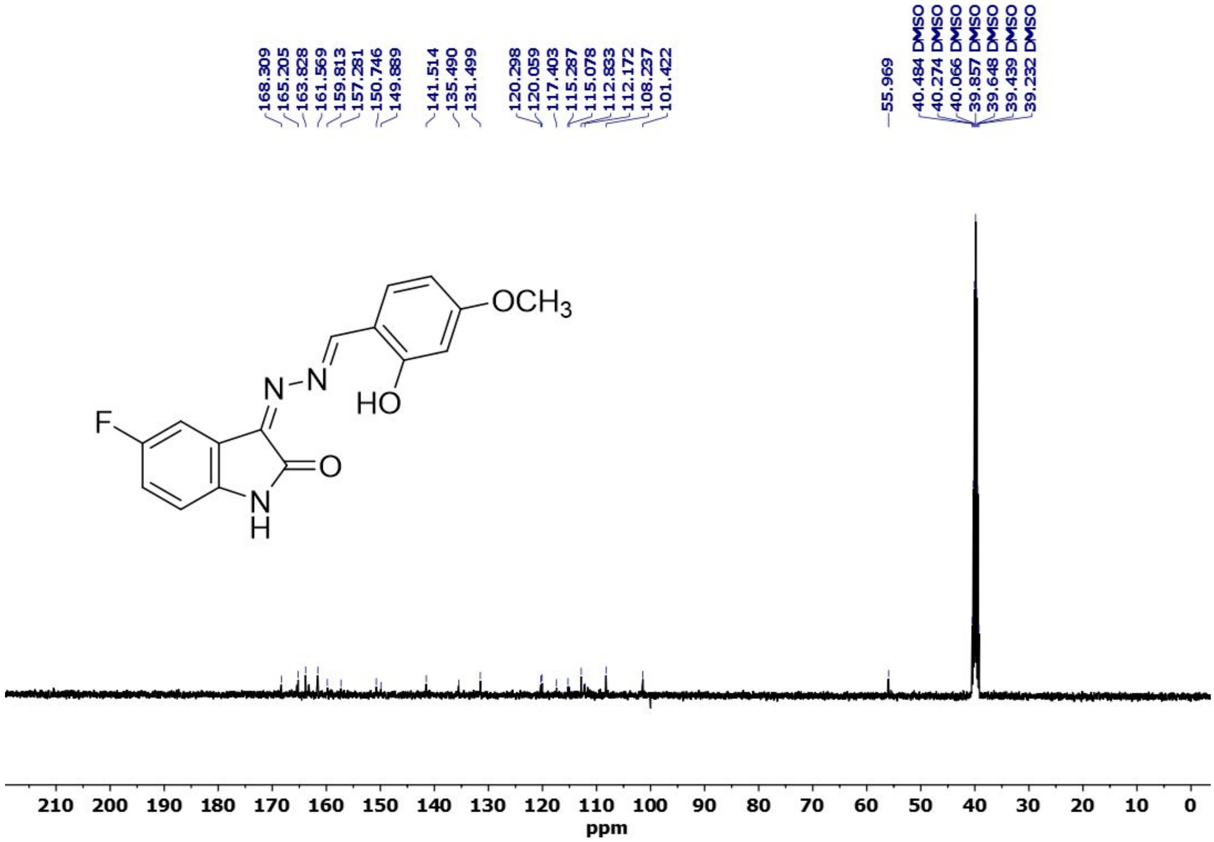

FT-IR spectrum of compound **14**

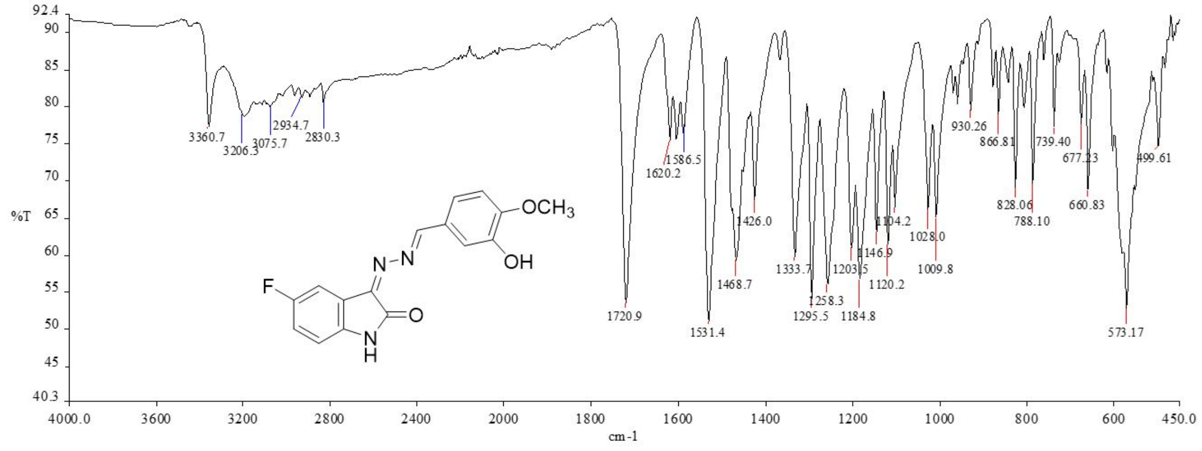

^{1}H NMR spectrum of compound **14**

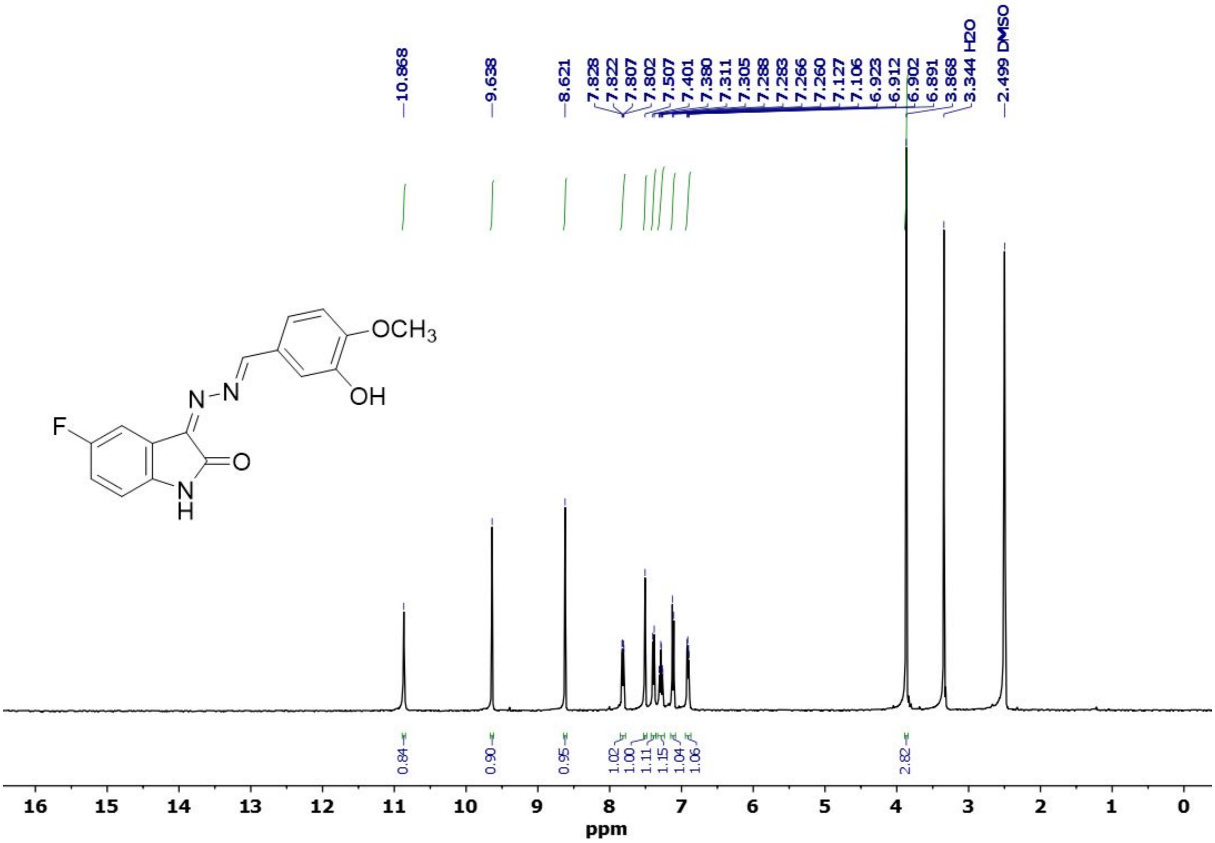

^{13}C NMR spectrum of compound **14**

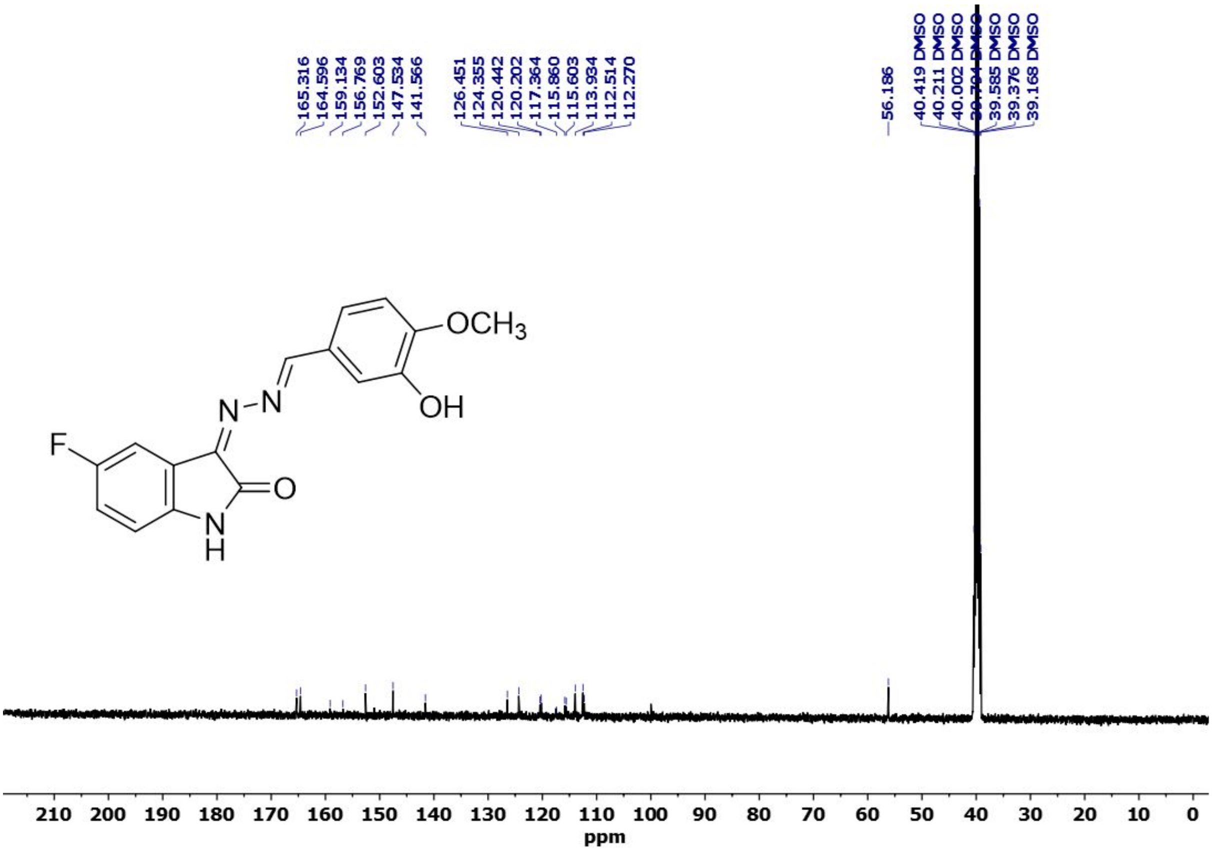

FT-IR spectrum of compound **15**

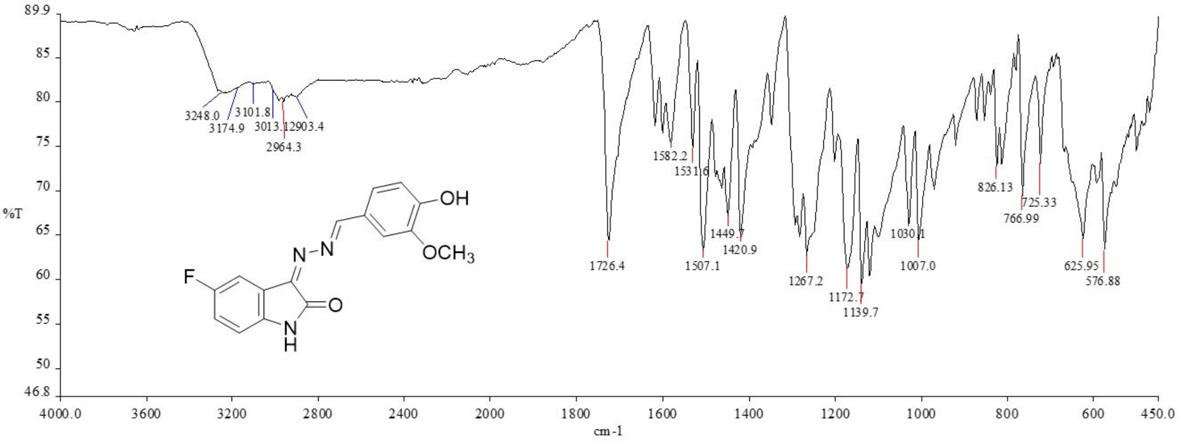

^{1}H NMR spectrum of compound **15**

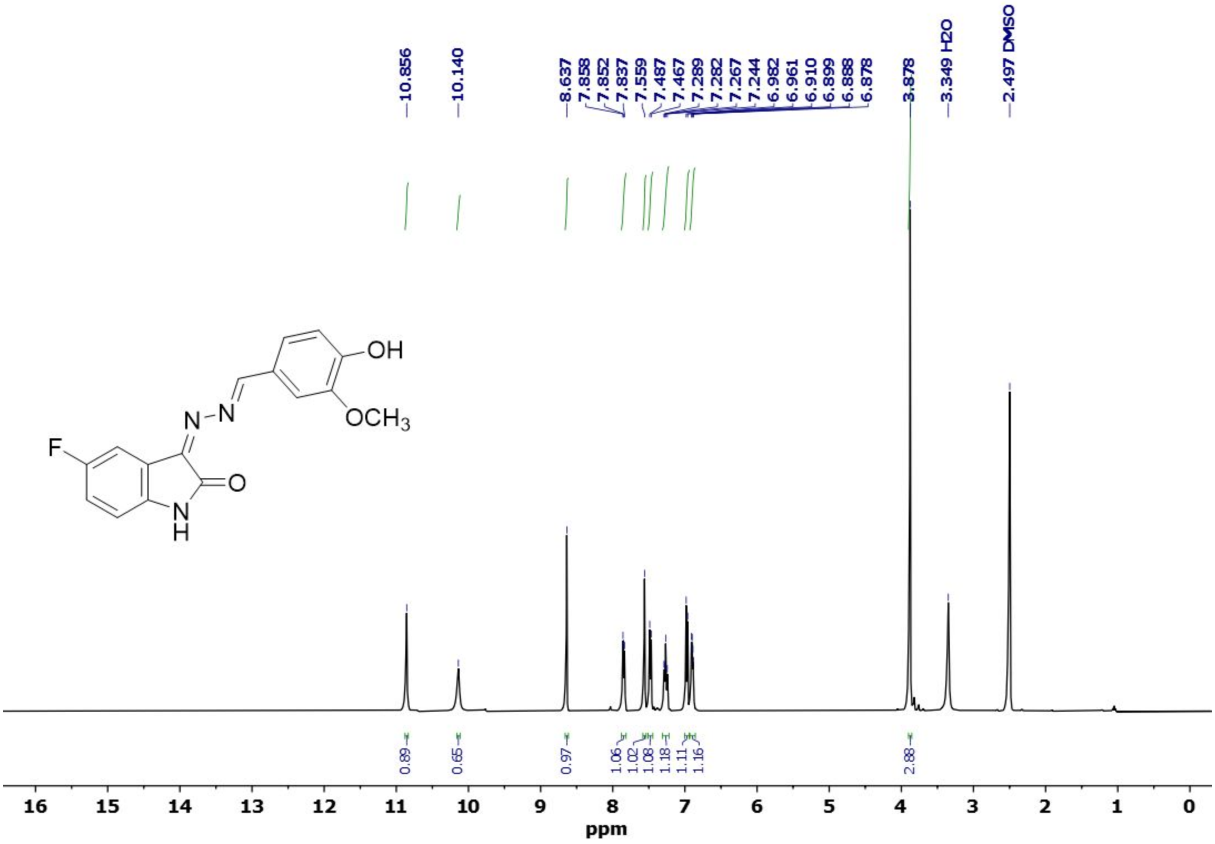

^{13}C NMR spectrum of compound **15**

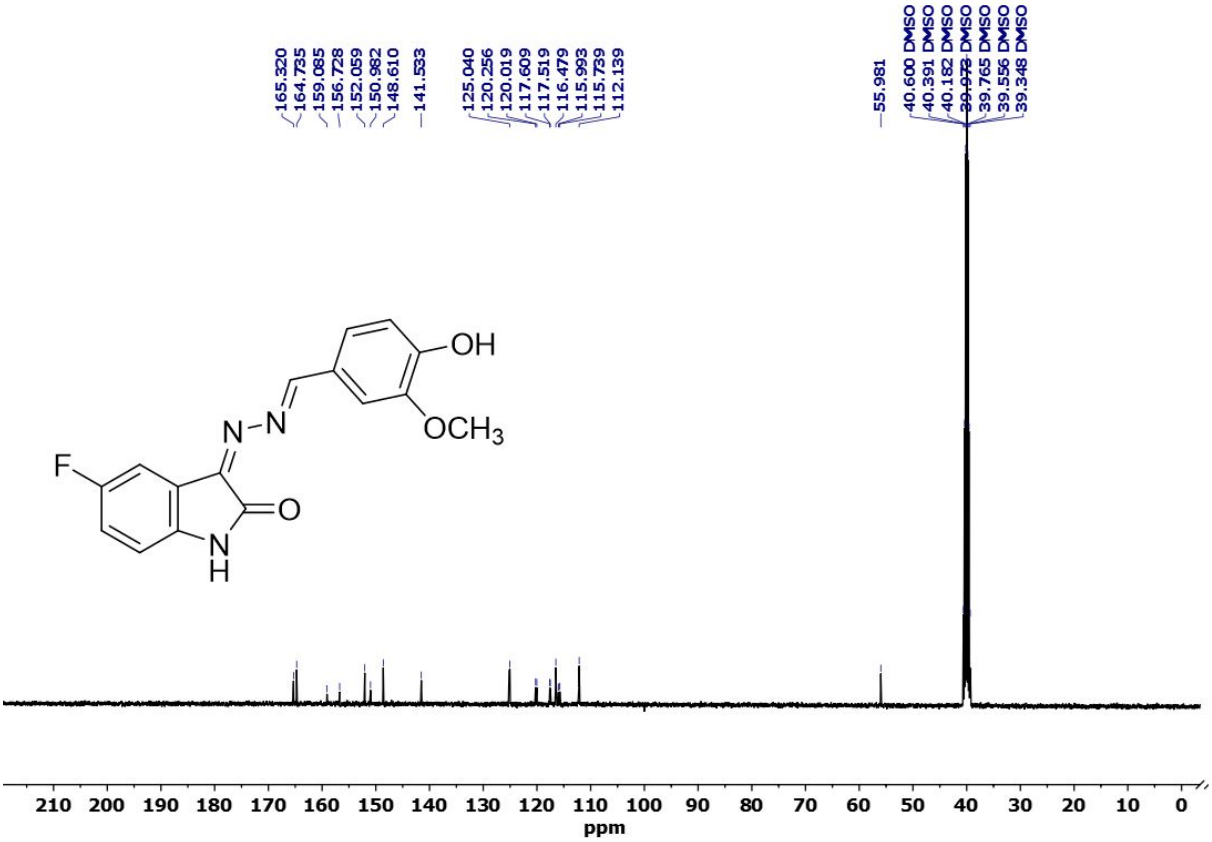

Supplement: Supplementary file 1 — ao4c03014_si_001.pdf [file ao4c03014_si_001.pdf]
